# Empowering photodynamic therapy with artificial intelligence: current trends and future directions

**DOI:** 10.3389/fonc.2026.1771804

**Published:** 2026-03-30

**Authors:** Avijit Paul, Marvin Xavierselvan, David Aebisher, Tomasz Kubrak, Dorota Bartusik-Aebisher, Srivalleesha Mallidi

**Affiliations:** 1 Department of Biomedical Engineering, Tufts University, Medford, MA, United States; 2 Department of Photomedicine and Physical Chemistry, Faculty of Medicine, Collegium Medicum, University of Rzeszów, Rzeszów, Poland; 3 Department of Biochemistry and General Chemistry, Faculty of Medicine, Collegium Medicum, University of Rzeszów, Rzeszów, Poland

**Keywords:** artificial intelligence, cancer therapy, clinical translation, deep learning, explainable AI, photodynamic therapy, photosensitizer design, treatment optimization

## Abstract

The evolution of photodynamic therapy (PDT), from ancient photomedicine practices to modern clinical applications, reflects its remarkable versatility in oncology and beyond. PDT relies on the interaction between photosensitizers, light, and tissue oxygen to generate reactive oxygen species that selectively destroy diseased cells. While the therapy has proven effective across various cancers and non-malignant conditions, tailoring treatment to individual patients remains challenging due to patient-specific variations in tissue optical properties, photosensitizer pharmacokinetics, and tumor heterogeneity. The rapid advancement of artificial intelligence (AI), including machine learning and deep learning, offers transformative opportunities to address these challenges through data-driven optimization and personalization. In this review, we examine how AI is being integrated across the PDT pipeline. We analyze AI-driven approaches for photosensitizer development, including quantitative structure-activity relationship modeling, graph neural networks for property prediction, and generative models for *de novo* molecular design. We examine machine learning applications in nanoparticle-based drug delivery systems, encompassing synthesis optimization, nano-bio interaction prediction, and stimuli-responsive release modeling. The review further explores AI integration in treatment planning through real-time tissue optical property estimation, and in clinical decision-making through treatment response monitoring and outcome prediction using multimodal imaging data. We critically assess current limitations, including small dataset challenges, model interpretability concerns, and the gap between preclinical research and clinical translation. Finally, we outline future directions, including federated learning, explainable AI, and regulatory considerations. This review aims to bridge the AI and PDT communities, providing a roadmap for improved patient outcomes.

## 1 Introduction

Photodynamic therapy (PDT) is a minimally invasive treatment modality that combines a photosensitizing drug, light, and tissue oxygen to selectively destroy malignant and diseased cells (1–3). Since the first FDA approval in 1995 (4, 5), PDT has demonstrated efficacy across multiple cancer types and non-malignant conditions (6–8). Among established cancer treatments, PDT offers distinct advantages: spatial selectivity through localized light delivery, minimal systemic toxicity, and repeatability without cumulative dose limitations (2, 6). Yet clinical adoption has not matched its therapeutic potential. The fundamental barrier is not the therapy itself but our inability to tailor treatments to individual patients, a challenge that artificial intelligence (AI) is uniquely positioned to address.

Although PDT has ancient roots where civilizations like Egypt, Greece, and India used plant extracts and sunlight to treat skin ailments (9, 10), its modern scientific foundation dates to 1900 when Raab first observed light-activated cytotoxicity. Clinical development accelerated after Dougherty’s trials in the 1970s established therapeutic efficacy (4, 11–14). FDA approval of Photofrin in 1995 marked a milestone but also established standardized treatment protocols that persist today (5). Subsequent generations of photosensitizers (PS), including phthalocyanines, chlorins, and improved porphyrin derivatives, addressed early limitations such as prolonged photosensitivity while expanding clinical applications (15). Recent advances include integration with imaging modalities for real-time monitoring, nanoparticle-based delivery systems, and combination approaches with immunotherapy and chemotherapy (16–22). Yet despite these technological advances, PDT protocols remain largely standardized rather than individualized.

Despite this progress, the gap between PDT’s potential and its clinical reality stems from biological variability that current protocols cannot accommodate. Tissue optical properties, including absorption and scattering coefficients, vary substantially between patients and even within the same tumor, with resulting variations in delivered fluence that can exceed 100% in some cases (23). A light dose that achieves a therapeutic effect in one patient may be insufficient or excessive in another. PS pharmacokinetics add another layer of uncertainty: uptake rates, biodistribution patterns, and clearance kinetics differ based on tumor type, vasculature, and individual patient factors (24). Tumor heterogeneity compounds these challenges. Oxygen concentration, critical for the photodynamic reaction, varies spatially within tumors and changes during treatment as the photodynamic process itself consumes oxygen (18). Hypoxic regions may receive subtherapeutic doses while well-oxygenated areas experience adequate treatment. Current clinical protocols rely largely on standardized parameters, such as fixed light doses and standard drug-light intervals, that ignore this patient-specific variability (25). The consequence is highly variable response rates depending on the indication and patient selection. Improving outcomes requires moving beyond one-size-fits-all protocols toward adaptive, individualized treatment planning.

**Figure 1:**
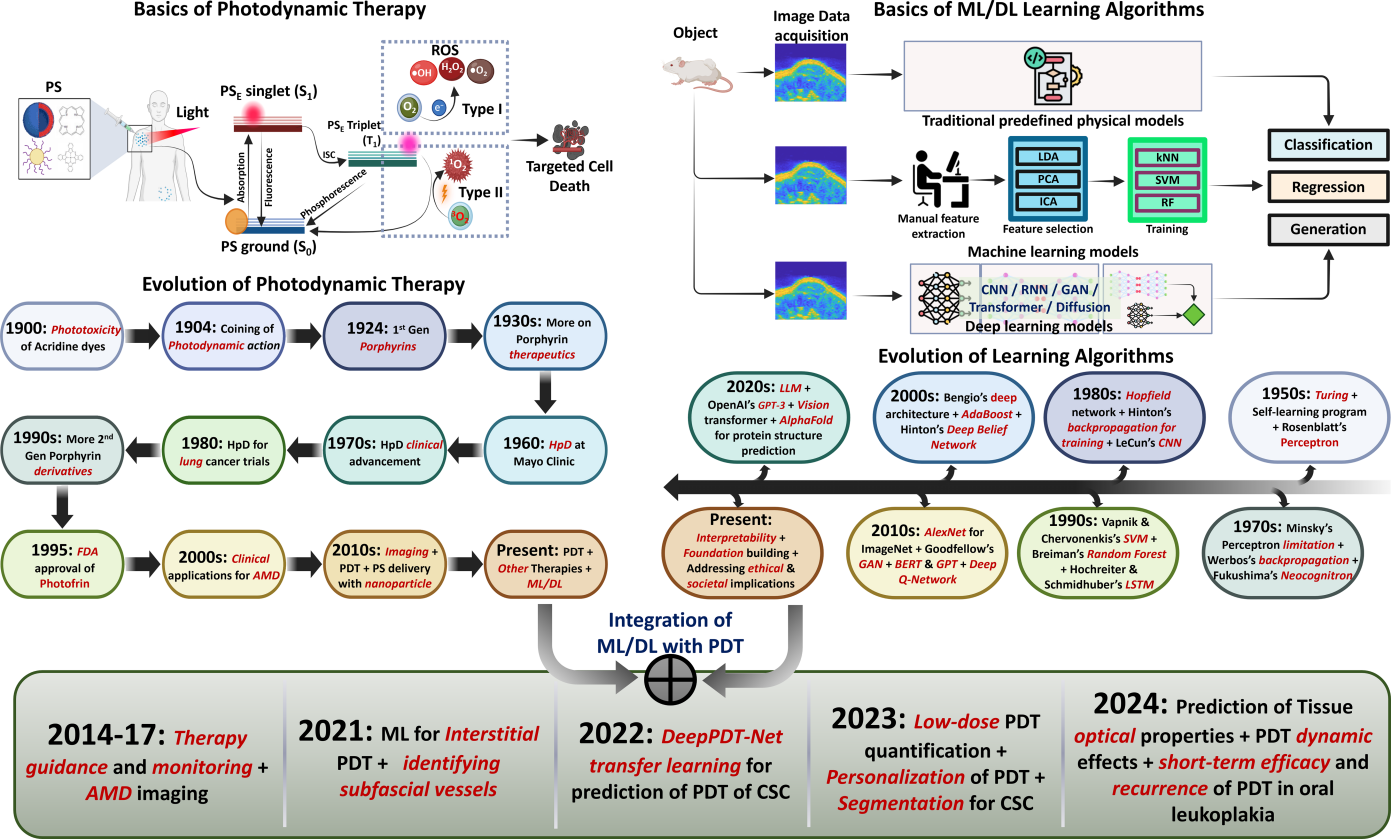
Evolution of PDT and ML/DL. (Top left) PDT mechanism illustrated via the Jablonski diagram: photon absorption promotes the PS from ground state (S_0_) to excited singlet state (S_1_), followed by ISC to the triplet state (T_1_), enabling Type I and Type II photochemical reactions that generate ROS for targeted cell death. (Top right) Comparison of traditional predefined models, ML requiring manual feature extraction, and DL with automatic feature learning. (Middle) Historical timelines trace PDT development from Raab’s discovery of photosensitization (1900) through FDA approval (1995) to current nanoparticle-based and combination therapies, alongside the evolution of learning algorithms from early perceptrons (1950s) through DL architectures and foundation models (2020s). (Bottom) Key milestones in AI-PDT integration (2014–2024), highlighting advances in therapy guidance, treatment prediction, and personalization.

Machine learning (ML) is a subset of AI in which algorithms learn patterns from data rather than following explicitly programmed rules (26–28). Traditional ML approaches, such as support vector machines (SVMs), K-nearest neighbors (KNN), principal component analysis (PCA), Naive Bayes, random forests, and gradient boosting, excel in structured data analysis but require domain experts to manually define relevant features (29–36) (**Figure 1**). Deep learning (DL) overcomes this limitation through neural networks with multiple hierarchical layers that automatically extract features from raw inputs. Convolutional neural networks have revolutionized medical image analysis, while recurrent architectures and transformers capture temporal and sequential patterns (37–41). These methods have driven advances in medical imaging, drug discovery, and clinical decision support (42–47), with particular success in radiation therapy treatment planning (48–51). PDT presents a high-dimensional parameter space spanning tissue optical properties, PS concentration, oxygen levels, and light dose (25), creating optimization challenges ideally suited to ML approaches. Pattern recognition from multimodal imaging enables real-time treatment adaptation, while structure-property prediction accelerates PS discovery. The computational tractability of PDT physics, combined with growing imaging datasets, positions this modality for significant AI-driven advances.

AI showed transformative potential in radiotherapy treatment planning (52) and accelerated drug discovery pipelines (46), yet PDT scarcely appears in the broader AI-oncology literature (53, 54). Conversely, PDT reviews address computational modeling such as Monte Carlo (MC) simulations and finite element methods (55), but rarely engage with modern ML techniques. This gap is significant: PDT’s high-dimensional parameter space, encompassing tissue optics, PS pharmacokinetics, oxygen dynamics, and light dosimetry, is precisely the type of complex optimization challenge where AI excels. Moreover, critical areas remain unexplored: transfer learning for small datasets, federated approaches for multi-institutional collaboration, and regulatory pathways for clinical implementation. This review bridges the AI and PDT communities by providing the first comprehensive survey of ML and DL applications across the complete PDT pipeline, from PS discovery and nanoparticle design through treatment planning, response monitoring, and outcome prediction. Beyond cataloging existing work, we critically assess translational barriers, including small dataset challenges, model interpretability, and regulatory pathways that must be addressed for clinical implementation. We conclude with future research directions for realizing AI-empowered personalized PDT.

## 2 Photodynamic therapy: mechanisms and applications

### 2.1 PDT: photophysics and mechanisms

**Figure 2:**
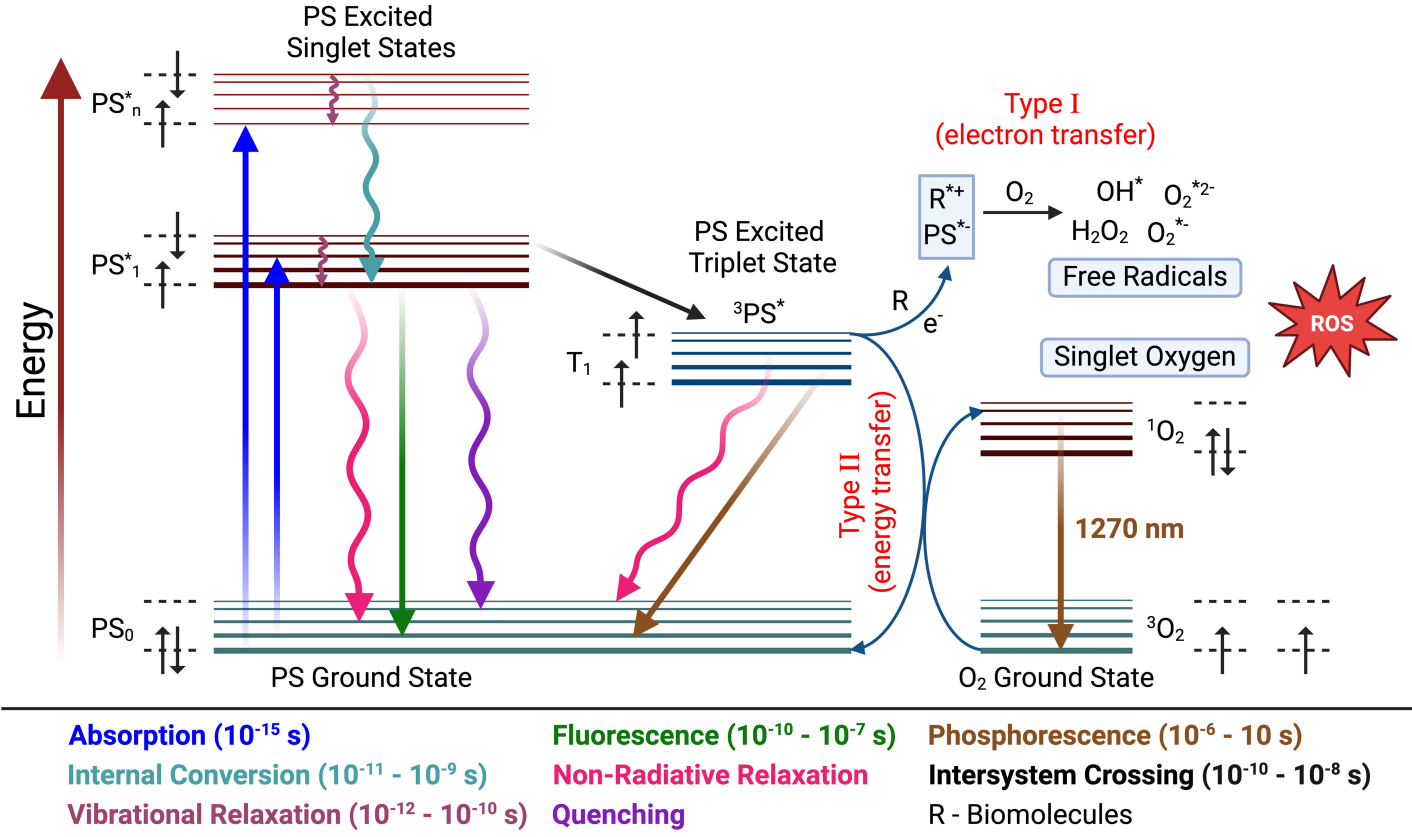
Jablonski diagram illustrating the photophysical processes underlying PDT. Upon light absorption (10^–15^ s), the PS is excited from its ground state ( $PS_{0}$) to higher singlet excited states ( $PS_{1}^{*},\;\;PS_{n}^{*}$). The molecule can return to the ground state via fluorescence (10^-10^ - 10^–7^ s) or non-radiative pathways including internal conversion and vibrational relaxation. Alternatively, ISC (10^-10^–10^-8^ s) populates the longer-lived triplet state (^3^ $PS^{*}$), which enables therapeutic ROS generation through two competing mechanisms. In Type I reactions, electron transfer to biomolecules (R) produces free radicals ( $O_{2}^{-},\;OH•,\;H_{2}O_{2}$). In Type II reactions, energy transfer to ground-state molecular oxygen (^3^ $O_{2}$) generates singlet oxygen (^1^ $O_{2}$), detectable by its characteristic 1270 nm phosphorescence. Both pathways converge on ROS-mediated cytotoxicity. Created in BioRender. Mallidi, S. (2026) https://BioRender.com/dkz7qn4.


PDT, at its core, operates on a deceptively simple premise: light activates a drug to destroy diseased tissue. Yet beneath this simplicity lies a precisely orchestrated sequence of photophysical events, oxygen dynamics, and biological responses that collectively determine therapeutic success. Understanding these mechanisms is essential, not only for optimizing treatment protocols but also for identifying the parameters that machine learning models can leverage to predict and improve outcomes. The process begins when photons excite PS molecules from their ground state to an excited state, initiating reactions that generate reactive oxygen species (ROS) (56). The Jablonski diagram (**Figure 2**) illustrates the quantum transitions underlying this process. In biological systems, these ROS interact with cellular membranes, proteins, and DNA, causing oxidative damage that results in cell death—the therapeutic goal of PDT. Upon absorbing a photon with energy matching the gap between ground state ( $PS_{0}$) and excited singlet state ( $PS_{1}^{*}$), the PS undergoes excitation:

$$PS_{0}+h\nu \to PS_{1}^{*}$$

The excited PS then undergoes intersystem crossing (ISC) to a longer-lived triplet state (^3^ $PS^{*}$) (19). ISC efficiency is critical for PDT effectiveness, as higher ISC yields mean more PS molecules reach the triplet state, leading to more interactions with molecular oxygen and greater ROS production. From the triplet state, the PS can generate cytotoxic species through two competing pathways (19, 57, 58).


$$PS_{1}^{*}\to^{3}P\;S^{*}$$

**Type I: free radical pathway.** The triplet-state PS interacts directly with cellular substrates, transferring electrons or hydrogen atoms to form radicals that react with oxygen to produce superoxide anion ( $O_{2}^{-}$), hydroxyl radical ( OH•), or hydrogen peroxide ( $H_{2}O_{2}$) (59–61).

**Type II: singlet oxygen pathway.** The triplet-state PS transfers energy directly to ground-state molecular oxygen (^3^ $O_{2}$), converting it to highly reactive singlet oxygen (*^1^* $O_{2}$) (59, 60). Singlet oxygen causes extensive oxidative damage to cellular membranes, proteins, and DNA, leading to cell death.


P 3S*+3O2 →PS0+1 O2

Both Type I and Type II mechanisms play crucial roles in PDT, with each pathway offering unique advantages depending on the specific clinical context (57, 58, 61–63). The relative efficiency depends on the PS used and the local tissue microenvironment, including the presence of substrates and the concentration of molecular oxygen. Type I PDT may be more effective in hypoxic (low oxygen) environments for certain tumor microenvironments, as it can still generate ROS through alternative pathways. Type II PDT is typically more efficient in well-oxygenated tissues where singlet oxygen production can occur readily.

### 2.2 Cornerstones of PDT

Three interdependent components majorly determine PDT outcomes: the PS, light parameters, and oxygen availability. PSs are light-activated compounds that generate ROS upon excitation (59, 60, 64). Effective PSs must absorb light in the red to near-infrared region (NIR) (600–800 nm) to achieve adequate tissue penetration. They must also resist photodegradation during prolonged exposure, exhibit high quantum yield for ISC, and accumulate selectively in target tissue. Additionally, they should show minimal dark toxicity to ensure cytotoxic effects occur only upon illumination (65–68). PSs have evolved across several generations. Porphyrins such as Photofrin absorb around 630 nm and offer moderate tissue penetration; they were the first clinically approved agents (64, 69). Chlorins like Chlorin e6 absorb at longer wavelengths (~660 nm), providing improved penetration and higher singlet oxygen quantum yields (64, 70). Phthalocyanines (~670–700 nm) offer excellent photostability, while bacteriochlorins (~735–740 nm) achieve the deepest tissue penetration (71, 72). These agents can be administered topically for superficial lesions, intravenously for deep-seated tumors, or conjugated to antibodies and peptides for targeted delivery. Light characteristics directly influence PDT outcomes (73, 74). The wavelength must match the PS’s absorption spectrum, with red and NIR light preferred for their ability to penetrate several millimeters to centimeters into tissue. For superficial lesions, light is typically delivered externally using lasers or light-emitting diodes, while deep-seated tumors require interstitial optical fibers positioned within the tissue (73, 74). The total energy delivered (fluence, J/cm²) and rate of delivery (irradiance, mW/cm²) both require optimization. Too little energy fails to generate sufficient ROS, while excessive irradiance depletes local oxygen faster than replenishment, paradoxically reducing efficacy. Fractionated protocols address this limitation by interspersing dark intervals between exposures, allowing oxygen replenishment and often improving outcomes over continuous illumination (73). Molecular oxygen is essential for generating the ROS responsible for PDT’s cytotoxic effects, and its availability influences both Type I and Type II reaction efficiency, though Type I shows reduced oxygen dependence (75–77). Because PDT consumes oxygen during ROS generation, a continuous supply is necessary to sustain the photodynamic effect, yet this demand creates a challenge. Prolonged illumination leads to localized oxygen depletion in the treated area, and many solid tumors already exhibit hypoxic regions due to inadequate blood supply (78). Hypoxia particularly impairs Type II PDT, which relies heavily on oxygen for singlet oxygen production. Several strategies have been developed to address this limitation and are reviewed in detail elsewhere (79–81). Understanding and managing the role of molecular oxygen is essential for optimizing PDT protocols and achieving consistent clinical outcomes.

### 2.3 Biological effects of PDT

**Figure 3:**
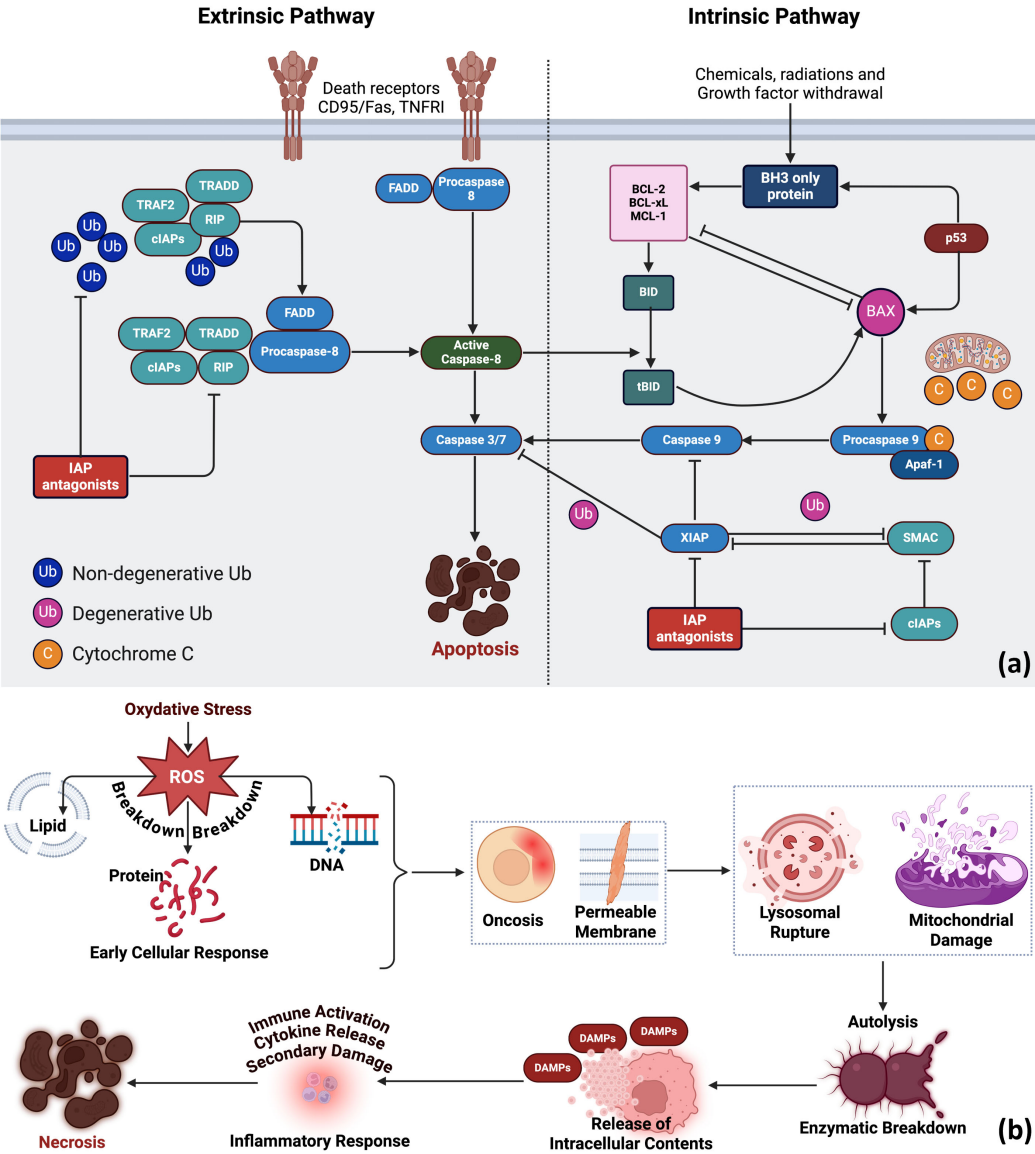
PDT-induced cell death pathways. Upon activation, PS generate ROS that cause oxidative damage to cellular components. **(a)** Moderate damage triggers apoptosis through extrinsic (death receptor) or intrinsic (mitochondrial) pathways, both activating caspase cascades that lead to DNA fragmentation, cell shrinkage, and controlled cell elimination. Created in BioRender. Mallidi, S. (2026) https://BioRender.com/y4wmkdg **(b)** Excessive ROS causes necrosis, an uncontrolled cell death marked by membrane rupture, release of DAMPs, and inflammatory responses. While necrosis can amplify tumor destruction, it may also affect surrounding tissue. Created in BioRender. Mallidi, S. (2026) https://BioRender.com/awd8s04.

PDT destroys tumors through three interconnected mechanisms: direct cell killing, vascular damage, and immune activation (82, 83). The balance among these effects depends on PS localization, light dose, and tissue characteristics. At the cellular level, ROS-mediated damage can trigger either apoptosis or necrosis. When ROS damage is moderate and localized—particularly to mitochondria—cells undergo apoptosis. This controlled form of death is characterized by caspase activation, chromatin condensation, and fragmentation into apoptotic bodies that phagocytes clear without provoking inflammation (**Figure 3a**) (84, 85). The intrinsic pathway, which predominates in PDT-induced apoptosis, begins when mitochondrial damage releases cytochrome C, initiating the caspase cascade. The extrinsic pathway involves ROS-induced upregulation of surface death receptors that activate caspase-8 (86). When oxidative stress is overwhelming, however, cells die by necrosis—an uncontrolled process in which extensive lipid peroxidation destroys membrane integrity, causing cells to swell and rupture (**Figure 3b**) (87–90). Necrotic cells release their contents, including damage-associated molecular patterns (DAMPs), which activate immune cells and trigger inflammation. This inflammatory response can amplify tumor destruction but may also affect surrounding healthy tissue. Beyond direct cytotoxicity, PDT damages tumor vasculature. ROS injure endothelial cells lining tumor blood vessels, disrupting vessel integrity and activating platelets to form microthrombi. The resulting vascular occlusion starves the tumor of oxygen and nutrients, causing widespread ischemic cell death (91, 92). PDT also disrupts angiogenic signaling pathways, inhibiting the formation of new blood vessels and contributing to sustained tumor control. The relative contribution of direct cell killing versus vascular shutdown varies with treatment parameters and can be tuned for specific clinical objectives.

### 2.4 Applications of PDT

The ability to destroy diseased tissue while sparing healthy structures has made PDT valuable across multiple medical specialties. From early applications in esophageal and skin cancers, PDT has expanded to include ophthalmologic disorders, localized infections, gynecologic conditions, and precancerous lesions in multiple organ systems. Its minimally invasive nature and ability to be repeated without cumulative toxicity make it attractive where surgery poses a significant risk or where tissue preservation is paramount. **Table 1** summarizes established and investigational applications.

**Table 1:** Clinical applications of PDT across organ systems.

Application	Description	Reference
Skin cancer	BCC: Effective for superficial and thin nodular (≤2 mm). Actinic keratoses: FDA-approved. Bowen’s disease (SCC *in situ*): Approved internationally. T-cell lymphoma: Targets malignant T-cells in the skin.	(8, 93, 94)
Head & Neck cancer	Oral: Precancerous lesions and early-stage treatment. Laryngeal: Early-stage treatment.	(95, 96)
Lung cancer	Non-Small Cell Lung: Early-stage treatment.	(97)
Gastrointestinal cancer	Esophageal: Treat Barrett’s esophagus with high-grade dysplasia. Gastric: Early-stage treatment. Colorectal: Early-stage treatment and prevention of polyp.	(98–100)
Brain tumor	Glioma: Target residual tumor cells following surgical resection.	(101, 102)
Bladder cancer	Non-muscle invasive bladder cancer: Intravesical PDT for recurrent or BCG-refractory superficial tumors.	(103, 104)
Dermatology	Acne: Target sebaceous glands and reduces bacterial load. Psoriasis: Reduce psoriatic plaques through anti-inflammatory and immunomodulatory effects. Warts: Treat recalcitrant warts by targeting viral-infected keratinocytes.	(105–107)
Ophthalmology	AMD: PDT with verteporfin treats Choroidal Neovascularization. CSC: Reduces subretinal fluid and improves visual acuity.	(108)
Dentistry	Oral Leukoplakia: Targets dysplastic and premalignant cells. Periodontitis: Reduce bacterial load and inflammation.	(109–112)
Rheumatology	Rheumatoid Arthritis: Ex vivo proof-of-concept targeting inflamed synovial tissue.	(113)
Cardiovascular	Atherosclerosis: Phase I trial demonstrated safety and preliminary efficacy in peripheral arterial disease.	(114)
Neurology	Amyloid Plaques: Preclinical research suggests potential to target amyloid aggregates.	(115, 116)
Gynecology	CIN/HPV: High complete response (82%) with HPV clearance; preserves cervical anatomy. VIN: Tissue-sparing treatment for vulvar lesions. VAIN: Effective for vaginal lesions including post-hysterectomy. Early cervical cancer: Fertility preservation for stage IA.	(117–123)
Endometrial cancer	Early-stage endometrial adenocarcinoma: Fertility-sparing treatment for young patients with disease confined to endometrium; documented successful pregnancies.	(124)

AMD, age-related macular degeneration; BCC, basal cell carcinoma; BCG, Bacillus Calmette-Guérin; CSC, central serous chorioretinopathy; SCC, squamous cell carcinoma.

Despite proven efficacy across these diverse applications, PDT outcomes remain inconsistent. Treatment response varies with tumor oxygenation, PS biodistribution, light penetration through irregular tissue geometries, and patient-specific factors that are difficult to assess or predict clinically. This complexity arising from the interplay of photophysical, biological, and anatomical variables creates both a challenge for standardized protocols and an opportunity for computational approaches. The following section introduces machine learning and deep learning techniques that can extract patterns from high-dimensional PDT data and enable the predictive, personalized treatment strategies that conventional methods cannot achieve.

## 3 Learning algorithms: techniques and advances

### 3.1 Machine learning fundamentals

ML encompasses algorithms that identify patterns and make predictions from data without explicit programming for each specific task (26–28). Rather than encoding rules manually, these methods learn relationships directly from examples. This approach is well-suited to the multivariable complexity where interactions among PS properties, light parameters, tissue characteristics, and biological responses resist simple analytical models. The standard workflow partitions data into training sets for model development, validation sets for parameter tuning, and held-out test sets for unbiased performance evaluation. This separation is critical: models that perform well on training data but poorly on new examples have “overfit” to noise rather than learning generalizable patterns. Performance metrics vary by task: accuracy, precision, recall, and F1-score for classification; mean squared error and correlation coefficients for regression (125). Several learning paradigms prove relevant for PDT applications. Supervised learning trains models on labeled data where each input is paired with a known outcome, enabling tasks like predicting treatment response from pretreatment measurements or classifying tissue types from spectroscopic signatures. Unsupervised learning discovers structure in unlabeled data, identifying patient subgroups with similar characteristics or segmenting tissue regions based on optical properties without predefined categories. Reinforcement learning (RL) optimizes sequential decisions through interaction with an environment, receiving feedback that guides future actions. This paradigm is well-suited to adaptive treatment protocols that adjust light delivery based on real-time tissue response. Self-supervised learning, increasingly prominent in modern AI, generates training signals from the data itself, enabling pretraining on large unlabeled datasets before fine-tuning on limited labeled examples. This approach holds particular promise for PDT, where annotated clinical datasets remain scarce, but imaging data accumulates steadily. The choice among these paradigms depends on data availability and clinical goals. Supervised approaches require outcome labels (whether a patient responded, whether tissue was malignant), limiting training to completed cases with documented results. Unsupervised methods can leverage larger imaging datasets without outcome annotation, potentially discovering predictive patterns that supervised approaches constrained by existing labels might miss.

### 3.2 Classical machine learning methods

Since most PDT studies involve only tens to hundreds of labeled cases, algorithm selection must prioritize methods that learn effectively from limited data while providing interpretable predictions. Regression, tree-based, and distance-based approaches meet these criteria. Regression methods model relationships between input variables and continuous outcomes. Linear regression establishes baseline associations between treatment parameters and results, while logistic regression handles binary classification by modeling the probability of outcomes such as treatment success or tumor recurrence (126, 127). These interpretable methods remain valuable not only for prediction but for identifying which input features strongly influence outcomes, providing insight that can guide clinical decision-making and experimental design. Tree-based methods partition data through hierarchical decision rules learned from training examples. Decision trees offer transparency that facilitates clinical adoption: physicians can trace the logic from patient characteristics through branching criteria to predicted outcomes (128). Random forests aggregate predictions from hundreds of trees, each trained on random data subsets, improving accuracy while reducing the overfitting that plagues individual trees (31). Gradient boosting methods such as XGBoost and LightGBM build trees sequentially, with each new tree correcting errors from previous ones (129). These ensemble approaches frequently achieve state-of-the-art performance on tabular clinical data and provide built-in metrics for feature importance. SVMs find optimal decision boundaries by maximizing the margin between classes (130). Kernel functions project data into higher-dimensional spaces where linear separation becomes possible, enabling SVMs to capture complex nonlinear relationships in tissue classification tasks. KNN takes a simpler approach, classifying new samples based on similarity to labeled examples in feature space (131). While computationally straightforward, KNN can capture local patterns useful for patient matching applications. Clustering algorithms discover natural groupings without predefined labels (132). K-means partitions data into clusters based on distance to learned centroids, while hierarchical clustering builds tree-structured relationships revealing multi-scale organization. Both approaches help identify patient phenotypes or tissue subtypes that may respond differently to treatment, enabling stratified protocols tailored to subgroup characteristics. For the small-to-moderate datasets characteristic of single-institution PDT studies (typically tens to hundreds of patients), these classical methods often outperform DL approaches that require orders of magnitude more training examples. Their interpretability also addresses a practical concern: clinicians understandably hesitate to act on predictions from opaque models they cannot interrogate or explain to patients.

### 3.3 Deep learning architectures

**Figure 4:**
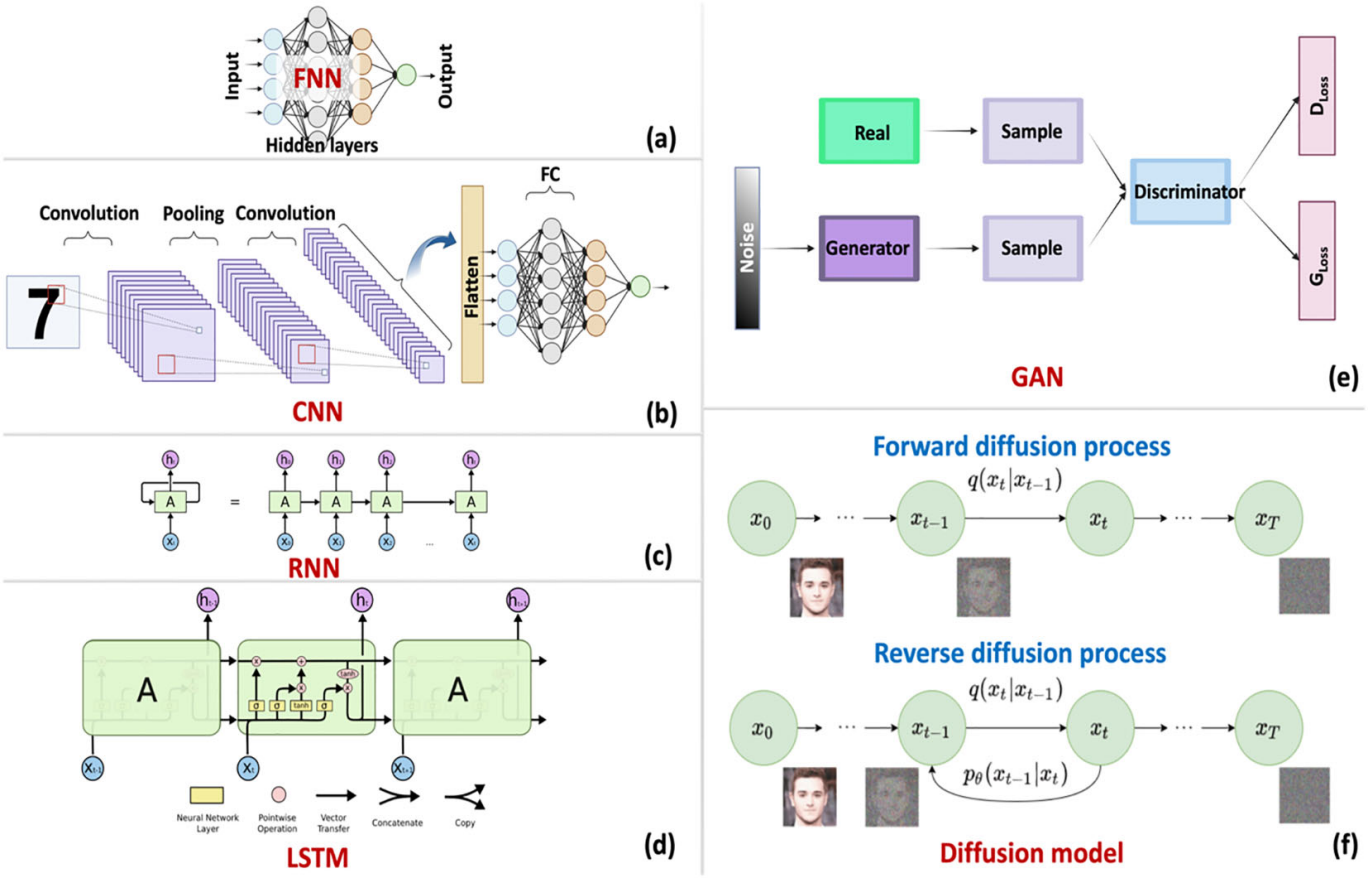
Deep learning architectures relevant to PDT applications. **(a)** Feedforward neural network (FNN) with input, hidden, and output layers. **(b)** Convolutional neural network (CNN) showing convolution, pooling, and fully connected layers for image processing. **(c)** Recurrent neural network (RNN) with temporal unfolding. **(d)** Long short-term memory (LSTM) unit showing gating mechanisms that control information flow. **(e)** Generative adversarial network (GAN) architecture with generator and discriminator components. **(f)** Diffusion model illustrating forward noise addition and reverse denoising processes. LSTM diagram adapted from Olah (https://colah.github.io/posts/2015-08-Understanding-LSTMs/); diffusion model adapted from (144).

DL extends ML through multi-layer neural networks that automatically discover hierarchical feature representations from raw data (133–137). This eliminates the manual feature engineering required by classical methods, a substantial advantage when relevant features are unknown or difficult to specify mathematically. The tradeoff is data hunger: deep networks typically require thousands to millions of training examples to learn robust representations without overfitting. The source of this representational power lies in the network architecture itself. Neural networks consist of interconnected computational units organized in layers. Each unit applies learned weights to its inputs, sums the weighted values, and passes the result through a nonlinear activation function such as rectified linear unit or sigmoid (138). Training adjusts weights throughout the network via backpropagation, an algorithm that efficiently computes how each weight contributes to prediction errors and updates them accordingly (139). Stacking many layers enables the network to learn progressively abstract representations, from simple patterns in early layers to complex concepts in deeper layers (**Figure 4**). Convolutional neural networks (CNNs) revolutionized image analysis by learning spatial filters that detect meaningful patterns regardless of their location in an image (140). Early convolutional layers learn to detect edges and textures; deeper layers combine these into higher-level features like tumor margins or necrotic regions. Pooling operations between layers reduce spatial dimensions while preserving important information, making CNNs robust to small shifts in feature position. These properties make CNNs particularly valuable for PDT imaging applications: segmenting tumor boundaries from fluorescence images, classifying tissue types from OCT scans, or detecting treatment response from serial imaging. Recurrent neural networks (RNNs) and their long short-term memory (LSTM) variants process sequential data by maintaining hidden states that carry information across time steps (141). Standard RNNs struggle with long sequences because gradients vanish or explode during backpropagation through many time steps. LSTMs address this limitation through gating mechanisms that control information flow, deciding what to remember, what to forget, and what to output at each step (142). These architectures suit PDT applications involving temporal dynamics: monitoring photobleaching kinetics, tracking oxygenation changes during treatment, or analyzing time-series spectroscopic measurements. Generative models learn to create new data samples resembling their training distribution. Generative adversarial networks (GANs) pit two networks against each other: a generator that creates synthetic samples and a discriminator that distinguishes real from generated data (143). Through adversarial training, generators learn to produce increasingly realistic outputs. Diffusion models take a different approach, learning to reverse a gradual noise-addition process to generate samples from pure noise (144). Both architectures address PDT’s data scarcity challenge by generating synthetic training examples and augmenting limited clinical datasets with plausible variations. Transformers and attention mechanisms, originally developed for natural language processing, increasingly impact biomedical imaging and multimodal data integration (41). Self-attention enables each element in a sequence to attend to all other elements, capturing long-range dependencies without the sequential processing constraints of RNNs. Vision transformers apply these principles to images by treating image patches as sequence elements. For PDT, transformers offer potential for integrating diverse data types, combining imaging, spectroscopy, and clinical variables in unified models that learn cross-modal relationships.

With these algorithmic foundations established, attention turns to their practical deployment. The diversity of methods reflects the diversity of PDT challenges: image segmentation favors CNNs, temporal monitoring suits LSTMs, molecular design leverages generative models, and emerging multimodal problems may benefit from transformer architectures. Section 4 examines specific applications across the PDT pipeline. **Table 2** summarizes representative ML/DL methods applied across five key domains of PDT: tissue optical characterization, PS design, nanoparticle optimization, treatment monitoring, and outcome prediction.

**Table 2:** Overview of AI (ML/DL) approaches in PDT research.

Application area	AI (ML/DL) method	Key task	Performance/outcome	Validation	Reference
1. Tissue optical characterization					
*Fluorophore quantification*	LS-SVM	Quantify fluorophore concentration, classify tissue	<5% quantification error for PpIX (400–1000 nM); 100% diagnostic accuracy	Phantom + Clinical	(146)
*Optical property recovery*	ANN, GBDT	Real-time $\mu_{a}$, $\mu_{s}^{'}$ extraction for iPDT	<6% average error in OP recovery; enabled dynamic dosimetry with PDT-SPACE integration	*In silico*	(145)
*Physics-guided prediction*	Physics-guided NN	Tissue optical properties estimation	Improved accuracy and out-of-domain generalizability	*In silico*	(147)
*Transfer learning for probes*	ANN + Transfer Learning	Adapt models across fiber probes with varying SDS	Reduced prediction errors for MB concentration (RMSE improved from 1.67 to 0.38 μM)	Phantom	(148)
2. Photosensitizer design					
Φ_Δ_ *prediction*	XGBoost, AdaBoost	Predict singlet oxygen quantum yield	R² = 0.86 (XGBoost/MFPs); experimentally validated; SHAP interpretability	*In silico* + *In vitro*	(152)
*BODIPY screening*	QSPR/MLR	Predict Φ_Δ_ across solvents for heavy-atom-free BODIPY	R = 0.88–0.91; q² = 0.62–0.69; RMSE of 8.2% (toluene)	*In silico* + *In vitro*	(149)
*TMC photosensitizers*	DFT-ML hybrid (SVR, KRR, MoE)	Predict Φ_Δ_ for Ru/Ir/Re metal complexes	R² > 0.9 (test); MoE R² = 0.87 (external); physics-informed	*In silico*	(153)
*Wavelength prediction*	GCN	Predict $\lambda_{max}$, emission, PLQY, lifetime	RMSE = 26.6 nm (absorption), 28.0 nm (emission); 30,094 samples	*In silico*	(155)
*Multi-objective PS design*	Graph Transformer + Bayesian (MoLeR/SolutionNet)	*De novo* PS design optimizing Φ_Δ_ and $\lambda_{max}$	Validated HB4Ph synthesis (Φ_Δ_ = 0.85, $\lambda_{max}\;$= 645 nm); 6,148 candidates	*In silico* + *In vitro*	(157)
*Type I PS identification*	1-PS-GCN (fingerprint-enhanced GCN)	Classify Type I mechanism for NIR RNA-targeted PDT	Accuracy 0.88; 782 Type I PS from 2,768; PYD experimentally validated	*In silico* + *In vitro* + *In vivo* (mouse model)	(162)
*Phototoxicity prediction*	RF, DNN + TD-DFT	Predict 3T3 NRU phototoxicity	83–85% accuracy; 86–90% sensitivity; phototoxophore identification	*In silico*	(164)
*Phototoxicity (GCN)*	GCN (kMoL) + HOMO-LUMO gap	Predict *in vitro* phototoxicity	F1 score of 0.857; interpretable via IG method	*In silico*	(165)
3. Nanoparticle design					
*Protein corona prediction*	Random Forest ensemble + meta-analysis	Predict corona composition and cellular recognition	R² > 0.80 (apolipoproteins, immune proteins); validated on 4 NP types	*In vitro*	(168)
*Protein abundance*	ERT, RF, GBDT	Predict relative protein abundance	F1 score of 0.893 (ERT); R² = 0.534 (RF regression); 178 proteins; SHAP interpretability	*In silico* (meta-analysis)	(169)
*Cellular uptake*	ANN (feedforward + backprop)	Predict NP internalization and cancer staging	Q² = 0.9 (internalization); >98% accuracy (cancer classification); TNBC differentiation	*In vitro*	(170)
*PBPK modeling*	AI-assisted PBPK (DNN + RF)	Predict tumor delivery efficiency	R² = 0.82 (DEmax); 378 tumor datasets; non-animal alternative	*In silico* (meta-analysis)	(171)
*Lipid NP generation*	ML	Screen ionizable lipids for mRNA-LNP delivery	584 lipids synthesized via 4-component reaction; 40,000 virtual library screened; Lipid 119–23 outperformed MC3 and ALC-0315	*In silico* + *In vivo* (mouse model)	(172)
4. Treatment monitoring					
*Vessel segmentation*	GA-Xnet (Attention U-Net + UNet++)	Subfascial vessel segmentation for V-PDT	Accuracy = 96%, Sensitivity = 86%, Specificity = 96%; preserved vessel skeleton connectivity	*In vivo* (animal)	(173)
*PDT/PTT optimization*	Regression, Interpolation, AFF	Optimize laser intensity/duration for PDT/PTT	Identified optimal parameters for combined therapy; death rate errors of 0.09–0.15	*In vitro*	(174)
*Cell response analysis*	Cellpose (U-Net-based CNN)	Cancer cell segmentation post-PDT	Morphological characterization of 16,100 cells; ~86% segmentation accuracy	*In vitro*	(175)
5. Outcome prediction					
*CSC treatment response*	ResNet50 + XGBoost (DeepPDT-Net)	Predict 1-year PDT outcomes for chronic CSC	88.0% accuracy; external validation AUC 0.917	Clinical (retrospective)	(176)
*Diabetic wound healing*	PCA + Raman spectroscopy	Quantify LDPDT wound effects	Biomarker identification via Mahalanobis distance; 4 J/cm² dose most effective	*In vivo* (mouse model)	(177)
*Choroidal analysis*	U-Net denoising + 3D segmentation	Volumetric choroid analysis after PDT/PC	3D treatment response insights; significant vessel/stromal volume reduction (P = 0.00029, P = 0.0014)	Clinical (retrospective)	(178)
*OLK recurrence*	Autoencoder + Cox regression	Predict recurrence after ALA-PDT	AUC 0.872 for complete response; recurrence AUC improved from 0.564 to 0.882 with DL features	Clinical (retrospective)	(179)
*OCT-based prediction*	DenseNet-based CNN	Predict PDT response in chronic CSC	Accuracy 0.529–0.670; precision 0.57–0.74 depending on comparison groups	Clinical (retrospective)	(180)

Methods: ANN, Artificial Neural Network; AFF, Analytical Function Fitting; CNN, Convolutional Neural Network; DFT, Density Functional Theory; DNN, Deep Neural Network; ERT, Extremely Randomized Trees; GANs, Generative Adversarial Networks; GA-Xnet, Global Attention-Xnet; GBDT, Gradient Boosted Decision Trees; GCN, Graph Convolutional Network; GNNs, Graph Neural Networks; KRR, Kernel Ridge Regression; LS-SVM, Least Squares Support Vector Machine; MLR, Multiple Linear Regression; MoE, Mixture of Experts; PBPK, Physiologically Based Pharmacokinetic; PCA, Principal Component Analysis; QSPR, Quantitative Structure-Property Relationship; RF, Random Forest; SVR, Support Vector Regression; TD-DFT, Time-Dependent DFT; XGBoost, Extreme Gradient Boosting.

Metrics: AUC, Area Under the Curve; PLQY, Photoluminescence Quantum Yield; R², Coefficient of Determination; RMSE, Root Mean Square Error.

Domain: ALA, 5-Aminolevulinic Acid; BODIPY, Boron-Dipyrromethene; CSC, Central Serous Chorioretinopathy; iPDT, Interstitial PDT; LDPDT, Low-Dose PDT; LNP, Lipid Nanoparticle; NP, Nanoparticle; OCT, Optical Coherence Tomography; OLK, Oral Leukoplakia; OP, Optical Properties; PDT, Photodynamic Therapy; PpIX, Protoporphyrin IX; PS, Photosensitizer; PTT, Photothermal Therapy; SDS, Source-Detector Separation; TMC, Transition Metal Complex; TNBC, Triple-Negative Breast Cancer; V-PDT, Vascular PDT; Φ_Δ_, Singlet Oxygen Quantum Yield; $\lambda_{max}$, Maximum Absorption Wavelength.

## 4 Applications of learning algorithms in PDT

Before examining specific applications, it is important to contextualize the current state of AI-PDT research. The studies reviewed here span a spectrum of validation stages, from computational simulations and phantom experiments through preclinical animal models to retrospective analyses of clinical data. Notably, no AI-PDT application has yet achieved prospective clinical validation or regulatory approval. **Table 2** categorizes each study by validation stage to help readers distinguish between proof-of-concept demonstrations and approaches closer to clinical translation. This distinction is critical for assessing the maturity and near-term clinical relevance of different AI applications in PDT.

### 4.1 Tissue optical characterization and light dosimetry

**Figure 5:**
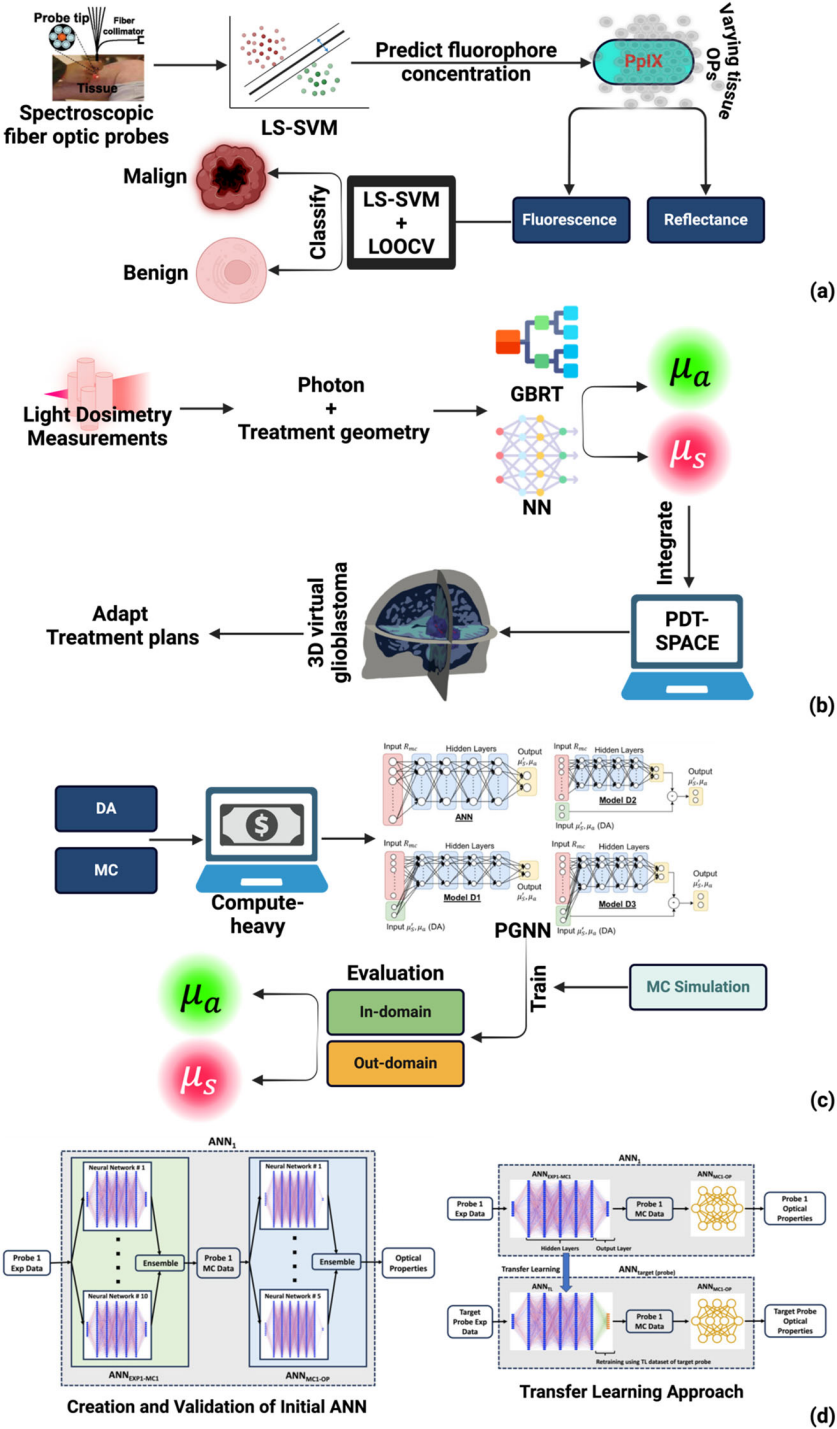
Machine learning approaches for tissue optical characterization and light dosimetry. **(a)** LS-SVM regression predicts fluorophore concentration from spectroscopic fiber optic probe measurements while compensating for varying tissue optical properties; combined with leave-one-out cross-validation (LOOCV), the model classifies tissue as malignant or benign. Probe image adapted from (146). **(b)** Neural networks and gradient boosting regression trees (GBRT) recover absorption ( $\mu_{a}$) and scattering ( $\mu_{s}$) coefficients from light dosimetry measurements; integration with PDT-SPACE enables adaptive treatment planning validated on 3D virtual glioblastoma models. Adapted from (145). **(c)** Physics-guided neural networks (PGNN) incorporate diffusion approximation (DA) inputs to overcome computational limitations of Monte Carlo (MC) simulations; in-domain and out-domain evaluation demonstrates improved generalizability. Adapted from (147). **(d)** Transfer learning adapts an ANN ensemble trained on one probe configuration to new probes with different source-detector separations, reducing calibration requirements for multi-site deployment. Adapted from (148).

Light propagation through tissue depends on absorption and scattering coefficients that vary between patients, tumor types, and even within individual lesions. Traditional PDT protocols assumed nominal optical properties, but these patient-specific variations significantly affect light dosimetry and treatment outcomes (145). ML offers approaches to recover optical properties in real time and adjust light delivery to individual tissue characteristics, with methods maturing over the past decade from proof-of-concept toward clinical deployment. Among the first applications of ML to PDT instrumentation, Xie et al. demonstrated spectroscopic probes enhanced by SVM regression for intraoperative guidance (146). Fiber optic systems combining fluorescence and reflectance measurements generate complex signals influenced by PS concentration, tissue optical properties, and ambient light contamination. Strong ambient light in the operating room poses particular challenges for accurate measurements. The team addressed this through a hand-held fiber optic probe system with fast pulse modulation and frequency-specific detection to suppress background interference. Their nonlinear least-squares SVM (LS-SVM) regression model quantified protoporphyrin IX concentrations in tissue phantoms while compensating for large variations in tissue scattering and absorption. This approach ensured that fluorescence signals correlated linearly with true fluorophore concentrations despite optical property variations. Applied to clinical skin lesion data, the approach achieved 100% diagnostic precision for tissue classification (**Figure 5a**). This work established that classical ML methods could handle the nonlinear signal dependencies inherent in real surgical settings, but the approach remained limited to pre-treatment tissue characterization with site-specific probe configurations. The question of whether ML could enable adaptation during treatment, rather than just before it, was addressed by Yassine et al. in their work on interstitial PDT for deep-seated tumors (145). When light must propagate through centimeters of tissue, uncertainties in optical properties become critical since small errors compound over longer path lengths. The team employed cylindrical diffusers that serve as both light sources and detectors, using photon measurements combined with treatment geometry and source locations as input features for neural networks and gradient boosting regression trees (GBRT). These models predicted the absorption coefficient ( $\mu_{a}$) and reduced scattering coefficient (µ_s_′) of tumor tissues in real time. The key advance was integration with treatment planning software PDT-SPACE, enabling immediate adjustment of optical power allocations based on recovered properties rather than assumed nominal values (**Figure 5b**). Validation on 3D virtual glioblastoma models constructed from real MRI images demonstrated significantly reduced prediction errors in critical volumes compared to conventional planning. This represented a shift from static pre-treatment characterization toward dynamic intra-treatment adaptation. However, purely data-driven neural networks, while fast and flexible, can operate as black boxes that occasionally produce physically unrealistic outputs such as negative optical property values.

Chong and Pramanik addressed this limitation through physics-guided neural networks (PGNN) that embed domain knowledge directly into network architecture (147). Traditional methods for estimating optical properties relied on physics-based models like the diffusion approximation (DA) or MC simulations. These approaches simulate light transport accurately but are computationally intensive, limiting their utility for real-time applications. Pure data-driven networks offer speed but sacrifice interpretability and physical consistency. The physics-guided approach bridges this gap by incorporating DA results as network inputs and applying physics-based constraints during training (**Figure 5c**). Evaluation on simulated datasets of single-layer tissue samples, with separate in-domain and out-domain test sets, demonstrated that these hybrid networks outperformed both traditional ML models and unconstrained neural networks in prediction accuracy, generalizability, and consistency with physical laws. By ensuring predictions remain physically plausible, this approach builds the reliability necessary for clinical trust. Yet even robust, physics-informed models face a practical barrier to adoption: spectroscopic probes vary in geometry and optical characteristics across clinical sites, and models trained on one configuration may not transfer directly to another. Transfer learning offers a solution to this deployment challenge. Hannan and Baran developed an artificial neural network (ANN) to predict optical properties from diffuse reflectance spectra collected by a specific fiber optic probe. The network used 8 input nodes corresponding to different source-detector separations and 2 output nodes for absorption and reduced scattering coefficients (148). The network combined two ensembles, one mapping experimental spectra to simulated reflectance and another mapping simulated reflectance to optical properties (**Figure 5d**). To adapt this model to probes with different source-detector configurations, they applied transfer learning by freezing the feature-extraction layers and retraining only output layers with small datasets from target probes. This approach significantly reduced prediction errors compared to applying the original model directly, as measured by root mean squared error and mean absolute percent error. The practical implication is substantial: rather than building and validating new models for each clinical site, institutions can adapt proven models to their equipment with minimal additional data collection. Collectively, these studies trace maturation from demonstrating feasibility to enabling practical deployment. Early work proved ML could handle clinical signal complexity. Subsequent advances enabled real-time treatment adaptation, physics-informed predictions that clinicians can trust, and transfer learning that reduces barriers to multi-site adoption. To summarize the translational status of these approaches: Xie et al. represents the only study with validation on human patients, achieving 100% diagnostic accuracy for skin tumor classification during clinical PDT. Yassine et al. and Chong and Pramanik developed their methods using computational simulations, while Hannan and Baran validated their methods using tissue-mimicking phantoms. This distribution, predominantly simulation and phantom studies with limited clinical validation, reflects the broader landscape of AI in PDT dosimetry and underscores the need for prospective clinical trials before implementation.

### 4.2 AI-driven photosensitizer development and design

**Figure 6:**
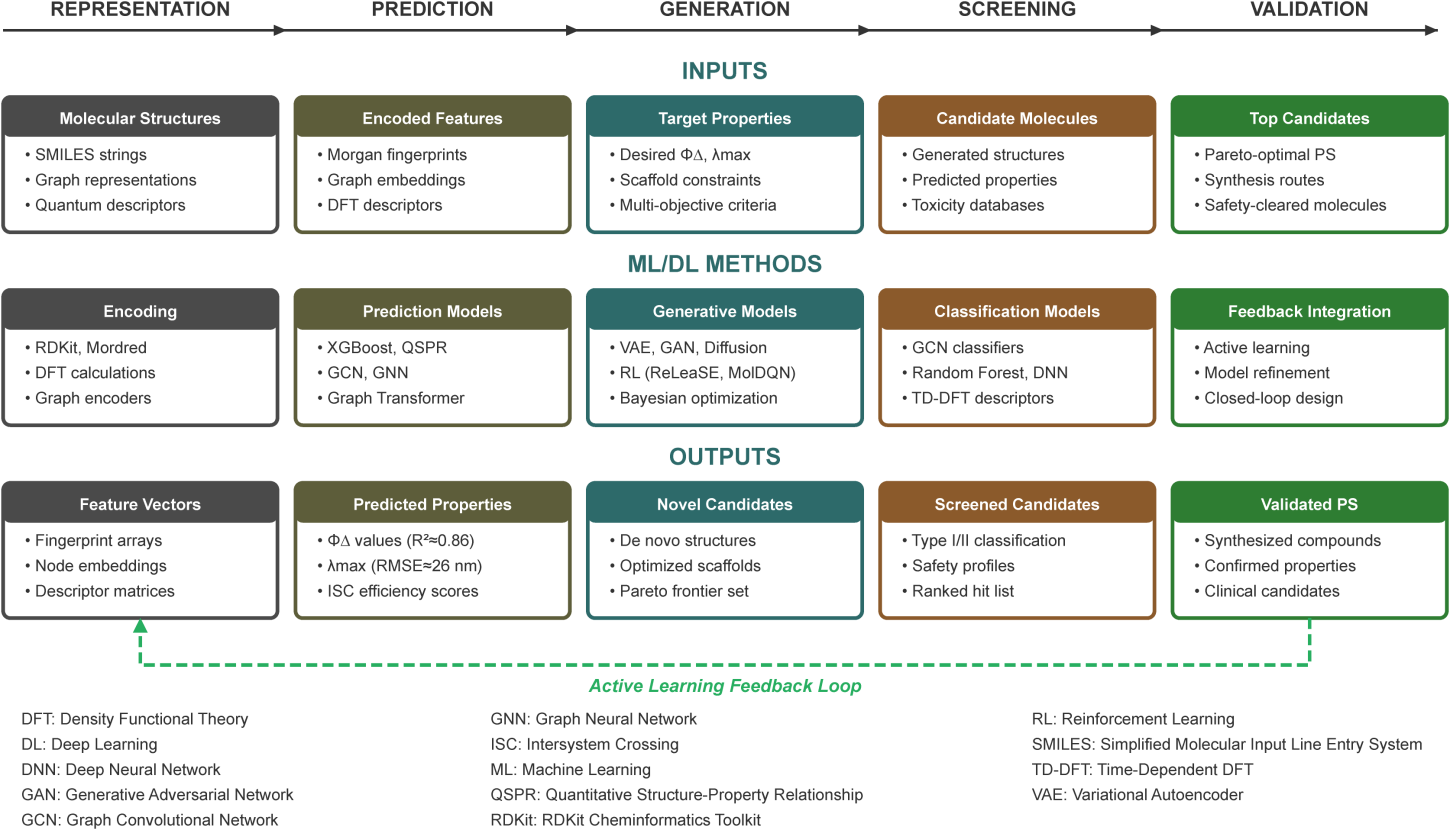
AI-driven photosensitizer development pipeline. The workflow progresses through five integrated stages. Inputs (top row): molecular structures are encoded as SMILES, fingerprints, or graphs; encoded features feed property prediction; target properties guide generative design; candidate molecules undergo screening; top candidates proceed to validation. AI/ML Methods (middle row): encoding tools (RDKit, Mordred, DFT), prediction models (XGBoost, GCN, Graph Transformer), generative models (VAE, GAN, RL), classification models (GCN, Random Forest, DNN), and feedback integration through active learning. Outputs (bottom row): feature vectors, predicted properties (Φ_Δ_, $\lambda_{max}$), novel candidates, screened candidates, and validated PSs.

The discovery and optimization of PSs represent one of the most promising applications of artificial intelligence in PDT. Traditional PS development has relied on iterative synthesis and characterization, a process that is time-consuming, resource-intensive, and often guided by chemical intuition rather than systematic exploration of the vast molecular space (46). The integration of ML and DL approaches into PS design offers opportunities to accelerate this process while achieving optimized photophysical and biological properties tailored for therapeutic applications. Advances have progressed from predicting properties of known compounds to generating novel structures and screening for clinical viability. **Figure 6** illustrates this integrated pipeline, showing how molecular representations feed into property prediction, generative design, mechanism screening, and experimental validation through an active learning feedback loop.

#### 4.2.1 Predictive modeling of photosensitizer properties

A fundamental challenge in PS development is accurately predicting photophysical properties that determine PDT efficacy, particularly the singlet oxygen quantum yield (Φ_Δ_), absorption wavelength, and ISC efficiency. These properties are critical for Type II PDT, where singlet oxygen generation directly correlates with therapeutic outcomes (56). Traditional quantum chemical calculations, such as time-dependent density functional theory (TD-DFT), while accurate, are computationally expensive and impractical for high-throughput screening of large molecular libraries. Quantitative structure-property relationship (QSPR) models emerged as early tools for predicting PS photophysical properties. Foundational work established approaches using linear regression and SVMs to correlate molecular descriptors with singlet oxygen quantum yields for porphyrins and BODIPYs (149, 150). These models demonstrated that PS efficacy is influenced by multiple molecular descriptors, including lipophilicity, electrostatic parameters, and quantum chemical descriptors such as HOMO-LUMO gaps (151). While limited in accuracy, this work established that computational prediction of PS properties was feasible. Recent advances have substantially improved prediction accuracy through more sophisticated ML approaches. He et al. developed ML models using Morgan fingerprints and combined molecular/quantum chemical descriptors to predict singlet oxygen quantum yields across diverse PS structures (152). Their XGBoost model achieved R^2^ values of 0.86, demonstrating superior performance compared to traditional approaches. Importantly, these models incorporated experimental conditions, including solvent type and excitation wavelength, that significantly influence measured Φ_Δ_ values. This approach provided more universally applicable predictions rather than predictions tied to specific measurement conditions. Specialized approaches address particular PS classes. For heavy-atom-free BODIPY PSs that undergo spin-orbit charge transfer ISC (SOCT-ISC), Buglak et al. established QSPR models using over 5,000 calculated molecular descriptors (149). Their multiple linear regression models achieved correlation coefficients of R = 0.88–0.91 for predicting Φ_Δ_ values across solvents of varying polarity, enabling pre-synthetic screening of environment-activatable PS candidates. This approach is particularly valuable for designing theranostic agents that combine fluorescence imaging with PDT capabilities, where balancing fluorescence quantum yield and singlet oxygen generation presents a fundamental design challenge. For transition metal complex PSs, Wang et al. proposed a hybrid DFT-ML framework where excited-state quantum chemistry descriptors were integrated with molecular descriptors to predict Φ_Δ_ for Ru-, Ir-, and Re-based complexes (153). This physics-informed approach addresses the limitation that traditional SMILES-based representations cannot adequately capture the structural complexity of metal-ligand interactions.

Graph neural networks (GNNs) have emerged as particularly powerful architectures for molecular property prediction due to their ability to naturally represent molecules as graphs where atoms correspond to nodes and bonds to edges (154). Unlike descriptor-based methods requiring manual feature engineering, GNNs automatically learn hierarchical molecular representations capturing both local and global structural features. Joung et al. employed graph convolutional networks trained on over 30,000 chromophore-solvent combinations to predict peak absorption and emission wavelengths (155). Their approach accounts for chromophore-solvent interactions through concatenated feature vectors, achieving a root mean square error of 26.6 nm for absorption wavelength prediction across diverse molecular scaffolds. The integration of molecular fingerprints with graph representations has further enhanced prediction capabilities. Hybrid GNN architectures combining molecular graphs with Morgan fingerprints capture both topological and electronic features, achieving R² values up to 0.92 for maximum absorption wavelength prediction (156). These advances are particularly relevant for PS design, where absorption in the red to NIR is essential for therapeutic tissue penetration. Graph transformers incorporating attention mechanisms represent the current frontier. The AAPSI (AI-Accelerated PhotoSensitizer Innovation) workflow employs graph transformers trained on a curated database of over 102,000 PS-solvent pairs to predict both Φ_Δ_ and $\lambda_{max}$ simultaneously (157). This multi-objective approach enables identification of PS candidates that optimize the inherent trade-off between singlet oxygen generation efficiency and absorption wavelength, a trade-off that single-property optimization cannot address.

#### 4.2.2 Generative models for *de novo* PS design

Property prediction enables screening of existing compounds, but generative AI models offer the more revolutionary capability to design novel PS structures with desired properties from scratch. This represents a paradigm shift from traditional approaches that rely on modifying known scaffolds based on chemical intuition. Deep generative models, including variational autoencoders (VAEs), GANs, and transformer-based architectures, can learn the underlying distribution of molecular structures and generate novel candidates satisfying multiple property constraints (158). RL approaches enable goal-directed molecular optimization where generated structures are iteratively refined based on predicted property scores. The ReLeaSE (Reinforcement Learning for Structural Evolution) framework combines generative and predictive neural networks, training them jointly to bias molecular generation toward desired properties (159). For PS optimization, RL agents can maximize singlet oxygen quantum yield while simultaneously optimizing for red-shifted absorption, low dark toxicity, and synthetic accessibility. Multi-objective RL frameworks such as MolDQN (Molecule Deep Q-Networks) directly define modifications on molecules while ensuring 100% chemical validity, avoiding the issue of generating synthetically infeasible structures (160). Diffusion models, which learn to reverse a noise-adding process, represent an emerging class of generative architectures showing promising results for molecular design (161). Their application to PS design, while nascent, holds potential for generating structurally novel candidates beyond the chemical space of known PSs. The first experimentally validated AI-driven PS design workflow was demonstrated through the AAPSI platform (157). This closed-loop framework integrates expert knowledge, scaffold-based molecule generation, and Bayesian optimization. From 23 expert-curated scaffolds, the system generated 6,148 synthetically accessible candidates screened using graph transformers. The hypocrellin-based candidate HB4Ph, identified at the Pareto frontier of high Φ_Δ_ and long $\lambda_{max}$, was subsequently synthesized and experimentally validated, demonstrating exceptional photodynamic performance. This validation establishes that AI-generated PS candidates can exhibit competitive or superior performance compared to empirically discovered PSs, moving the field from theoretical promise to demonstrated capability.

#### 4.2.3 Mechanism and safety screening

Beyond photophysical efficacy, clinical viability requires control over photochemical mechanism and safety profile. The distinction between Type I and Type II PDT mechanisms has significant implications for treating hypoxic tumors, where Type I PSs may offer advantages due to reduced oxygen dependence (61). AI models capable of classifying PS mechanism preference are therefore clinically valuable. Chen et al. developed a multi-stage screening workflow combining a graph convolutional network for Type I PS identification with subsequent screening for NIR absorption and RNA-targeting capabilities (162). From an initial pool of 2,768 molecules, the model identified 782 Type I PSs, demonstrating the ability to navigate chemical space toward mechanistically distinct classes. Chemical space visualization using t-SNE dimensionality reduction revealed that Type I and non-Type I PSs exhibit distinct structural distributions, reflecting underlying electronic features governing photochemical mechanism. For Type II PSs, models targeting the singlet-triplet energy gap and ISC efficiency are critical. Chen et al. developed fragment-based and character-based models integrating conditional transformers, recurrent neural networks, and RL (163). These models significantly outperformed traditional baselines with prediction accuracies of 73% versus 4% for identifying high-efficiency triplet PSs. An ideal PS should exhibit minimal dark toxicity while maximizing phototoxicity upon illumination. Predicting these properties is essential for identifying clinically viable candidates during early development. ML models for phototoxicity prediction have leveraged data from standardized assays such as the 3T3 Neutral Red Uptake (NRU) test. Schmidt et al. developed Random Forest and deep neural network models using pharmacophoric fingerprints and quantum chemical descriptors, achieving accuracies of 83–85% with sensitivity reaching 86–90% (164). Graph convolutional approaches have further improved prediction; Igarashi et al. developed a model achieving F1 scores of 0.857 for predicting phototoxicity (165). Importantly, integrated gradient methods enable visualization of substructures contributing to predictions, providing interpretable insights for structural optimization rather than black-box classification. Multi-task deep learning models trained on *in vitro*, *in vivo*, and clinical toxicity data offer comprehensive screening by simultaneously predicting multiple toxicity endpoints (166). The integration of mechanism classification and safety prediction into virtual screening workflows enables comprehensive candidate evaluation before synthesis. Combined with active learning strategies that iteratively select the most informative compounds for experimental evaluation, these approaches can accelerate PS discovery while minimizing experimental costs (167). The AAPSI validation of HB4Ph demonstrates that this integrated pipeline, from property prediction through generative design to mechanism and safety screening, can yield experimentally validated PSs with superior performance (157).

### 4.3 AI in nanoparticle design and drug delivery systems

Nanoparticle-based delivery systems have emerged as essential platforms for enhancing PDT efficacy by addressing fundamental limitations of conventional PS administration. Traditional PSs often suffer from poor water solubility, nonspecific biodistribution, suboptimal tumor accumulation, and inadequate tissue penetration depth (181, 182). Nanocarriers, including liposomes, polymeric nanoparticles, mesoporous silica, metal-organic frameworks, and upconversion nanoparticles, offer solutions to these limitations. They provide enhanced permeability and retention effects, active targeting capabilities, and the ability to co-deliver multiple therapeutic agents (183, 184). However, the vast parameter space governing nanoparticle design, encompassing size, shape, surface chemistry, composition, and drug loading, has traditionally required extensive trial-and-error experimentation. The integration of AI and ML into nanoparticle design enables rational, data-driven optimization that accelerates development timelines while improving therapeutic outcomes (185, 186).

#### 4.3.1 Machine learning for nanoparticle synthesis optimization

The synthesis of nanoparticles for PDT applications involves numerous interdependent variables that collectively determine physicochemical properties and biological performance. ML algorithms have demonstrated remarkable capability in navigating this complex parameter space to optimize synthesis conditions and predict nanoparticle characteristics. Tao et al. provided a comprehensive framework demonstrating how ML can guide the synthesis of colloidal nanocrystals by learning structure-property relationships from experimental data, significantly reducing the number of experiments required to achieve target specifications (187). For lipid-based nanoparticles, which serve as important carriers for PS delivery, AI-driven approaches have revolutionized formulation development. Li et al. developed an accelerated ionizable lipid discovery platform that combines ML with combinatorial chemistry (172). By screening 584 ionizable lipids and training models on the resulting data, they predicted transfection efficiency across a virtual library of 40,000 compounds. The identified candidates outperformed established benchmarks in cellular delivery, demonstrating the power of computational screening to identify optimal formulations without exhaustive experimentation. Similarly, the AI-Guided Ionizable Lipid Engineering platform extends this approach by employing GNNs to screen thousands of lipid variants, achieving enhanced delivery efficiency while dramatically reducing experimental burden (188). ANNs and gradient boosting algorithms have proven particularly effective for predicting nanoparticle size, polydispersity, and encapsulation efficiency based on synthesis parameters. Chaurawal et al. demonstrated the integration of ML with Design of Experiments approaches to optimize sorafenib-loaded nanoparticles, achieving precise control over particle characteristics while minimizing experimental iterations (189). The combination of automated optimization algorithms with high-throughput synthesis platforms enables rapid exploration of formulation space that would be impractical through conventional approaches. Physics-informed ML models represent an emerging strategy incorporating domain knowledge into predictive frameworks. By constraining model predictions to obey fundamental physical principles governing nanoparticle formation and behavior, these hybrid approaches achieve improved generalizability and interpretability compared to purely data-driven methods (190). Such models can predict not only synthesis outcomes but also the relationship between synthesis conditions and downstream biological performance, enabling end-to-end optimization of nanoparticle formulations.

#### 4.3.2 Predicting nano-bio interactions and biodistribution

Understanding how nanoparticles interact with biological systems is critical for designing effective PDT delivery platforms. Upon introduction into biological fluids, nanoparticles rapidly acquire a protein corona that fundamentally alters their biological identity, affecting biodistribution, cellular uptake, and therapeutic efficacy (191). Predicting protein corona composition and its functional consequences has traditionally been challenging due to the complex interplay of physicochemical factors governing protein adsorption. ML approaches have demonstrated significant progress in addressing this challenge. Ban et al. developed ML models that predict the functional composition of the protein corona and subsequent cellular recognition of nanoparticles, achieving R^2^ values exceeding 0.75 for functional protein categories including immune proteins, complement proteins, and apolipoproteins (168). Their analysis identified nanoparticle surface modification and core material as the most important factors determining corona composition, providing actionable insights for rational nanocarrier design. Fu et al. extended this work by employing ensemble methods, including Extremely Randomized Trees and GBRT, to predict relative protein abundance on protein coronas, enabling comprehensive prediction of multiple proteins simultaneously (169). The Protein Corona Database represents a significant resource for the field, compiling data from over 80 studies spanning 2000–2024 and integrating quantitative profiles of nearly 2,500 adsorbed proteins across 817 nanoparticle formulations (192). Meta-analysis revealed that silica, polystyrene, and lipid-based nanoparticles smaller than 100 nm with moderately negative to neutral zeta potentials preferentially bind apolipoproteins APOE and APOB-100, proteins linked to receptor-mediated uptake and enhanced delivery efficiency. Such database-driven insights enable prospective design of nanoparticles with favorable corona compositions for enhanced tumor targeting. Predicting cellular uptake represents another critical application. Alafeef et al. applied ML to estimate internalization behavior of carbon nanoparticles across different cancer cell lines, demonstrating that computational models can accurately predict uptake efficiency based on physicochemical properties (170). For biodistribution prediction, Chou et al. developed an AI-assisted physiologically-based pharmacokinetic model integrating ML with mechanistic modeling to predict nanoparticle delivery to tumors in mice (171). Mi et al. further developed ML models predicting tissue distribution and tumor delivery efficiency, enabling prospective selection of formulations likely to achieve therapeutic concentrations in target tissues (193). Digital twin technology represents an emerging application enabling real-time simulation and optimization of drug delivery processes. By creating computational replicas of physical systems that update based on sensor data, digital twins can guide personalized treatment optimization and predict individual patient responses to nanoparticle-mediated therapy (194). Recent applications have reduced formulation optimization timelines from months to weeks while achieving enhanced stability and performance.

#### 4.3.3 Optimizing nanocarriers for PDT-specific applications

The unique requirements of PDT, including efficient PS delivery, appropriate subcellular localization, and adequate light activation, create specialized demands for nanocarrier optimization. Several recent studies have demonstrated AI-driven optimization of nanoparticles specifically for phototherapeutic applications. Upconversion nanoparticles (UCNPs) address the fundamental limitation of light penetration depth in PDT by enabling NIR excitation and visible light emission for PS activation in deep tissues (195). These lanthanide-doped nanostructures can absorb NIR light (800–1000 nm) and emit UV/visible light capable of activating conventional PS, extending PDT applicability to tumors beyond the reach of direct visible light irradiation (196). Computational approaches can optimize UCNP composition and surface functionalization to maximize energy transfer efficiency to PSs while maintaining biocompatibility and tumor targeting capabilities. Smart nanoplatforms responding to tumor microenvironment stimuli represent another area of active development. pH-responsive, redox-responsive, and enzyme-responsive nanocarriers enable triggered release of PSs specifically within tumor tissues, minimizing off-target photosensitivity (197). ML models can predict stimulus-response characteristics based on nanocarrier composition, enabling rational design of platforms with optimal release kinetics for specific tumor types. Zhou et al. reviewed how nanogenerator strategies can overcome barriers in PDT, highlighting opportunities for AI-driven optimization of these emerging platforms (184). The integration of multiple therapeutic modalities within single nanoplatforms, combining PDT with chemotherapy, photothermal therapy (PTT), or immunotherapy, creates additional optimization challenges that benefit from AI approaches. Varon et al. developed predictive models for nanotechnology-based PDT combined with PTT, using ML to optimize laser radiation parameters for enhanced treatment efficiency (174). Their analytical function fitting (AFF) models identified optimal laser intensity and duration settings, establishing foundations for personalized combination therapy optimization where multiple treatment parameters must be simultaneously tuned.

#### 4.3.4 Generative AI for novel nanocarrier design

Generative AI models, including GANs and VAEs, represent an emerging frontier in nanocarrier design by enabling *de novo* generation of novel molecular structures with desired properties (158). While extensively developed for small molecule drug design, these approaches are increasingly applied to nanomedicine formulation development with promising results. GANs have been employed to generate novel ionizable lipid structures for nanoparticle-mediated delivery, producing lipids with optimized pKa values and lipophilicity for enhanced endosomal escape (198). Out of over 1200 generated lipids, approximately 90% were synthesizable within three synthetic steps, and the top candidates demonstrated superior mRNA binding affinity compared to clinically approved lipids. This generative approach accelerates the exploration of chemical space far beyond what traditional screening methods can achieve. The directed evolution framework introduced by Shan et al. combines virtual and physical compound libraries with ML-driven analysis to iteratively refine nanoparticle designs (199). This approach merges combinatorial synthesis, DNA/peptide barcoding for high-throughput *in vivo* screening, and computational prediction to identify structure-activity relationships guiding optimization. Such integrated workflows represent the future of nanocarrier development, where AI continuously learns from experimental feedback to propose improved candidates. RL offers complementary capabilities by iteratively refining nanoparticle designs through feedback loops that reward configurations meeting performance criteria such as enhanced tumor targeting, reduced immune clearance, or optimal drug release kinetics (200). By learning optimal design strategies through trial-and-error interaction with simulated or real experimental environments, RL agents can navigate complex multi-objective optimization landscapes that challenge conventional approaches. The convergence of generative design, high-throughput experimentation, and autonomous synthesis platforms promises to accelerate nanocarrier development for PDT applications substantially.

### 4.4 Monitoring treatment responses

**Figure 7:**
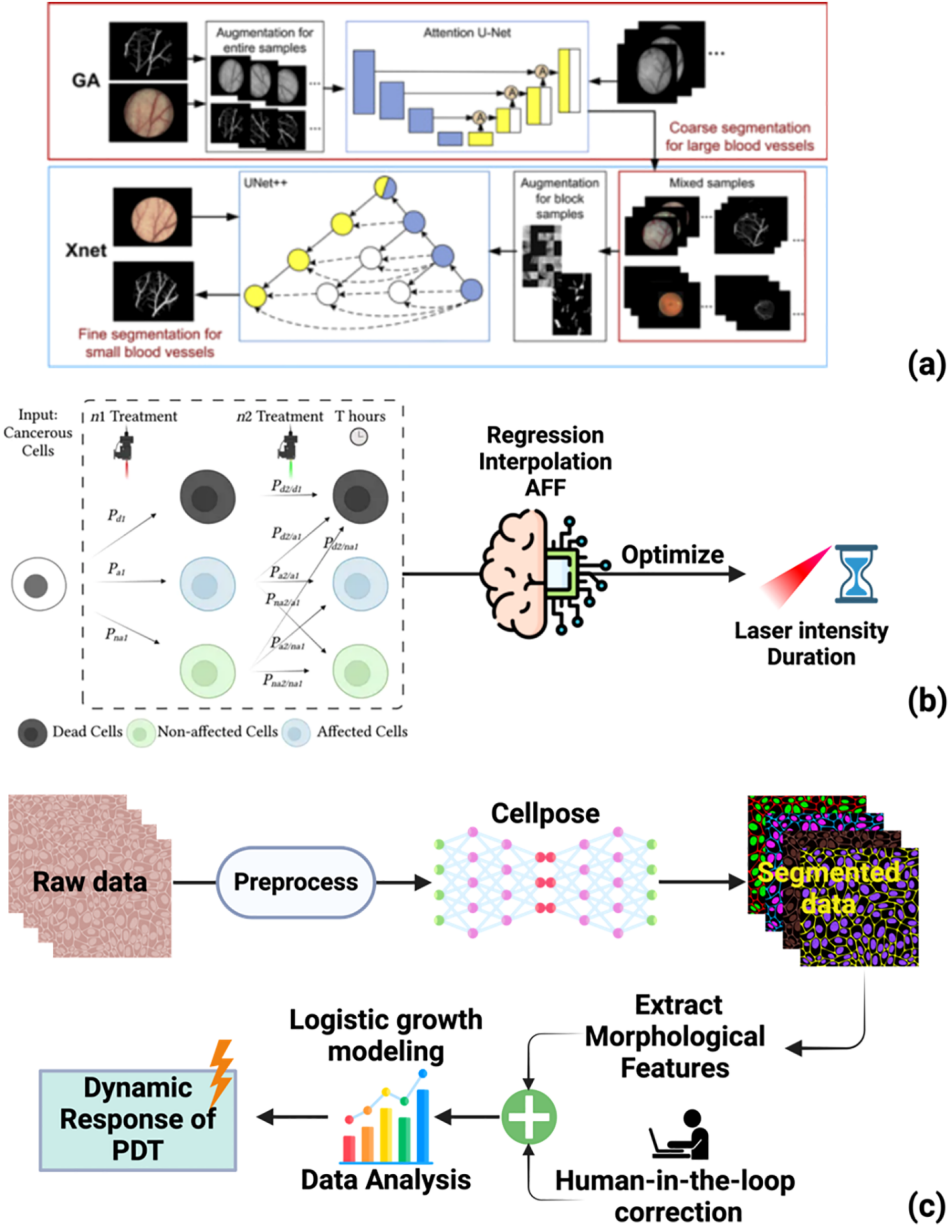
Machine learning approaches for monitoring PDT treatment responses. **(a)** GA-Xnet model combines global feature learning with Attention U-Net (GA step) and multiscale feature extraction with UNet++ (Xnet step) for precise blood vessel segmentation in V-PDT; transfer learning from retinal vessel datasets enhances accuracy, sensitivity, and specificity for evaluating vascular responses. Adapted from (173). **(b)** Three predictive models (regression, interpolation, and analytical function fitting (AFF)) optimize laser intensity and duration for combined PDT and PTT based on cellular response data tracking dead, non-affected, and affected cells across treatment conditions. Adapted from Varon E, et. al. (174). **(c)** Cellpose-based pipeline processes raw fluorescence images through segmentation, morphological feature extraction, and human-in-the-loop correction; logistic growth modeling reveals dynamic cellular responses to PDT for treatment personalization. Adapted from (175).

Monitoring treatment responses during PDT enables clinicians to assess therapeutic efficacy and adjust parameters based on observed tissue or cellular changes. Individual variations in PS uptake, tumor microenvironment, and biological response affect treatment efficacy, making real-time and post-treatment monitoring essential for personalized therapy. DL approaches now enable automated segmentation of vascular structures, quantification of cellular responses, and tracking of morphological dynamics across treatment time points. Vascular-targeted PDT (V-PDT) requires precise assessment of blood vessel responses to evaluate therapeutic efficacy. Xu et al. introduced the Global Attention-Xnet (GA-Xnet) model, a multi-step deep neural network designed for segmentation of subfascial blood vessels in the dorsal skinfold window chamber model (173). Accurate vessel segmentation in this context is challenging due to complex tissue architecture and imaging artifacts. The GA-Xnet pipeline employs three sequential stages. First, a Hough transform combined with a U-Net model extracts circular regions of interest from images. Second, an Attention U-Net learns global features and performs coarse segmentation of blood vessels (the GA step). Third, the coarse segmentation results combined with retinal images from the DRIVE dataset are input to a UNet++ model that learns multiscale features and produces fine segmentation maps (the Xnet step). This multi-step approach combining global feature learning, attention mechanisms, and transfer learning from established retinal vessel datasets, addresses the challenge of limited V-PDT training data (**Figure 7a**). The model achieved high accuracy, sensitivity, and specificity for subfascial vessel segmentation, demonstrating potential for clinical application in evaluating V-PDT responses. Further studies will be needed to assess the feasibility of long-term response evaluation, but the approach establishes a foundation for quantitative vascular monitoring. While vascular segmentation captures tissue-level effects, cellular response monitoring provides direct insight into treatment efficacy. Varon et al. addressed the challenge of optimizing combined PDT and PTT by systematically monitoring cellular responses across laser conditions (174). They presented a methodology combining nanotechnology-based PDT and PTT with ML to optimize cancer treatment parameters. *In vitro* cytotoxicity assays gathered data on cell death induced by PDT and PTT using a single nanocomplex, with measurements of cell death after light radiation divided into training and test sets. Three predictive models were developed to determine optimal laser radiation intensity and duration that maximize treatment efficiency (**Figure 7b**). The regression model predicted relationships between input parameters (laser intensity and duration) and treatment outcome. The interpolation model estimated values within the training data range, providing continuous predictions based on existing data points. The AFF model approximated the response surface with a low-degree function to predict treatment outcomes. The models were used to identify optimal laser radiation intensity and duration settings that maximize treatment efficiency. Comparing prediction errors across models, the AFF approach offered the best balance of simplicity and accuracy, making it particularly suitable for clinical applicability. This model was subsequently used for sensitivity analysis, examining how treatment performance responds to parameter variations, aiding in the refinement of treatment protocols. The work establishes a foundation for personalized cancer treatment optimization where monitored cellular responses directly inform parameter selection.

Understanding dynamic morphological changes in cancer cells during PDT offers insights for treatment personalization. Rahman et al. demonstrated the potential of DL, particularly the Cellpose algorithm, to enhance understanding of PDT’s dynamic effects on cancer cells (175). The advanced instance segmentation provided by Cellpose offers detailed insights into cellular responses, enabling personalized treatment strategies (**Figure 7c**). They analyzed fluorescence microscopic time series images of hepatocellular carcinoma (HCC) cells exposed to different laser intensities (6%-12.5%). These images included 16,100 cells segmented using the pre-trained Cellpose model, allowing for comprehensive morphological characterization, including dimensions and geometric properties. The authors employed Cellpose, a deep learning model based on U-Net architecture, for robust instance segmentation of cancer cells. Cellpose is known for its ability to handle diverse cell morphologies and provides superior accuracy compared to traditional methods. It was chosen due to its resilience to noise and capability to handle a wide range of cell shapes and sizes, which is crucial for accurately monitoring PDT effects on HCC cells. The authors used a pre-trained version, leveraging transfer learning to adapt the model to their specific dataset without extensive retraining. This approach saved time and computational resources while maintaining high segmentation accuracy. Following segmentation, various morphological features, including length, width, area, perimeter, and diameter, were extracted using the scikit-image library. This detailed morphological characterization was essential for understanding cellular responses to PDT. A human-in-the-loop approach identified and rectified instances of missing cells during segmentation, with iterative manual correction and retraining ensuring high prediction accuracy and reliability. To predict cellular behavior post-PDT, logistic growth modeling was applied, providing insights into dynamic responses of cancer cells to different laser intensities and enhancing understanding of PDT efficacy. The study included extensive measurement and analysis of morphological attributes of all segmented cells, offering insights into physical dimensions and geometric properties. The combination of Cellpose-based segmentation and logistic growth modeling revealed significant morphological changes in HCC cells after PDT, such as changes in cell size, shape, and proliferation rates. These findings were critical for optimizing PDT parameters and improving treatment efficacy. The study highlights the challenges of measuring onset times of anti-cancer drugs and the limitations of traditional methods. Their findings underscore the potential of DL to improve cancer cell analysis and contribute to the development of more effective cancer therapies. These approaches address complementary aspects of PDT monitoring. GA-Xnet enables precise vascular segmentation for evaluating V-PDT responses. Systematic cytotoxicity measurement across laser conditions informs ML-driven parameter optimization for combined therapies. Cellpose-based morphological tracking reveals dynamic cellular changes that guide treatment personalization. Together, these capabilities advance the real-time and post-treatment assessment needed for adaptive PDT.

### 4.5 Predicting treatment outcomes

Predicting treatment outcomes before or after PDT enables tailored protocols, efficient resource allocation, and informed clinical decision-making. Unlike real-time monitoring that guides immediate parameter adjustments, outcome prediction addresses longer-term questions: which patients will respond to treatment, what factors influence recurrence, and how therapy can be personalized based on individual characteristics. AI models integrating imaging data with clinical variables have demonstrated significant predictive capability across diverse PDT applications.

**Figure 8:**
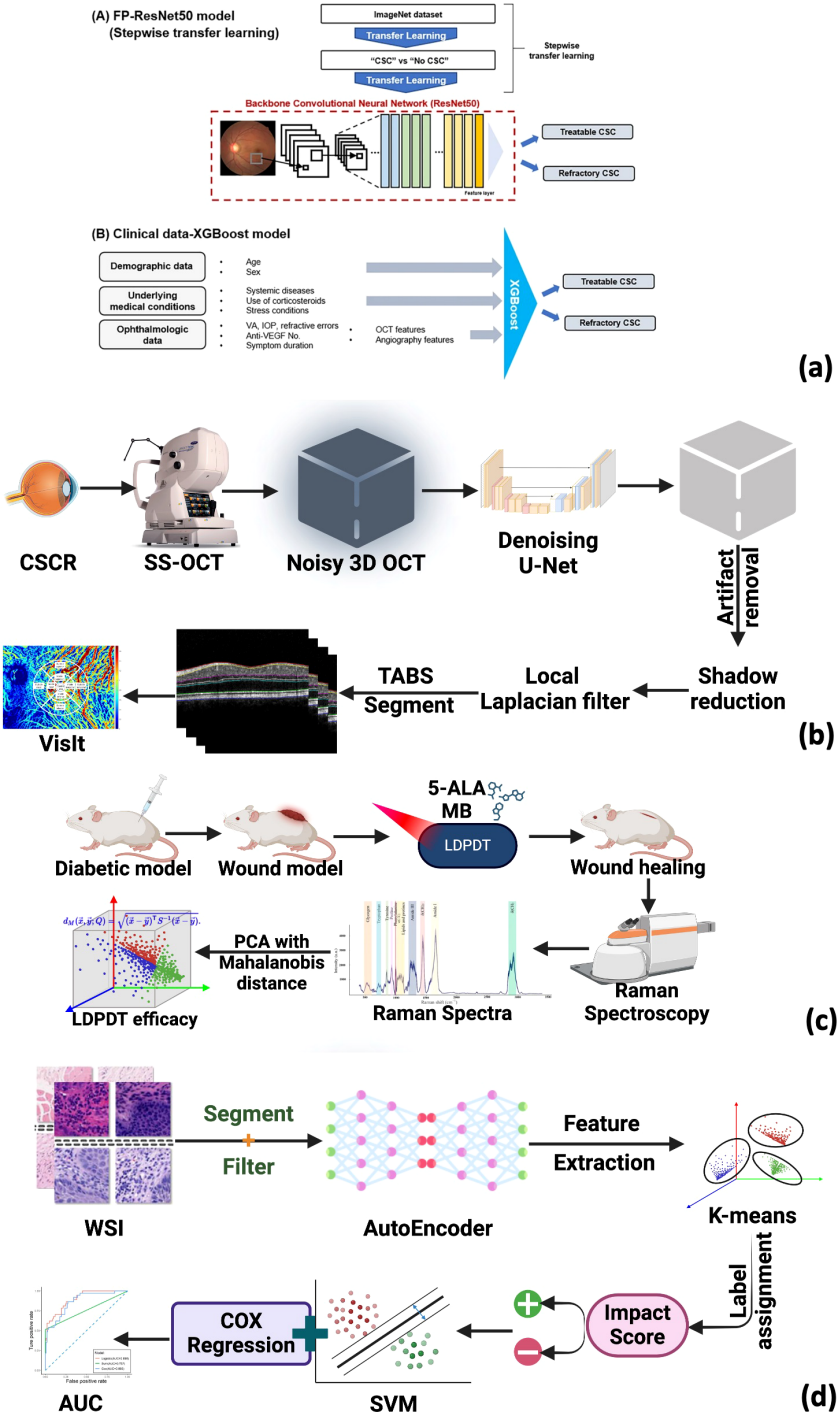
AI-based prediction of PDT treatment outcomes across clinical applications. **(a)** DeepPDT-Net predicts 1-year PDT outcomes for central serous chorioretinopathy (CSC) using a two-stage approach: ResNet50 extracts deep features from fundus photographs through stepwise transfer learning, while XGBoost integrates clinical variables including demographic, medical, and ophthalmologic data to classify treatable versus refractory cases. Adapted from Choi, J.Y., Kim, H., Kim, J.K. et al. (176)]. **(b)** 3D volumetric analysis of choroidal structure in CSC using SS-OCT with U-Net denoising, shadow reduction, local Laplacian filtering, and Topcon Advanced Boundary Segmentation (TABS); VisIt software enables 3D visualization of choroidal vessel and stromal volumes. Adapted from (178). **(c)** Low-dose PDT (LDPDT) assessment for diabetic wound healing using Raman spectroscopy with 5-aminolevulinic acid (5-ALA) and methylene blue (MB) PSs; PCA with Mahalanobis distance quantifies wound healing progression by analyzing spectral changes in amide I and CH_2_ bands. Adapted from (177). **(d)** Recurrence prediction for oral leukoplakia (OLK) following ALA-PDT: whole slide images (WSIs) are segmented and processed through an autoencoder for feature extraction, K-means clustering assigns impact scores for recurrence risk, and COX regression combined with clinical variables achieves high AUC for 12-month prediction. Adapted from (179).

Central serous chorioretinopathy (CSC) treatment with PDT presents a prediction challenge where identifying treatable versus refractory cases could guide therapeutic decisions. Yoo et al. developed DeepPDT-Net, a two-stage deep learning model predicting 1-year PDT outcomes using initial clinical data (176). Their dataset included 166 eyes with chronic CSC and 745 healthy control eyes. Clinical data encompassing demographic details, medical history, corticosteroid use, stress conditions, and ophthalmologic measurements, including fundus photographs (FPs), OCT measurements, and PDT protocol parameters. Data augmentation through flipping, translation, rotation, zooming, and shearing prevented overfitting, given the limited CSC dataset size. The first stage employed ResNet50 pretrained on ImageNet and fine-tuned to distinguish CSC from normal eyes using the larger dataset containing both patient and control FPs. The model was further fine-tuned on CSC eyes alone to differentiate treatable from refractory cases, adapting specifically to CSC-related features. For the combined approach, deep features extracted from FPs by the pretrained ResNet50 were combined with clinical variables using XGBoost, creating the DeepPDT-Net model that predicts the likelihood of complete subretinal fluid absorption after PDT (**Figure 8a**). Performance was evaluated using the area under the curve (AUC) and Youden’s index for optimal threshold setting. Shapley Additive Explanations (SHAP) analyzed the contribution of each input variable to model decisions, while Gradient-weighted Class Activation Mapping (Grad-CAM) generated heatmaps highlighting FP regions influencing predictions. The authors noted that while Grad-CAM provided insights into regions of interest, the heatmaps remained challenging to interpret and limited in clinical actionability, highlighting the ongoing gap between model explainability and clinical utility. While DeepPDT-Net focused on predicting treatment response, understanding the underlying choroidal changes in CSC requires detailed structural analysis. Previous studies relied primarily on 2D choroidal thickness measurements from swept-source OCT (SS-OCT), which may not capture the complex 3D structure involved in disease pathogenesis. Hara et al. introduced a novel AI-based method for 3D volumetric analysis of the choroid to better understand CSC and treatment response (178). Patients underwent SS-OCT before treatment and at 1 and 3 months post-treatment. A custom deep learning noise reduction algorithm utilizing U-Net was applied to original volumetric scans, followed by shadow reduction techniques addressing artifacts in deep choroidal layers. Attenuation compensation and contrast enhancement with a local Laplacian filter improved visibility of choroidal vessels. The preprocessed scans were segmented using the Topcon Advanced Boundary Segmentation algorithm with manual corrections as needed, and a fully automated composite method combining local and global thresholding accurately segmented choroidal vessels from stroma (**Figure 8b**). The segmented structures were visualized in 3D using VisIt software, enabling quantification of total choroidal volume, vessel volume, and stromal volume. This detailed 3D analysis provided insights into choroidal architecture changes with treatment that 2D measurements cannot capture, suggesting pathogenic mechanisms and enabling more precise response assessment.

Beyond ophthalmology, PDT outcome prediction extends to other clinical applications. Zuhayri et al. investigated low-dose PDT effects on diabetic wounds in mice using two PSs, 5-aminolevulinic acid and methylene blue, with laser doses of 1 J/cm² and 4 J/cm² (177). Raman spectroscopy provided non-invasive biochemical and structural analysis of tissue state. The study developed a quantitative assessment method applying PCA to Raman spectroscopy data to reduce dimensionality and identify patterns explaining variance across wound healing states (**Figure 8c**). PCA transformed potentially correlated spectral features into linearly uncorrelated principal components, enabling visualization of chemical content changes distinguishing wound states across study groups. The analysis revealed that specific principal components (PC2 and PC3) in the spectral range of 2800–3000 cm^-1^, corresponding to amide I and CH_2_ band intensities, were particularly effective for monitoring the healing process. Mahalanobis distance quantified differences between wound states and a healthy skin reference, providing a metric for assessing how closely treated wounds approached normal tissue characteristics. This approach enables objective, non-invasive tracking of wound healing progression that could guide treatment decisions. Oral leukoplakia (OLK) treated with aminolaevulinic acid PDT (ALA-PDT) presents a recurrence prediction challenge where identifying high-risk patients could enable intensified follow-up or adjuvant therapy. Wang et al. applied ML and DL to enhance the prediction of short-term efficacy and recurrence (179). Whole slide images (WSIs) of pathological sections were segmented into 128×128 pixel patches using Python’s Openslide, with filtering criteria removing contaminated or irrelevant patches. An autoencoder, trained for 50 epochs to minimize reconstruction loss, extracted meaningful features from the image patches in an unsupervised manner. K-means clustering grouped the extracted feature vectors, and each patch was labeled based on whether the patient experienced OLK recurrence after PDT (**Figure 8d**). Impact scores calculated for each cluster determined the likelihood of promoting or preventing recurrence, with scores above 0.5 indicating recurrence-associated features and below 0.5 indicating protective features. Pathologists assessed patches within each cluster to categorize them by histomorphological characteristics, providing interpretable connections between learned features and tissue biology. Predictive models combining deep learning-generated features with clinical variables were constructed using logistic regression, COX regression, and SVM, with 10-fold cross-validation for evaluation. Comparison of models using only WSIs, only clinical variables, or both demonstrated that combining data types significantly improved predictive performance, with the combined model achieving high accuracy and AUC values. The COX regression model showed good predictive performance for recurrence up to 12 months after PDT, demonstrating that integrated pathological and clinical features can inform recurrence risk assessment. These prediction studies span diverse clinical contexts but share common themes. Integration of imaging features with clinical variables consistently improves performance over either data type alone. DL enables the extraction of features from complex medical images that complement traditional clinical predictors. Extending these approaches to incorporate multimodal data and enable real-time adaptive treatment represents the next frontier.

## 5 Future research directions

### 5.1 Integration of multimodal imaging and data fusion

Current AI applications in PDT typically analyze single data streams, whether optical spectra, fluorescence images, or clinical variables, yet the complexity of tumor biology and treatment response demands more comprehensive characterization. Integrating data from multiple imaging modalities, including MRI for anatomical context, PET for metabolic activity, OCT for microstructural detail, and fluorescence imaging for PS distribution, would provide holistic views of tumor characteristics and the dynamic microenvironment. Deep learning architectures capable of processing and fusing these diverse data types, whether through late fusion of modality-specific predictions or attention-based integration of learned representations, represent a critical research frontier (201). The challenge extends beyond technical architecture to practical implementation. Multimodal approaches complicate training and require careful handling of missing data when not all modalities are available for every patient. Research must develop robust fusion strategies that degrade gracefully when inputs are incomplete, maintaining clinical utility even with partial information. Additionally, combining imaging data with molecular markers, including genetic, proteomic, and metabolomic profiles, would enable truly integrative tumor characterization, moving PDT planning beyond morphological assessment toward biologically-informed treatment design. Federated learning offers a pathway to realize these ambitions despite data fragmentation across institutions (202). By training models locally and aggregating only weight updates rather than sharing sensitive patient data, federated approaches enable multi-institutional collaboration while preserving privacy (203). This paradigm is particularly valuable for PDT, where individual centers may have limited patient volumes, but collective datasets could support sophisticated multimodal models. Research priorities include developing federated architectures optimized for heterogeneous imaging protocols and establishing standardized data representations that facilitate cross-institutional learning (204).

### 5.2 Real-time adaptive PDT systems

The static nature of current PDT protocols, where treatment parameters are determined pre-operatively and applied uniformly, fails to account for the dynamic tissue responses occurring during light delivery. Real-time adaptive systems that continuously monitor tissue state and adjust treatment parameters accordingly represent a paradigm shift toward truly responsive therapy. RL algorithms that learn optimal control policies from real-time feedback, adjusting light dosimetry and irradiance patterns based on continuous physiological monitoring, could maximize therapeutic effect while minimizing collateral damage (205, 206). Such approaches have shown promising results in retrospective dosimetric studies of analogous adaptive radiotherapy applications, where RL-based systems dynamically optimize dose fractionation based on tumor response (207). Implementation requires integration of real-time sensing technologies including optical, thermal, and biochemical sensors into PDT delivery systems, providing immediate feedback on tissue oxygenation, PS photobleaching, and cellular stress responses. The computational challenge lies in processing these multivariate signals rapidly enough to inform treatment adjustments within clinically relevant timeframes. Edge computing architectures that perform inference locally rather than relying on cloud connectivity may prove essential for achieving the latency requirements of intraoperative adaptation. Synthetic data generation through GANs addresses a fundamental barrier to developing adaptive systems: the limited availability of real-time monitoring data from diverse patient populations. GANs trained on existing tissue optical property datasets can generate realistic synthetic samples spanning parameter spaces underrepresented in clinical collections, enabling robust algorithm development prior to prospective validation. Research should focus on ensuring synthetic data captures the physiological variability and measurement noise characteristic of real clinical environments.

### 5.3 Personalized PDT protocol optimization

Moving beyond population-level treatment guidelines toward individualized protocols tailored to each patient’s tumor biology and systemic characteristics represents perhaps the most consequential opportunity for AI in PDT. Machine learning models analyzing genomic and proteomic data alongside imaging and clinical variables can identify patient subgroups with differential treatment responses, enabling precision medicine approaches to PDT planning. Pharmacogenomic insights may reveal genetic variants affecting PS metabolism, cellular susceptibility to oxidative stress, or immune response to PDT-induced immunogenic cell death. Foundation models pre-trained on massive chemical and biological datasets offer transformative potential for personalized PS selection and design (208, 209). These large-scale models learn universal molecular representations transferable to PDT-specific prediction tasks even with limited domain data. Incorporating three-dimensional molecular geometry and conformational dynamics into predictive frameworks, potentially through physics-informed neural networks embedding quantum mechanical principles for excited-state property prediction, would enhance accuracy for novel PS candidates (210). Multi-scale models integrating molecular-level predictions with cellular uptake, subcellular localization, and tissue distribution could optimize the entire PS-to-therapeutic-effect pathway. The vision of closed-loop automated laboratories, where robotic synthesis systems guided by machine learning iteratively design, synthesize, characterize, and refine PSs, is transitioning from speculation to early implementation (211, 212). Self-driving laboratories combining high-throughput experimentation with active learning algorithms that prioritize the most informative experiments could dramatically accelerate PS optimization cycles (213). Research should address integration challenges, including standardized characterization protocols, automated quality control, and feedback mechanisms linking *in vitro* results to computational model refinement.

### 5.4 AI-driven clinical decision support

Translating AI advances into clinical impact ultimately requires decision support tools that enhance rather than disrupt existing practice workflows. Developing interpretable models that provide not only predictions but also explanations accessible to clinicians represents a critical research priority (214). Techniques beyond gradient-weighted class activation mapping, including concept-based explanations, counterfactual reasoning, and uncertainty quantification, could help clinicians understand model recommendations and appropriately calibrate their trust (215, 216). Validation across diverse patient populations and clinical settings is essential before widespread deployment. Models developed at single institutions may fail to generalize when confronted with different imaging equipment, patient demographics, or clinical protocols. Research must establish robust frameworks for external validation, domain adaptation, and continuous model monitoring to detect performance degradation over time. Prospective clinical trials comparing AI-guided PDT protocols against standard-of-care will ultimately determine whether computational advances translate to improved patient outcomes. Integration pathways must consider clinical workflow constraints, regulatory requirements, and liability considerations. AI tools designed for seamless incorporation into existing electronic health records and treatment planning systems, with clear documentation of intended use and performance limitations, will facilitate adoption (217). Collaboration between AI researchers, PDT clinicians, and regulatory scientists is essential to navigate the evolving landscape of AI medical device oversight while maintaining the pace of innovation.

### 5.5 Research priorities and translational pathways

Unlike radiology, where over 1,200 FDA-authorized AI devices now operate within established infrastructure (218, 219), PDT lacks the foundational elements for AI translation. Most clinical PDT treatments are still administered with little more than application of a prescribed drug dose and timed light delivery, with no standardized dosimetry protocols across treatment centers (220). No benchmark datasets enable algorithm comparison, and no dedicated research consortium coordinates multi-institutional efforts. Addressing these infrastructure gaps represents the most urgent near-term priority. Realizing AI-empowered PDT requires coordinated progress across defined horizons informed by successful translations in analogous fields. Near-term efforts (1–3 years) should focus on establishing imaging protocol standardization through dedicated task groups within professional societies, building upon existing dosimetry guidelines (23), creating pilot multi-center data sharing agreements with standardized annotations, and curating initial retrospective datasets targeting 500-1,000 cases with harmonized acquisition parameters. The Medical Imaging and Data Resource Center, a joint ACR/RSNA/AAPM consortium that has accumulated over 150,000 imaging studies using FAIR data principles (221), provides a directly applicable governance template for PDT-specific infrastructure. Mid-term goals (3–5 years) include completing external multi-center retrospective validation across three or more institutions following established reporting guidelines (222), conducting prospective validation studies across diverse clinical sites, and engaging FDA through pre-submission meetings to establish evidence requirements for specific indications. The validation pathway proven for IDx-DR—the first autonomous AI diagnostic device—combined retrospective algorithm development with a prospective pivotal trial across 10 primary care sites, achieving FDA authorization within 89 days and demonstrating 87.2% sensitivity and 90.7% specificity for diabetic retinopathy detection (223). This hybrid retrospective-prospective model offers a directly applicable template for AI-PDT translation. Long-term objectives (5–10 years) encompass regulatory authorization through 510(k) or *De Novo* pathways, establishment of post-market surveillance programs, pursuit of reimbursement pathways (224), and deployment with continuous performance monitoring. Translational success requires academic-industry hybrid partnerships that maintain scientific rigor while enabling regulatory-grade development. Funding mechanisms including NIH SBIR/STTR programs, NIBIB Trailblazer Awards, and the NSF-NIH Smart Health Program support early-stage development, while ARPA-H’s PRECISE-AI initiative addresses the critical challenge of detecting and correcting AI algorithm drift post-deployment (225). Federated learning approaches enable multi-institutional collaboration while preserving data privacy (202, 204). **Figure 8** illustrates the development pipeline and current translational barriers; the priorities outlined here provide a temporal roadmap for addressing these challenges.

## 6 Challenges and limitations

### 6.1 Small dataset challenges

Despite the remarkable progress detailed in the preceding sections, significant barriers remain between algorithmic development and clinical implementation. One of the most persistent obstacles confronting AI applications in PDT is the scarcity of high-quality, annotated datasets. Unlike general computer vision tasks, where millions of labeled images are readily available (226, 227), PDT-specific datasets remain remarkably limited. This scarcity stems from the specialized nature of the treatment modality, heterogeneous imaging protocols across institutions, and resource-intensive expert annotation requirements. This scarcity is compounded by the diversity of PDT applications: datasets collected for ophthalmic PDT may have limited relevance for dermatologic or oncologic applications, fragmenting an already small data landscape. Models developed with limited data frequently exhibit overfitting, wherein they memorize training examples rather than learning generalizable patterns (228). This manifests as artificially inflated performance metrics during development that fail to translate to real-world clinical settings. In PDT, where treatment decisions carry significant implications for patient outcomes, such performance degradation poses unacceptable risks (229). Several strategies address these challenges with varying applicability. Transfer learning leverages models pre-trained on large general-purpose datasets, enabling reasonable performance even with limited PDT data (230). Studies have demonstrated that this approach can reduce prediction errors and enhance generalizability across different imaging configurations (148). However, fundamental differences between natural images and PDT-relevant data, including fluorescence imaging, OCT, and spectroscopic measurements, may limit feature transferability from non-medical domains (231). Data augmentation through geometric transformations and GANs offers another avenue for artificially expanding datasets, though concerns regarding the clinical validity of synthetic data persist (232, 233). The heterogeneity of PDT data presents additional complexity beyond mere quantity. Treatment protocols vary across institutions, PS differ in their optical properties and accumulation patterns, and light delivery systems span a range of configurations (3). This variability means that even datasets of nominally adequate size may not capture the full spectrum of clinical scenarios, potentially limiting model generalizability. Multi-center collaborations and standardized data collection protocols represent potential solutions. However, coordinating such efforts across institutions with different equipment, expertise, and regulatory environments remains challenging (234).

### 6.2 Interpretability and the black box problem

DL architectures frequently achieve predictive capabilities at the expense of interpretability. Neural networks with many layers and millions of parameters function as effective black boxes, producing outputs without transparent explanations of the reasoning process (235). In clinical settings where treatment decisions must be justified to patients, clinicians, and regulatory bodies, this opacity presents fundamental challenges (236). When an AI system recommends a specific PDT protocol or predicts treatment response, clinicians require an understanding of the factors driving these recommendations (237). Without such insight, practitioners cannot evaluate whether the model’s reasoning aligns with established clinical knowledge, identify potential errors, or appropriately calibrate their trust in the system’s outputs (238). This interpretability gap undermines AI integration into clinical workflows, regardless of their demonstrated accuracy on benchmark datasets. Explainable AI (xAI) methodologies have emerged in response to these concerns, offering techniques to elucidate model decision-making. Gradient-weighted Class Activation Mapping generates visual heatmaps highlighting influential image regions (239), revealing whether models attend to clinically relevant features such as subretinal fluid or retinal pigment epithelium changes in ophthalmic PDT applications (176). Similarly, SHAP values quantify individual feature contributions, enabling identification of influential clinical variables (240). However, current techniques remain limited in generating clinically actionable explanations. Heatmaps indicate which regions influenced predictions without explaining their pathophysiological relevance (128), and *post-hoc* explanations may not reflect true computational processes within the model (241). PGNNs represent a promising middle ground, incorporating domain knowledge of light-tissue interactions and photodynamic mechanisms into model architectures to constrain predictions within physically plausible bounds while maintaining interpretability (147). Such approaches have demonstrated improved accuracy in predicting tissue optical properties, a critical parameter for PDT planning. Beyond technical solutions, explanations must be tailored to end-user needs; what satisfies a computer scientist may differ substantially from what a treating clinician requires (242). Research on effective human-AI collaboration in medical decision-making remains nascent, and significant work is needed to understand how clinicians interpret, trust, and act upon AI-generated insights in PDT contexts (243).

### 6.3 Clinical translation gap

**Figure 9:**
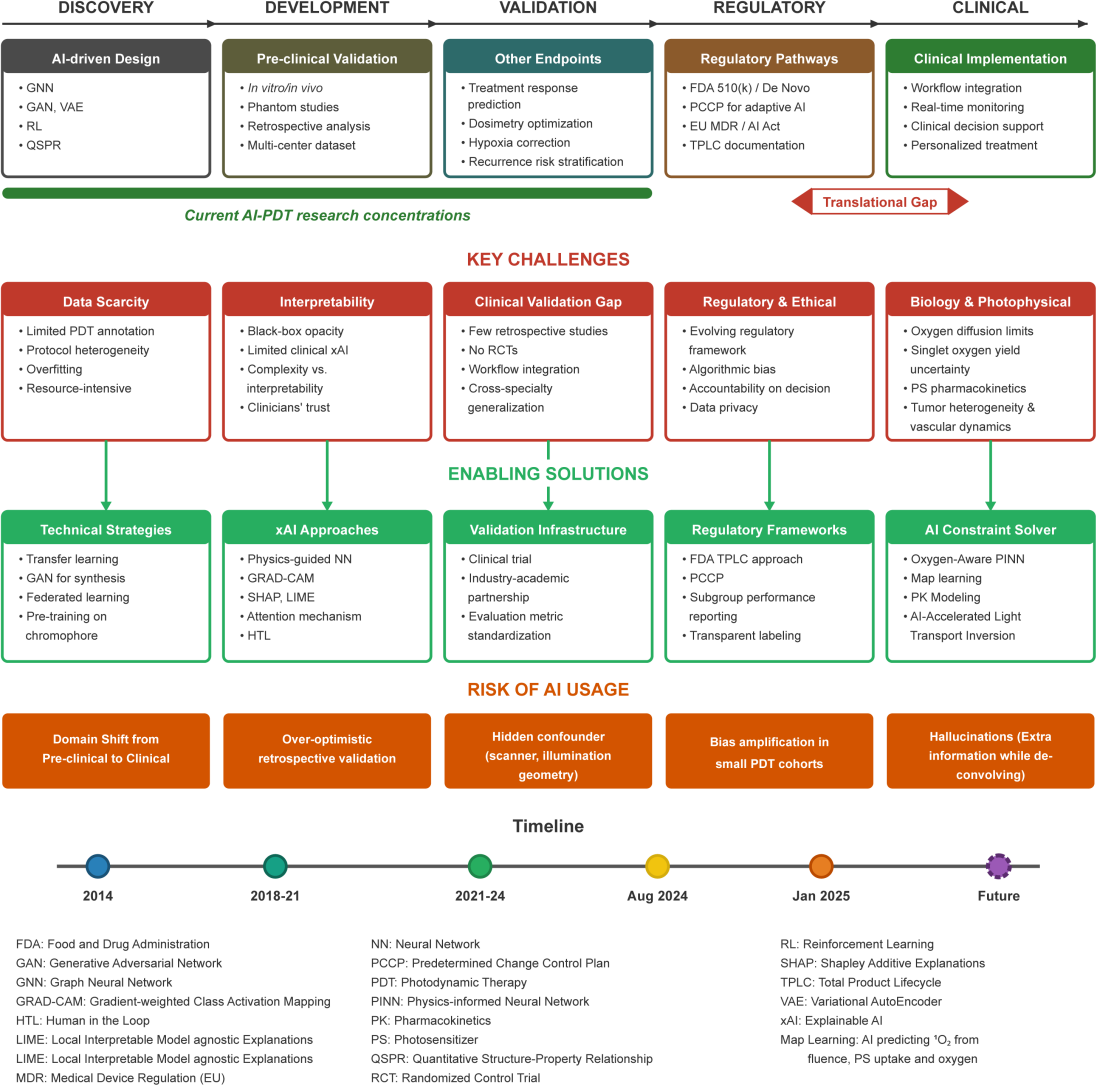
Translational roadmap for AI-empowered PDT. The figure illustrates the complete landscape of AI integration into PDT, spanning from discovery to clinical implementation. Top row: The AI-PDT development pipeline showing progression through five stages AI-driven molecular design (GNN, GAN, VAE, RL, QSPR), pre-clinical validation, endpoint optimization, regulatory pathways, and clinical implementation. The green bar indicates current research concentrations, while the translational gap between regulatory approval and clinical adoption represents a critical bottleneck. Middle rows: Key challenges facing AI-PDT translation (red) are mapped to enabling solutions (green), including technical strategies for data scarcity, xAI approaches for interpretability, validation infrastructure for clinical gaps, regulatory frameworks, and AI constraint solvers for biological/photophysical limitations. Bottom row: Risks associated with AI deployment in PDT contexts, including domain shift, over-optimistic validation, hidden confounders, bias amplification, and hallucinations. Timeline: Major milestones in AI-PDT development from 2014 to projected future advances. Abbreviations are defined below the figure.

A striking disparity characterizes AI in PDT: while preclinical and computational research flourish, validated clinical implementations remain conspicuously absent (224). This translational gap reflects fundamental barriers beyond typical research-to-practice lag. The preponderance of AI-PDT research has relied on retrospective designs utilizing existing clinical data or phantoms and animal models. While essential for method development, these approaches cannot substitute for prospective clinical evaluation and are susceptible to selection bias that may not reflect contemporary practice (244, 245). The retrospective studies that have examined AI in PDT applications, primarily in ophthalmologic conditions, represent encouraging initial steps but lack prospective validation (180). Clinical translation faces interconnected obstacles. First, PDT’s diversity across oncology, dermatology, and ophthalmology means models developed for one indication may not generalize to others (246). Unlike radiotherapy, where relatively standardized workflows enabled broader AI adoption (52), PDT protocol heterogeneity across disease sites, PSs, and light delivery systems fragments development efforts and prevents the accumulation of experience with any single platform. Second, real-time treatment monitoring applications require computational infrastructure for rapid inference, seamless integration with existing imaging and light delivery systems, and user interfaces accessible to clinicians without specialized AI expertise (247). Developing such integrated systems demands interdisciplinary collaboration among AI researchers, biomedical engineers, clinical physicists, and treating physicians that remains challenging to coordinate (248). Third, evidence standards for clinical adoption exceed those typically reported in technical publications. Demonstrations of accuracy on held-out test sets are insufficient to establish clinical utility (249). Clinicians appropriately require evidence that AI tools improve patient outcomes, fit within existing care pathways, and offer acceptable risk-benefit profiles (250). Generating such evidence requires randomized controlled trials or well-designed prospective studies demanding substantial time, resources, and clinical infrastructure. The lack of active AI-PDT clinical trials perpetuates a cycle where insufficient clinical experience generates inadequate data to train and validate AI tools, providing little impetus for adoption (251). Addressing this gap requires academic medical centers as research incubators, industry partnerships for integrated platforms, and professional consensus on evidence standards. Most importantly, it requires demonstration of clear clinical benefit in well-designed studies (252, 253). **Figure 9** synthesizes these multifaceted challenges into a comprehensive translational roadmap for AI-empowered PDT. The figure illustrates the development pipeline from AI-driven PS discovery through clinical implementation, mapping key barriers to corresponding enabling solutions. Data scarcity, interpretability limitations, clinical validation gaps, regulatory uncertainties, and biological constraints are each paired with targeted strategies. Technical approaches, including transfer learning, federated learning, and generative models, address small dataset challenges, while xAI methods such as PGNN, Grad-CAM, and SHAP offer pathways toward clinician trust. The roadmap also highlights deployment risks, including domain shift between preclinical and clinical settings, hidden confounders from imaging equipment variability, and bias amplification in small PDT cohorts. This integrated view underscores that successful clinical translation requires simultaneous progress across technical, regulatory, and validation domains.

### 6.4 Regulatory and ethical frameworks

PDT occupies an unusual regulatory position that presents distinct challenges for AI integration. Unlike most medical devices or drugs reviewed by a single FDA center, PDT systems are classified as cross-labeled combination products under 21 CFR 3.2(e), where the PS and light delivery device are separately packaged but specifically labeled for combined use (254). The PS’s pharmacological action constitutes the primary mode of action, placing the Center for Drug Evaluation and Research (CDER) as lead review center, with device components reviewed in consultation with CDRH (255). This bifurcated structure, established when porfimer sodium became the first approved PS in 1995, creates jurisdictional complexity that would be compounded by AI components requiring additional software oversight. Despite three decades of clinical experience, only six PSs have received FDA approval for limited indications, primarily in dermatology and ophthalmology (256). International regulators have authorized substantially broader applications; the EMA and Japan’s PMDA have approved agents for head and neck, lung, and esophageal cancers that remain unavailable in the United States (257, 258). This regulatory heterogeneity has direct implications for AI development: systems trained on European or Japanese clinical data incorporating these agents cannot be assumed valid for US submissions, while limited domestic indications constrain available on-label training data. Light delivery devices add further regulatory fragmentation. Laser-based PDT systems (FDA Product Code MVF) require Class III Premarket Approval (259). LED and lamp-based illuminators designed specifically for use with photosensitizing drugs such as the BLU-U, Aktilite CL128, and BF-RhodoLED, similarly require Class III authorization, typically as components of drug-device combination products (260). Non-coherent light devices for general phototherapy applications without PSs may receive Class II clearance through 510(k) under different product codes. The absence of FDA guidance specifically addressing PDT dosimetry compounds this fragmentation. Treatment parameters including light dose, irradiance, PS concentration, and drug-light interval vary substantially across institutions, with no standardized protocols analogous to those in radiotherapy (23). This variability is compounded by extensive off-label use documented across dermatology practices for indications including acne, photorejuvenation, and viral warts (261). For AI systems, training data inevitably incorporates these heterogeneous protocols, non-standardized dosimetry, and mixed on-label and off-label applications. An AI-enabled PDT platform integrating treatment planning, real-time dosimetry optimization, or outcome prediction would constitute an unprecedented three-component combination product spanning drug, device, and software jurisdictions. Such systems would likely require coordination between CDER, CDRH, and the Office of Combination Products, with formal Request for Designation advisable prior to development to establish the appropriate review pathway (262). The regulatory route would depend on how AI functionality integrates with existing components: whether as embedded dosimetry optimization within a light delivery device, standalone treatment planning software, or an integral system affecting drug dosing. The IMDRF Software as Medical Device framework provides risk categorization guidance, with AI controlling PDT parameters likely falling into Category III or IV given serious clinical consequences of dosimetry errors (263). Unlike radiology, where over 1,200 FDA-authorized AI devices, predominantly in radiology, with oncology and ophthalmology also represented, benefit from standardized imaging protocols and large annotated datasets (219, 264), PDT lacks this foundational infrastructure. The absence of benchmark datasets and obvious predicate devices combining PDT with AI suggests the *De Novo* pathway may be necessary for most AI-PDT applications (265).

Traditional regulatory frameworks designed for static medical devices are poorly suited to AI systems that must learn from variable real-world data while adapting over time (266). The FDA’s January 2025 draft guidance addresses this mismatch by establishing a Total Product Lifecycle approach, recognizing that AI devices require oversight not only at initial authorization but throughout operational lifespan, requiring documentation of model architecture, training data provenance, validation methodology, and performance monitoring throughout operational lifespan (267). For AI-PDT applications, compliance demands systematic characterization of training data sources, including explicit documentation of what proportion derives from off-label use, which PSs and light sources are represented, and how dosimetry parameters were recorded. Most authorized AI devices have received clearance through the 510(k) pathway based on substantial equivalence to predicate devices (265), while novel technologies have utilized the *De Novo* pathway (268). Understanding which pathway is appropriate for a given AI-PDT application and what evidence will be required is essential for developers planning regulatory strategies. The Predetermined Change Control Plan framework offers pathways for AI devices requiring post-market updates (269). Manufacturers can specify in advance allowable algorithm modifications and governing validation procedures. For AI-PDT tools incorporating real-time learning from treatment outcomes or periodic retraining as protocols evolve, this pathway may prove essential for maintaining compliance while enabling improvement. However, interaction between PCCP provisions for AI components and existing regulatory status of underlying PSs and devices remains subject to evolving interpretation (268). Labeling requirements have become increasingly specific, with implications for how AI-PDT developers must communicate inherent limitations. The FDA’s 2025 guidance recommends that labeling clearly indicate AI use, provide plain-language algorithm descriptions, detail model inputs and outputs, describe training data characteristics, report performance metrics, and disclose known limitations and bias sources (219). For AI-PDT devices trained on data from multiple PSs, varied light sources, or mixed indications, these requirements necessitate clear communication about which clinical scenarios have been validated and where generalizability may be limited. International harmonization remains incomplete, presenting additional challenges. The EU framework operates under the Medical Device Regulation and AI Act, with divergent approved PSs meaning systems trained on European data may not translate directly to US submissions (270). Differences in classification criteria, evidence requirements, and post-market surveillance obligations between jurisdictions require developers to navigate multiple regulatory pathways, potentially delaying the availability of beneficial technologies.

Beyond regulatory compliance, several ethical considerations warrant attention. Accountability for adverse outcomes from AI-assisted PDT involves multiple parties: clinician, institution, PS manufacturer, device manufacturer, and AI developer. Current malpractice frameworks designed around human decision-makers may inadequately address this distributed responsibility (271, 272). Algorithmic bias presents particular concern. Analysis reveals less than one-third of FDA-authorized AI device evaluations provide sex-specific performance data, and only one-fourth address age-related subgroups (265). For AI-PDT, where skin pigmentation influences fluorescence imaging quality and PS visualization, ensuring equitable performance across diverse populations is both an ethical imperative and a practical necessity (273). The historical concentration of PDT trials in fair-skinned populations for conditions like actinic keratoses may introduce systematic bias that developers must actively address. Data privacy further intersects with development needs, as training robust AI-PDT models requires large multi-institutional datasets subject to HIPAA and GDPR protections (274). Federated learning enables collaboration without sharing raw patient data, potentially allowing institutions with different protocols and PS preferences to contribute while preserving privacy (275, 276). Effective clinical translation requires attention to human-AI interaction. Clinical decision support systems must foster appropriate reliance, where clinicians neither ignore beneficial recommendations nor follow them blindly (277). For PDT, where treatment decisions integrate patient factors, lesion characteristics, and dosimetry considerations, designing interfaces that support rather than supplant clinical judgment requires careful attention to how recommendations are presented and how clinicians are trained to interpret outputs (278). In this context, xAI transitions from a desirable feature to a necessity (279–284). A clinician must understand whether an AI’s recommended dose is driven primarily by lesion size, fluorescence intensity, or historical outcome patterns from similar patients. Saliency maps highlighting which image regions most influenced a recommendation, or counterfactual explanations showing how different inputs would change the output, can foster appropriate trust and facilitate error detection (285–287). The path to clinical translation for AI-PDT systems requires a proactive, parallel development strategy. Future efforts should focus on creating consortia to establish benchmark datasets with standardized annotation protocols for imaging, dosimetry parameters, and outcomes across diverse skin types. Digital phantoms and simulation platforms can augment scarce clinical data for initial algorithm training. Engaging with regulators early via the FDA’s Pre-Submission program offers opportunities to gain alignment on validation strategies for hybrid combination products. Adaptive clinical trial designs that can evaluate both the AI component and its interaction with the PDT combination product in iterative phases may prove essential for navigating the unique regulatory landscape these systems present. As AI-PDT applications mature from prototypes to clinical tools, institutional quality management systems addressing validation, monitoring, and bias detection must develop in parallel (288).

## 7 Conclusion

The historical evolution of PDT from ancient practices to modern clinical treatment highlights its potential and adaptability in oncology and beyond. PDT’s continued advancement, driven by innovations in PS design, imaging technologies, and delivery systems, underscores its growing importance in the medical field. Concurrently, the rapid progression of ML and DL offers unprecedented opportunities to enhance PDT’s efficacy and precision. Integrating AI with PDT can significantly improve tissue characterization, PS discovery, treatment monitoring, and outcome prediction, leading to personalized and optimized therapeutic approaches. Given the explosion of research in these two dynamic fields, this review aimed to synthesize existing knowledge, identify gaps, and provide guidance for future research that would be valuable for researchers, clinicians, and policymakers. However, realizing this potential requires addressing the challenges outlined here: data scarcity, interpretability limitations, translational barriers, and evolving regulatory frameworks. We hope that highlighting the synergy between AI and PDT will promote collaborative research efforts. Researchers in computer science, oncology, radiology, and bioinformatics must work together to bridge the gap between algorithmic innovation and clinical implementation. By addressing these technical challenges and exploring synergistic advancements, we can pave the way for more effective and individualized PDT treatments, ultimately improving patient outcomes and expanding the therapeutic potential of this versatile modality.

## References

[1] Dougherty TJ Gomer CJ Henderson BW Jori G Kessel D Korbelik M . Photodynamic therapy. J Natl Cancer Inst. (1998) 90:889–905. doi: 10.1093/jnci/90.12.889, PMID: 9637138 PMC4592754

[2] Chilakamarthi U Giribabu L . Photodynamic therapy: past, present and future. Chem Rec. (2017) 17:775–802. doi: 10.1002/tcr.201600121, PMID: 28042681

[3] Mallidi S Spring BQ Hasan T . Optical imaging, photodynamic therapy and optically triggered combination treatments. Cancer J. (2015) 21:194–205. doi: 10.1097/PPO.0000000000000117, PMID: 26049699 PMC4459538

[4] Kessel D . Photodynamic therapy: A brief history. J Clin Med. (2019) 8:1581. doi: 10.3390/jcm8101581, PMID: 31581613 PMC6832404

[5] Ackroyd R Kelty C Brown N Reed M . The history of photodetection and photodynamic therapy. Photochem Photobiol. (2001) 74:656–69. doi: 10.1562/0031-8655(2001)0740656THOPAP2.0.CO2 11723793

[6] Dolmans DE Fukumura D Jain RK . Photodynamic therapy for cancer. Nat Rev Cancer. (2003) 3:380–7. doi: 10.1038/nrc1071, PMID: 12724736

[7] dos Santos AF de Almeida DQ Terra LF Baptista MS Labriola L . Photodynamic therapy in cancer treatment-an update review. J Cancer Metastasis Treat. (2019) 5:25. doi: 10.20517/2394-4722.2018.83, PMID: 39698022

[8] Silva JN Filipe P Morliere P Maziere JC Freitas JP Gomes MM . Photodynamic therapy: dermatology and ophthalmology as main fields of current applications in clinic. BioMed Mater Eng. (2008) 18:319–27. doi: 10.3233/BME-2008-0546, PMID: 19065042

[9] Daniell MD Hill JS . A history of photodynamic therapy. Aust N Z J Surg. (1991) 61:340–8. doi: 10.1111/j.1445-2197.1991.tb00230.x, PMID: 2025186

[10] Patel P Sanghvi S Patel P . The vedic view of vitiligo. JAMA Dermatol. (2018) 154:434. doi: 10.1001/jamadermatol.2018.0364, PMID: 29641830

[11] Moghissi K Allison RR . 1 - a Narrative History of Photodynamic Therapy☆. In Kesharwani P , editor. Nanomaterials for Photodynamic Therapy. Woodhead Publishing (2023). p. 1–39. doi: 10.1016/B978-0-323-85595-2.00010-4, PMID:

[12] Blum HF . Photodynamic action. Physiol Rev. (1932) 12:23–55. doi: 10.1152/physrev.1932.12.1.23, PMID: 40884160

[13] Lipson RL Baldes EJ . The photodynamic properties of a particular hematoporphyrin derivative. Arch Dermatol. (1960) 82:508–16. doi: 10.1001/archderm.1960.01580040026005, PMID: 13762615

[14] Lipson RL Baldes EJ Olsen AM . The use of a derivative of hematoporphyrin in tumor detection23. JNCI: J Natl Cancer Institute. (1961) 26:1–11. doi: 10.1093/jnci/26.1.1 13762612

[15] Hamblin MR . Photodynamic therapy for cancer: what’s past is prologue. Photochem Photobiol. (2020) 96:506–16. doi: 10.1111/php.13190, PMID: 31820824 PMC7282978

[16] Psomadakis CE Marghoob N Bleicher B Markowitz O . Optical coherence tomography. Clin Dermatol. (2021) 39:624–34. doi: 10.1016/j.clindermatol.2021.03.008, PMID: 34809767

[17] Xavierselvan M Cook J Duong J Diaz N Homan K Mallidi S . Photoacoustic nanodroplets for oxygen enhanced photodynamic therapy of cancer. Photoacoustics. (2022) 25:100306. doi: 10.1016/j.pacs.2021.100306, PMID: 34917471 PMC8666552

[18] Langley A Sweeney A Shethia RT Bednarke B Wulandana F Xavierselvan M . Heterogeneous tumor blood oxygenation dynamics during phototherapy deciphered with real-time label-free photoacoustic imaging. NPJ Acoustics. (2025) 1:9. doi: 10.1038/s44384-025-00012-x, PMID: 40485754 PMC12137135

[19] Celli JP Spring BQ Rizvi I Evans CL Samkoe KS Verma S . Imaging and photodynamic therapy: mechanisms, monitoring, and optimization. Chem Rev. (2010) 110:2795–838. doi: 10.1021/cr900300p, PMID: 20353192 PMC2896821

[20] Ji B Wei M Yang B . Recent advances in nanomedicines for photodynamic therapy (Pdt)-driven cancer immunotherapy. Theranostics. (2022) 12:434–58. doi: 10.7150/thno.67300, PMID: 34987658 PMC8690913

[21] Hafiz SS Xavierselvan M Gokalp S Labadini D Barros S Duong J . Eutectic gallium–indium nanoparticles for photodynamic therapy of pancreatic cancer. ACS Appl Nano Materials. (2022) 5:6125–39. doi: 10.1021/acsanm.1c04353, PMID: 35655927 PMC9150699

[22] Li G Wang C Jin B Sun T Sun K Wang S . Advances in smart nanotechnology-supported photodynamic therapy for cancer. Cell Death Discov. (2024) 10:466. doi: 10.1038/s41420-024-02236-4, PMID: 39528439 PMC11554787

[23] Zhu TC Pogue BW Dimofte A Finlay JC Lilge L Sunar U . Aapm task group report 274: fluence rate dosimetry for photodynamic therapy (Pdt). Med Phys. (2025) 52:1354–71. doi: 10.1002/mp.17613, PMID: 39815459 PMC11882379

[24] Cramers P Ruevekamp M Oppelaar H Dalesio O Baas P Stewart FA . Foscan^®^ Uptake and tissue distribution in relation to photodynamic efficacy. Br J Cancer. (2003) 88:283–90. doi: 10.1038/sj.bjc.6600682, PMID: 12610515 PMC2377038

[25] Wilson BC Patterson MS Lilge L . Implicit and explicit dosimetry in photodynamic therapy: A new paradigm. Lasers Med Sci. (1997) 12:182–99. doi: 10.1007/BF02765099, PMID: 20803326

[26] Alpaydin E . Machine Learning. 4th ed. Cambridge, MA: MIT press (2021).

[27] Kotsiantis SB . Supervised Machine Learning: A Review of Classification Techniques. In: Maglogiannis I Karpouzis K Bramer M , editors. Emerging Artificial Intelligence Applications in Computer Engineering. IOS Press (2007). p.3–24.

[28] Mahesh B . Machine learning algorithms-a review. Int J Sci Res (IJSR). (2020) 9:381–6. doi: 10.21275/ART20203995

[29] Guo G Wang H Bell D Bi Y Greer K . Knn model-based approach in classification. In: Meersman R Tari Z Schmidt DC , editors. On The Move to Meaningful Internet Systems 2003: CoopIS, DOA, and ODBASE: OTM Confederated International Conferences, CoopIS, DOA, and ODBASE. LNCS 2888. Berlin: Springer. (2003) 986–96. doi: 10.1007/978-3-540-39964-3_62, PMID:

[30] Maćkiewicz A Ratajczak W . Principal components analysis (Pca). Comput Geosciences. (1993) 19:303–42. doi: 10.1016/0098-3004(93)90090-R

[31] Rigatti SJ . Random forest. J Insur Med. (2017) 47:31–9. doi: 10.17849/insm-47-01-31-39.1, PMID: 28836909

[32] Wang H Hu D . Comparison of Svm and Ls-Svm for regression. In: 2005 International conference on neural networks and brain. Piscataway, NJ: IEEE. p. 279–83. doi: 10.1109/ICNNB.2005.1614615, PMID:

[33] Webb GI . Naïve bayes. In: Sammut C Webb GI , editors. Encyclopedia of machine learning. Boston, MA: Springer (2010). p. 713–4. doi: 10.1007/978-0-387-30164-8_576, PMID:

[34] Friedman JH . Greedy function approximation: A gradient boosting machine. Ann Stat. (2001) 29:1189–1232. doi: 10.1214/aos/1013203451, PMID: 38281721

[35] Chen T Guestrin C . XGBoost: A Scalable Tree Boosting System. In: Proceedings of the 22nd ACM SIGKDD International Conference on Knowledge Discovery and Data Mining. New York: ACM (2016). p.785–794. doi: 10.1145/2939672.2939785, PMID:

[36] Hosny A Parmar C Quackenbush J Schwartz LH Aerts HJWL . Artificial intelligence in radiology. Nat Rev Cancer. (2018) 18:500–10. doi: 10.1038/s41568-018-0016-5, PMID: 29777175 PMC6268174

[37] Bhatt C Kumar I Vijayakumar V Singh KU Kumar A . The state of the art of deep learning models in medical science and their challenges. Multimedia Syst. (2021) 27:599–613. doi: 10.1007/s00530-020-00694-1, PMID: 41865474

[38] Menghani G . Efficient deep learning: A survey on making deep learning models smaller, faster, and better. ACM Computing Surveys. (2023) 55:1–37. doi: 10.1145/3578938, PMID: 40727313

[39] Mosavi A Ardabili S Varkonyi-Koczy AR eds. List of deep learning models. In: Várkonyi-Kóczy AR , editor. Engineering for Sustainable Future. Cham: Springer International Publishing (2020). p. 202 14. 10.1007/978-3-030-36841-8_20, PMID:

[40] Tsuneki M . Deep learning models in medical image analysis. J Oral Biosci. (2022) 64:312–20. doi: 10.1016/j.job.2022.03.003, PMID: 35306172

[41] Vaswani A Shazeer N Parmar N Uszkoreit J Jones L Gomez AN . Attention is all you need, in: Advances in Neural Information Processing Systems 30 (NeurIPS 2017), Red Hook, NY: Curran Associates Inc. (2017) pp. 5998–6008. doi: 10.48550/arXiv.1706.03762, PMID:

[42] Kim M Yun J Cho Y Shin K Jang R Bae HJ . Deep learning in medical imaging. Neurospine. (2019) 16:657–68. doi: 10.14245/ns.1938396.198, PMID: 31905454 PMC6945006

[43] Lee JG Jun S Cho YW Lee H Kim GB Seo JB . Deep learning in medical imaging: general overview. Korean J Radiol. (2017) 18:570–84. doi: 10.3348/kjr.2017.18.4.570, PMID: 28670152 PMC5447633

[44] Shen D Wu G Suk HI . Deep learning in medical image analysis. Annu Rev BioMed Eng. (2017) 19:221–48. doi: 10.1146/annurev-bioeng-071516-044442, PMID: 28301734 PMC5479722

[45] Suzuki K . Overview of deep learning in medical imaging. Radiol Phys Technol. (2017) 10:257–73. doi: 10.1007/s12194-017-0406-5, PMID: 28689314

[46] Vamathevan J Clark D Czodrowski P Dunham I Ferran E Lee G . Applications of machine learning in drug discovery and development. Nat Rev Drug Discov. (2019) 18:463–77. doi: 10.1038/s41573-019-0024-5, PMID: 30976107 PMC6552674

[47] Shortliffe EH Sepúlveda MJ . Clinical decision support in the era of artificial intelligence. JAMA. (2018) 320:2199–200. doi: 10.1001/jama.2018.17163, PMID: 30398550

[48] Almeida G Tavares J . Deep learning in radiation oncology treatment planning for prostate cancer: A systematic review. J Med Syst. (2020) 44:179. doi: 10.1007/s10916-020-01641-3, PMID: 32862251

[49] Fan J Wang J Chen Z Hu C Zhang Z Hu W . Automatic treatment planning based on three-dimensional dose distribution predicted from deep learning technique. Med Phys. (2019) 46:370–81. doi: 10.1002/mp.13271, PMID: 30383300

[50] Liu F Yadav P Baschnagel AM McMillan AB . Mr-based treatment planning in radiation therapy using a deep learning approach. J Appl Clin Med Phys. (2019) 20:105–14. doi: 10.1002/acm2.12554, PMID: 30861275 PMC6414148

[51] Wang M Zhang Q Lam S Cai J Yang R . A review on application of deep learning algorithms in external beam radiotherapy automated treatment planning. Front Oncol. (2020) 10:580919. doi: 10.3389/fonc.2020.580919, PMID: 33194711 PMC7645101

[52] Huynh E Hosny A Guthier C Bitterman DS Petit SF Haas-Kogan DA . Artificial intelligence in radiation oncology. Nat Rev Clin Oncol. (2020) 17:771–81. doi: 10.1038/s41571-020-0417-8, PMID: 32843739

[53] Elemento O Leslie C Lundin J Tourassi G . Artificial intelligence in cancer research, diagnosis and therapy. Nat Rev Cancer. (2021) 21:747–52. doi: 10.1038/s41568-021-00399-1, PMID: 34535775

[54] Kolla L Parikh RB . Uses and limitations of artificial intelligence for oncology. Cancer. (2024) 130:2101–7. doi: 10.1002/cncr.35307, PMID: 38554271 PMC11170282

[55] Wilson BC Patterson MS . The physics, biophysics and technology of photodynamic therapy. Phys Med Biol. (2008) 53:R61–109. doi: 10.1088/0031-9155/53/9/R01, PMID: 18401068

[56] Zhou Z Song J Nie L Chen X . Reactive oxygen species generating systems meeting challenges of photodynamic cancer therapy. Chem Soc Rev. (2016) 45:6597–626. doi: 10.1039/c6cs00271d, PMID: 27722328 PMC5118097

[57] Garcia-Diaz M Huang YY Hamblin MR . Use of fluorescent probes for ros to tease apart type I and type II photochemical pathways in photodynamic therapy. Methods. (2016) 109:158–66. doi: 10.1016/j.ymeth.2016.06.025, PMID: 27374076 PMC5075498

[58] Huang L Xuan Y Koide Y Zhiyentayev T Tanaka M Hamblin MR . Type I and type II mechanisms of antimicrobial photodynamic therapy: an *in vitro* study on gram-negative and gram-positive bacteria. Lasers Surg Med. (2012) 44:490–9. doi: 10.1002/lsm.22045, PMID: 22760848 PMC3428129

[59] Castano AP Demidova TN Hamblin MR . Mechanisms in photodynamic therapy: part one—Photosensitizers, photochemistry and cellular localization. Photodiagnosis Photodyn Ther. (2004) 1:279–93. doi: 10.1016/S1572-1000(05)00007-4, PMID: 25048432 PMC4108220

[60] Abrahamse H Hamblin MR . New photosensitizers for photodynamic therapy. Biochem J. (2016) 473:347–64. doi: 10.1042/BJ20150942, PMID: 26862179 PMC4811612

[61] Chen D Xu Q Wang W Shao J Huang W Dong X . Type I photosensitizers revitalizing photodynamic oncotherapy. Small. (2021) 17:e2006742. doi: 10.1002/smll.202006742, PMID: 34038611

[62] Wang YY Liu YC Sun HW Guo DS . Type I photodynamic therapy by organic-inorganic hybrid materials: from strategies to applications. Coordination Chem Rev. (2019) 395:46–62. doi: 10.1016/j.ccr.2019.05.016, PMID: 41865745

[63] Zhuang Z Dai J Yu M Li J Shen P Hu R . Type I photosensitizers based on phosphindole oxide for photodynamic therapy: apoptosis and autophagy induced by endoplasmic reticulum stress. Chem Sci. (2020) 11:3405–17. doi: 10.1039/d0sc00785d, PMID: 34745515 PMC8515424

[64] Allison RR Downie GH Cuenca R Hu XH Childs CJ Sibata CH . Photosensitizers in clinical Pdt. Photodiagnosis Photodyn Ther. (2004) 1:27–42. doi: 10.1016/S1572-1000(04)00007-9, PMID: 25048062

[65] Gao JC Tian Y Li YG Hu F Wu WB . Design strategies for aggregation-induced emission photosensitizers with enhanced safety in photodynamic therapy. Coordination Chem Rev. (2024) 507:215756. doi: 10.1016/j.ccr.2024.215756, PMID: 41865745

[66] Garland MJ Cassidy CM Woolfson D Donnelly RF . Designing photosensitizers for photodynamic therapy: strategies, challenges and promising developments. Future Med Chem. (2009) 1:667–91. doi: 10.4155/fmc.09.55, PMID: 21426032

[67] Zhang P Steelant W Kumar M Scholfield M . Versatile photosensitizers for photodynamic therapy at infrared excitation. J Am Chem Soc. (2007) 129:4526–7. doi: 10.1021/ja0700707, PMID: 17385866 PMC2528873

[68] Zhao X Liu J Fan J Chao H Peng X . Recent progress in photosensitizers for overcoming the challenges of photodynamic therapy: from molecular design to application. Chem Soc Rev. (2021) 50:4185–219. doi: 10.1039/d0cs00173b, PMID: 33527104

[69] Kou J Dou D Yang L . Porphyrin photosensitizers in photodynamic therapy and its applications. Oncotarget. (2017) 8:81591–603. doi: 10.18632/oncotarget.20189, PMID: 29113417 PMC5655312

[70] Kwiatkowski S Knap B Przystupski D Saczko J Kedzierska E Knap-Czop K . Photodynamic therapy - mechanisms, photosensitizers and combinations. BioMed Pharmacother. (2018) 106:1098–107. doi: 10.1016/j.biopha.2018.07.049, PMID: 30119176

[71] Zhu W Gao Y-H Liao P-Y Chen D-Y Sun N-N Nguyen Thi PA . Comparison between porphin, chlorin and bacteriochlorin derivatives for photodynamic therapy: synthesis, photophysical properties, and biological activity. Eur J Medicinal Chem. (2018) 160:146–56. doi: 10.1016/j.ejmech.2018.10.005, PMID: 30336449

[72] Lo P-C Rodríguez-Morgade MS Pandey RK Ng DKP Torres T Dumoulin F . The unique features and promises of phthalocyanines as advanced photosensitisers for photodynamic therapy of cancer. Chem Soc Rev. (2020) 49:1041–56. doi: 10.1039/C9CS00129H, PMID: 31845688

[73] Algorri JF Lopez-Higuera JM Rodriguez-Cobo L Cobo A . Advanced light source technologies for photodynamic therapy of skin cancer lesions. Pharmaceutics. (2023) 15:2075. doi: 10.3390/pharmaceutics15082075, PMID: 37631289 PMC10458875

[74] Mang TS . Lasers and light sources for Pdt: past, present and future. Photodiagnosis Photodyn Ther. (2004) 1:43–8. doi: 10.1016/S1572-1000(04)00012-2, PMID: 25048063

[75] Maharjan PS Bhattarai HK . Singlet oxygen, photodynamic therapy, and mechanisms of cancer cell death. J Oncol. (2022) 2022:7211485. doi: 10.1155/2022/7211485, PMID: 35794980 PMC9252714

[76] Dąbrowski JM . Reactive oxygen species in photodynamic therapy: mechanisms of their generation and potentiation. In: van Eldik R Hubbard CD , editors. Advances in Inorganic Chemistry, vol. 70. Academic Press (2017). p. 343–94.

[77] Fuchs J Thiele J . The role of oxygen in cutaneous photodynamic therapy. Free Radic Biol Med. (1998) 24:835–47. doi: 10.1016/s0891-5849(97)00370-5, PMID: 9586814

[78] Xu F Wang M Dotse E Chow KT Lo P-C . Inducing immunogenic cancer cell death through oxygen-economized photodynamic therapy with nitric oxide-releasing photosensitizers. Angewandte Chemie Int Edition. (2024) 63:e202404561. doi: 10.1002/anie.202404561, PMID: 38887983

[79] Shen Z Ma Q Zhou X Zhang G Hao G Sun Y . Strategies to improve photodynamic therapy efficacy by relieving the tumor hypoxia environment. NPG Asia Materials. (2021) 13:39. doi: 10.1038/s41427-021-00303-1, PMID: 41862587

[80] Zhao L Fu C Tan L Li T Zhong H Meng X . Advanced nanotechnology for hypoxia-associated antitumor therapy. Nanoscale. (2020) 12:2855–74. doi: 10.1039/C9NR09071A, PMID: 31965135

[81] Larue L Myrzakhmetov B Ben-Mihoub A Moussaron A Thomas N Arnoux P . Fighting hypoxia to improve Pdt. Pharmaceuticals. (2019) 12:163 p. doi: 10.3390/ph12040163, PMID: 31671658 PMC6958374

[82] Castano AP Demidova TN Hamblin MR . Mechanisms in photodynamic therapy: part two—Cellular signaling, cell metabolism and modes of cell death. Photodiagnosis Photodyn Ther. (2005) 2:1–23. doi: 10.1016/S1572-1000(05)00030-X, PMID: 25048553 PMC4108176

[83] Castano AP Demidova TN Hamblin MR . Mechanisms in photodynamic therapy: part three—Photosensitizer pharmacokinetics, biodistribution, tumor localization and modes of tumor destruction. Photodiagnosis Photodyn Ther. (2005) 2:91–106. doi: 10.1016/S1572-1000(05)00060-8, PMID: 25048669 PMC4108218

[84] Kessel D Luo Y . Mitochondrial photodamage and Pdt-induced apoptosis. J Photochem Photobiol B. (1998) 42:89–95. doi: 10.1016/s1011-1344(97)00127-9, PMID: 9540214

[85] Oleinick NL Morris RL Belichenko I . The role of apoptosis in response to photodynamic therapy: what, where, why, and how. Photochem Photobiol Sci. (2002) 1:1–21. doi: 10.1039/b108586g, PMID: 12659143

[86] Li J Yuan J . Caspases in apoptosis and beyond. Oncogene. (2008) 27:6194–206. doi: 10.1038/onc.2008.297, PMID: 18931687

[87] Plaetzer K Kiesslich T Verwanger T Krammer B . The modes of cell death induced by Pdt: an overview. Med Laser Appl. (2003) 18:7–19. doi: 10.1078/1615-1615-00082, PMID: 37640969

[88] Mroz P Yaroslavsky A Kharkwal GB Hamblin MR . Cell death pathways in photodynamic therapy of cancer. Cancers. (2011) 3:2516–39 pp. doi: 10.3390/cancers3022516, PMID: 23914299 PMC3729395

[89] Zhou C . New trends in photobiology: mechanisms of tumor necrosis induced by photodynamic therapy. J Photochem Photobiol B: Biol. (1989) 3:299–318. doi: 10.1016/1011-1344(89)80035-1, PMID: 2504899

[90] Garg AD Agostinis P . Cell death and immunity in cancer: from danger signals to mimicry of pathogen defense responses. Immunol Rev. (2017) 280:126–48. doi: 10.1111/imr.12574, PMID: 29027218

[91] Wang W Moriyama LT Bagnato VS . Photodynamic therapy induced vascular damage: an overview of experimental Pdt. Laser Phys Lett. (2012) 10:023001. doi: 10.1088/1612-2011/10/2/023001

[92] Fingar VH . Vascular effects of photodynamic therapy. J Clin Laser Med Surg. (1996) 14:323–8. doi: 10.1089/clm.1996.14.323, PMID: 9612199

[93] Sidoroff A Thaler P . Taking treatment decisions in non-melanoma skin cancer--the place for topical photodynamic therapy (Pdt). Photodiagnosis Photodyn Ther. (2010) 7:24–32. doi: 10.1016/j.pdpdt.2009.12.004, PMID: 20230990

[94] Rook AH Wood GS Duvic M Vonderheid EC Tobia A Cabana B . A phase II placebo-controlled study of photodynamic therapy with topical hypericin and visible light irradiation in the treatment of cutaneous T-cell lymphoma and psoriasis. J Am Acad Dermatol. (2010) 63:984–90. doi: 10.1016/j.jaad.2010.02.039, PMID: 20889234

[95] Mosaddad SA Namanloo RA Aghili SS Maskani P Alam M Abbasi K . Photodynamic therapy in oral cancer: A review of clinical studies. Med Oncol. (2023) 40:91. doi: 10.1007/s12032-023-01949-3, PMID: 36749489

[96] Rigual NR Thankappan K Cooper M Sullivan MA Dougherty T Popat SR . Photodynamic therapy for head and neck dysplasia and cancer. Arch Otolaryngol Head Neck Surg. (2009) 135:784–8. doi: 10.1001/archoto.2009.98, PMID: 19687399 PMC2810853

[97] Jayadevappa R Chhatre S Soukiasian HJ Murgu S . Outcomes of patients with advanced non-small cell lung cancer and airway obstruction treated with photodynamic therapy and non-photodynamic therapy ablation modalities. J Thorac Dis. (2019) 11:4389–99. doi: 10.21037/jtd.2019.04.60, PMID: 31737325 PMC6837945

[98] Luketich JD Christie NA Buenaventura PO Weigel TL Keenan RJ Nguyen NT . Endoscopic photodynamic therapy for obstructing esophageal cancer: 77 cases over a 2-year period. Surg Endosc. (2000) 14:653–7. doi: 10.1007/s004640000144, PMID: 10948303

[99] Oinuma T Nakamura T Nishiwaki Y . Report on the national survey of photodynamic therapy (Pdt) for gastric cancer in Japan (a secondary publication). Laser Ther. (2016) 25:87–98. doi: 10.5978/islsm.16-OR-06, PMID: 27721560 PMC4961674

[100] Barr H Krasner N Boulos PB Chatlani P Bown SG . Photodynamic therapy for colorectal cancer: A quantitative pilot study. Br J Surg. (1990) 77:93–6. doi: 10.1002/bjs.1800770132, PMID: 2302524

[101] Stylli SS Kaye AH MacGregor L Howes M Rajendra P . Photodynamic therapy of high grade glioma - long term survival. J Clin Neurosci. (2005) 12:389–98. doi: 10.1016/j.jocn.2005.01.006, PMID: 15925768

[102] Muragaki Y Akimoto J Maruyama T Iseki H Ikuta S Nitta M . Phase II clinical study on intraoperative photodynamic therapy with talaporfin sodium and semiconductor laser in patients with Malignant brain tumors: clinical article. J Neurosurg. (2013) 119:845–52. doi: 10.3171/2013.7.JNS13415, PMID: 23952800

[103] Li H Long G Tian J . Efficacy and safety of photodynamic therapy for non-muscle-invasive bladder cancer: A systematic review and meta-analysis. Front Oncol. (2023) 13:1255632. doi: 10.3389/fonc.2023.1255632, PMID: 37860180 PMC10584312

[104] Rahman KMM Giram P Foster BA You Y . Photodynamic therapy for bladder cancers, a focused review. Photochem Photobiol. (2023) 99:420–36. doi: 10.1111/php.13726, PMID: 36138552 PMC10421568

[105] Taylor MN Gonzalez ML . The practicalities of photodynamic therapy in acne vulgaris. Br J Dermatol. (2009) 160:1140–8. doi: 10.1111/j.1365-2133.2009.09054.x, PMID: 19239465

[106] Choi YM Adelzadeh L Wu JJ . Photodynamic therapy for psoriasis. J Dermatol Treat. (2015) 26:202–7. doi: 10.3109/09546634.2014.927816, PMID: 24881473

[107] Stender IM Na R Fogh H Gluud C Wulf HC . Photodynamic therapy with 5-aminolaevulinic acid or placebo for recalcitrant foot and hand warts: randomised double-blind trial. Lancet. (2000) 355:963–6. doi: 10.1016/S0140-6736(00)90013-8, PMID: 10768434

[108] Wada I Shiose S Ishikawa K Kano K Notomi S Mori K . One-year efficacy of “Rescue photodynamic therapy” for patients with typical age-related macular degeneration, polypoidal choroidal vasculopathy, and pachychoroid neovasculopathy refractory to anti-vascular endothelial growth factor therapy. Graefe’s Arch Clin Exp Ophthalmol. (2022) 260:2029–36. doi: 10.1007/s00417-022-05553-5, PMID: 35038016

[109] Zhang R Gao T Wang D . Photodynamic therapy (Pdt) for oral leukoplakia: A systematic review and meta-analysis of single-arm studies examining efficacy and subgroup analyses. BMC Oral Health. (2023) 23:568. doi: 10.1186/s12903-023-03294-3, PMID: 37574560 PMC10424357

[110] Piccolino FC Eandi CM Ventre L Rigault de la Longrais RC Grignolo FM . Photodynamic therapy for chronic central serous chorioretinopathy. Retina. (2003) 23(6):752–63. doi: 10.1097/00006982-200312000-00002, PMID: 14707823

[111] van Dijk EHC Fauser S Breukink MB Blanco-Garavito R Groenewoud JMM Keunen JEE . Half-dose photodynamic therapy versus high-density subthreshold micropulse laser treatment in patients with chronic central serous chorioretinopathy: the place trial. Ophthalmology. (2018) 125:1547–55. doi: 10.1016/j.ophtha.2018.04.021, PMID: 29776672

[112] Elsadek MF Farahat MF . Effectiveness of photodynamic therapy as an adjunct to periodontal scaling for treating periodontitis in geriatric patients. Eur Rev Med Pharmacol Sci. (2022) 26:1832–8. doi: 10.26355/eurrev_202203_28327, PMID: 35363330

[113] Dorst DN Rijpkema M Buitinga M Walgreen B Helsen MMA Brennan E . Targeting of fibroblast activation protein in rheumatoid arthritis patients: imaging and ex vivo photodynamic therapy. Rheumatol (Oxford). (2022) 61:2999–3009. doi: 10.1093/rheumatology/keab664, PMID: 34450633 PMC9258553

[114] Rockson SG Kramer P Razavi M Szuba A Filardo S Fitzgerald P . Photoangioplasty for Human Peripheral Atherosclerosis : Results of a Phase I Trial of Photodynamic Therapy With Motexafin Lutetium (Antrin). Circulation. (2000) 102:2322–4. doi: 10.1161/01.CIR.102.19.2322, PMID: 11067782

[115] Lee BI Lee S Suh YS Lee JS Kim A-k Kwon OY . Photoexcited porphyrins as a strong suppressor of B-amyloid aggregation and synaptic toxicity. Angewandte Chemie Int Edition. (2015) 54:11472–6. doi: 10.1002/anie.201504310, PMID: 26178411

[116] Yan C Wang C Shao X Teng Y Chen P Hu X . Multifunctional carbon-dot-photosensitizer nanoassemblies for inhibiting amyloid aggregates, suppressing microbial infection, and overcoming the blood–brain barrier. ACS Appl Materials Interfaces. (2022) 14:47432–44. doi: 10.1021/acsami.2c14118, PMID: 36254877

[117] Choi MC Jung SG Park H Lee SY Lee C Hwang YY . Fertility preservation by photodynamic therapy combined with conization in young patients with early stage cervical cancer: A pilot study. Photodiagnosis Photodyn Ther. (2014) 11:420–5. doi: 10.1016/j.pdpdt.2014.06.001, PMID: 24927981

[118] Choi MC Kim MS Lee GH Jung SG Park H Joo WD . Photodynamic therapy for premalignant lesions of the vulva and vagina: A long-term follow-up study. Lasers Surg Med. (2015) 47:566–70. doi: 10.1002/lsm.22384, PMID: 26174756

[119] Han Q Guo H Wu Z Shi J Zhang X . Efficacy and safety of 5-aminolevulinic acid photodynamic therapy for treating cervical and vaginal intraepithelial neoplasia. Pharmaceutics. (2024) 16(5):627. doi: 10.3390/pharmaceutics16050627, PMID: 38794289 PMC11126115

[120] Hillemanns P Garcia F Petry KU Dvorak V Sadovsky O Iversen OE . A randomized study of hexaminolevulinate photodynamic therapy in patients with cervical intraepithelial neoplasia 1/2. Am J Obstet Gynecol. (2015) 212:465.e1–7. doi: 10.1016/j.ajog.2014.10.1107, PMID: 25467012

[121] Hillemanns P Untch M Dannecker C Baumgartner R Stepp H Diebold J . Photodynamic therapy of vulvar intraepithelial neoplasia using 5-aminolevulinic acid. Int J Cancer. (2000) 85:649–53. doi: 10.1002/(sici)1097-0215(20000301)85:5<649::Aid-ijc9>3.0.Co;2-e 10699944

[122] Zhang W Zhang A Sun W Yue Y Li H . Efficacy and safety of photodynamic therapy for cervical intraepithelial neoplasia and human papilloma virus infection: A systematic review and meta-analysis of randomized clinical trials. Med (Baltimore). (2018) 97:e10864. doi: 10.1097/MD.0000000000010864, PMID: 29794788 PMC6392907

[123] Zhou M Su Y Tong Y Zhang C Yuan S Zhang M . Comparative study of topical 5-aminolevulinic acid photodynamic therapy and surgery for the treatment of vulvar squamous intraepithelial lesion. Photodiagnosis Photodyn Ther. (2023) 44:103868. doi: 10.1016/j.pdpdt.2023.103868, PMID: 37898260

[124] Choi MC Jung SG Park H Cho YH Lee C Kim SJ . Fertility preservation via photodynamic therapy in young patients with early-stage uterine endometrial cancer: A long-term follow-up study. Int J Gynecol Cancer. (2013) 23:698–704. doi: 10.1097/IGC.0b013e31828b5ba2, PMID: 23478222

[125] Naidu G Zuva T Sibanda EM . A review of evaluation metrics in machine learning algorithms. In: Silhavy R Silhavy P , editors. Artificial Intelligence Application in Networks and Systems. Cham: Springer International Publishing (2023). p. 15–25. doi: 10.1007/978-3-031-35314-7_2, PMID:

[126] Schneider A Hommel G Blettner M . Linear regression analysis. Dtsch Arztebl Int. (2010) 107:776–82. doi: 10.3238/arztebl.2010.0776, PMID: 21116397 PMC2992018

[127] LaValley MP . Logistic regression. Circulation. (2008) 117:2395–9. doi: 10.1161/CIRCULATIONAHA.106.682658, PMID: 18458181

[128] Rudin C . Stop explaining black box machine learning models for high stakes decisions and use interpretable models instead. Nat Mach Intell. (2019) 1:206–15. doi: 10.1038/s42256-019-0048-x, PMID: 35603010 PMC9122117

[129] Bentéjac C Csörgő A Martínez-Muñoz G . A comparative analysis of gradient boosting algorithms. Artif Intell Rev. (2021) 54:1937–67. doi: 10.1007/s10462-020-09896-5, PMID: 41865474

[130] Pisner DA Schnyer DM . Support vector machine. In: Mechelli A Vieira S , editors. Machine Learning. Academic Press (2020). p. 101–21.

[131] Peterson LE . K-Nearest Neighbor. Scholarpedia. (2009) 4:1883. doi: 10.4249/scholarpedia.1883

[132] Xu R Wunsch D 2nd . Survey of clustering algorithms. IEEE Trans Neural Netw. (2005) 16:645–78. doi: 10.1109/TNN.2005.845141, PMID: 15940994

[133] Kaur R Singh S . A comprehensive review of object detection with deep learning. Digital Signal Process. (2023) 132:103812. doi: 10.1016/j.dsp.2022.103812, PMID: 41865745

[134] Mehrish A Majumder N Bharadwaj R Mihalcea R Poria S . A review of deep learning techniques for speech processing. Inf Fusion. (2023) 99:101869. doi: 10.1016/j.inffus.2023.101869, PMID: 41865745

[135] Mohammed A Kora R . A comprehensive review on ensemble deep learning: opportunities and challenges. J King Saud Univ Comput Inf Sci. (2023) 35:757–74. doi: 10.1016/j.jksuci.2023.01.014, PMID: 41865745

[136] Soori M Arezoo B Dastres R . Artificial intelligence, machine learning and deep learning in advanced robotics, a review. Cogn Robotics. (2023) 3:54–70. doi: 10.1016/j.cogr.2023.04.001, PMID: 41865745

[137] LeCun Y Bengio Y Hinton G . Deep learning. Nature. (2015) 521:436–44. doi: 10.1038/nature14539, PMID: 26017442

[138] Kriegeskorte N Golan T . Neural network models and deep learning. Curr Biol. (2019) 29:R231–R6. doi: 10.1016/j.cub.2019.02.034, PMID: 30939301

[139] Wythoff BJ . Backpropagation neural networks - a tutorial. Chemometrics Intelligent Lab Syst. (1993) 18:115–55. doi: 10.1016/0169-7439(93)80052-J

[140] Gu JX Wang ZH Kuen J Ma LY Shahroudy A Shuai B . Recent advances in convolutional neural networks. Pattern Recognition. (2018) 77:354–77. doi: 10.1016/j.patcog.2017.10.013, PMID: 41865745

[141] Salehinejad H Sankar S Barfett J Colak E Valaee S . Recent advances in recurrent neural networks. arXiv preprint arXiv:180101078. (2018). doi: 10.48550/arXiv.1801.01078, PMID: 41363103

[142] Yu Y Si X Hu C Zhang J . A review of recurrent neural networks: Lstm cells and network architectures. Neural Comput. (2019) 31:1235–70. doi: 10.1162/neco_a_01199, PMID: 31113301

[143] Goodfellow I Pouget-Abadie J Mirza M Xu B Warde-Farley D Ozair S . Generative adversarial networks. Commun ACM. (2020) 63:139–44. doi: 10.1145/3422622, PMID: 40727313

[144] Ho J Jain A Abbeel P . Denoising Diffusion Probabilistic Models. In: Proceedings of the 34th International Conference on Neural Information Processing Systems. Vancouver, BC, Canada: Curran Associates Inc. (2020) p. 6840–6851. Article 574. Available online at: https://dl.acm.org/doi/abs/10.5555/3495724.3496298

[145] Yassine AA Lilge L Betz V . Machine learning for real-time optical property recovery in interstitial photodynamic therapy: A stimulation-based study. BioMed Opt Express. (2021) 12:5401–22. doi: 10.1364/BOE.431310, PMID: 34692191 PMC8515975

[146] Xie H Xie Z Mousavi M Bendsoe N Brydegaard M Axelsson J . Design and validation of a fiber optic point probe instrument for therapy guidance and monitoring. J BioMed Opt. (2014) 19:71408. doi: 10.1117/1.JBO.19.7.071408, PMID: 24623193

[147] Chong KC Pramanik M . Physics-guided neural network for tissue optical properties estimation. BioMed Opt Express. (2023) 14:2576–90. doi: 10.1364/BOE.487179, PMID: 37342718 PMC10278626

[148] Hannan MN Baran TM . Application of transfer learning for rapid calibration of spatially resolved diffuse reflectance probes for extraction of tissue optical properties. J BioMed Opt. (2024) 29:27004. doi: 10.1117/1.JBO.29.2.027004, PMID: 38419753 PMC10901350

[149] Buglak AA Charisiadis A Sheehan A Kingsbury CJ Senge MO Filatov MA . Quantitative structure-property relationship modelling for the prediction of singlet oxygen generation by heavy-atom-free bodipy photosensitizers*. Chemistry. (2021) 27:9934–47. doi: 10.1002/chem.202100922, PMID: 33876842 PMC8362084

[150] Buglak AA Filatov MA Hussain MA Sugimoto M . Singlet oxygen generation by porphyrins and metalloporphyrins revisited: A quantitative structure-property relationship (Qspr) study. J Photochem Photobiol A: Chem. (2020) 403:112833. doi: 10.1016/j.jphotochem.2020.112833, PMID: 41865745

[151] Buglak AA Chebotaev PP Filatov MA . Applications of Qspr and machine learning in molecular photonics. Advanced Optical Materials. (2025) 13(33):e01713. doi: 10.1002/adom.202501713, PMID: 41859965

[152] He LQ Dong JP Yang YH Huang ZH Ye SP Ke XT . Accelerating the discovery of type II photosensitizer: experimentally validated machine learning models for predicting the singlet oxygen quantum yield of photosensitive molecule. J Mol Structure. (2025) 1321:119118. doi: 10.1016/j.molstruc.2024.139850, PMID: 41865745

[153] Gao J Dong Y Qiu T Sun W Du J . Dft-ml-based property prediction of transition metal complex photosensitizers for photodynamic therapy. ACS Omega. (2025) 10:53447–59. doi: 10.1021/acsomega.5c08727, PMID: 41244434 PMC12613122

[154] Rittig JG Gao Q Dahmen M Mitsos A Schweidtmann AM . Graph neural networks for the prediction of molecular structure-property relationships. In: Zhang D del Río Chanona EA editors. Machine Learning and Hybrid Modelling for Reaction Engineering: Theory and Applications. 26. Royal Society of Chemistry (2023). p. 159–81. doi: 10.1039/BK9781837670178-00159, PMID: 41859911

[155] Joung JF Han M Hwang J Jeong M Choi DH Park S . Deep learning optical spectroscopy based on experimental database: potential applications to molecular design. JACS Au. (2021) 1:427–38. doi: 10.1021/jacsau.1c00035, PMID: 34467305 PMC8395663

[156] Nguyen DP Le QM Tran HTP Le PT Nguyen TVT . Enhanced prediction of absorption and emission wavelengths of organic compounds through hybrid graph neural network architectures. ACS Omega. (2025) 10:50643–51. doi: 10.1021/acsomega.5c08722, PMID: 41179196 PMC12573182

[157] Wang H Zheng X Liu W Tang Z Gong S . Artificial intelligence driven workflow for accelerating design of novel photosensitizers. AI for Science. (2026). doi: 10.1088/3050-287X/ae4412, PMID: 41725453

[158] Tang X Dai H Knight E Wu F Li Y Li T . A survey of generative Ai for de novo drug design: new frontiers in molecule and protein generation. Brief Bioinform. (2024) 25:bbae338. doi: 10.1093/bib/bbae338, PMID: 39007594 PMC11247410

[159] Popova M Isayev O Tropsha A . Deep reinforcement learning for de novo drug design. Sci Adv. (2018) 4:eaap7885. doi: 10.1126/sciadv.aap7885, PMID: 30050984 PMC6059760

[160] Zhou Z Kearnes S Li L Zare RN Riley P . Optimization of molecules via deep reinforcement learning. Sci Rep. (2019) 9:10752. doi: 10.1038/s41598-019-47148-x, PMID: 31341196 PMC6656766

[161] Alakhdar A Poczos B Washburn N . Diffusion models in *de novo* drug design. J Chem Inf Model. (2024) 64:7238–56. doi: 10.1021/acs.jcim.4c01107, PMID: 39322943 PMC11481093

[162] Chen W Mao X-Q Wang X-Z Liao Y-C Yin X-Y Wu H-L . Data-driven multi-stage screening for discovering type I photosensitizers with nir absorption for Rna-targeted tumor Pdt. Chem Sci. (2025) 16:14455–67. doi: 10.1039/D5SC03648H, PMID: 40698170 PMC12278499

[163] Chen K Zhang X Wang J Li D Hou T Yang W . Effective generation of heavy-atom-free triplet photosensitizers containing multiple intersystem crossing mechanisms based on deep learning. Chem Sci. (2025) 16:14698–709. doi: 10.1039/D5SC03192C, PMID: 40671753 PMC12261949

[164] Schmidt F Wenzel J Halland N Gussregen S Delafoy L Czich A . Computational investigation of drug phototoxicity: photosafety assessment, photo-toxophore identification, and machine learning. Chem Res Toxicol. (2019) 32:2338–52. doi: 10.1021/acs.chemrestox.9b00338, PMID: 31625387

[165] Igarashi Y Re S Kojima R Okuno Y Yamada H . Development of a gcn-based model to predict *in vitro* phototoxicity from the chemical structure and homo-lumo gap. J Toxicol Sci. (2023) 48:243–9. doi: 10.2131/jts.48.243, PMID: 37121739

[166] Sharma B Chenthamarakshan V Dhurandhar A Pereira S Hendler JA Dordick JS . Accurate clinical toxicity prediction using multi-task deep neural nets and contrastive molecular explanations. Sci Rep. (2023) 13:4908. doi: 10.1038/s41598-023-31169-8, PMID: 36966203 PMC10039880

[167] Dodds M Guo J Löhr T Tibo A Engkvist O Janet JP . Sample efficient reinforcement learning with active learning for molecular design. Chem Sci. (2024) 15:4146–60. doi: 10.1039/D3SC04653B, PMID: 38487235 PMC10935729

[168] Ban Z Yuan P Yu F Peng T Zhou Q Hu X . Machine learning predicts the functional composition of the protein corona and the cellular recognition of nanoparticles. Proc Natl Acad Sci U.S.A. (2020) 117:10492–9. doi: 10.1073/pnas.1919755117, PMID: 32332167 PMC7229677

[169] Fu X Yang C Su Y Liu C Qiu H Yu Y . Machine learning enables comprehensive prediction of the relative protein abundance of multiple proteins on the protein corona. Res (Wash D C). (2024) 7:487. doi: 10.34133/research.0487, PMID: 39324017 PMC11423712

[170] Alafeef M Srivastava I Pan D . Machine learning for precision breast cancer diagnosis and prediction of the nanoparticle cellular internalization. ACS Sens. (2020) 5:1689–98. doi: 10.1021/acssensors.0c00329, PMID: 32466640

[171] Chou WC Chen Q Yuan L Cheng YH He C Monteiro-Riviere NA . An artificial intelligence-assisted physiologically-based pharmacokinetic model to predict nanoparticle delivery to tumors in mice. J Control Release. (2023) 361:53–63. doi: 10.1016/j.jconrel.2023.07.040, PMID: 37499908 PMC11008607

[172] Li B Raji IO Gordon AGR Sun L Raimondo TM Oladimeji FA . Accelerating ionizable lipid discovery for mrna delivery using machine learning and combinatorial chemistry. Nat Mater. (2024) 23:1002–8. doi: 10.1038/s41563-024-01867-3, PMID: 38740955

[173] Xu X Shen Y Lin L Lin L Li B . Multi-step deep neural network for identifying subfascial vessels in a dorsal skinfold window chamber model. BioMed Opt Express. (2022) 13:426–37. doi: 10.1364/BOE.446214, PMID: 35154882 PMC8803012

[174] Varon E Blumrosen G Shefi O . A predictive model for personalization of nanotechnology-based phototherapy in cancer treatment. Front Oncol. (2022) 12:1037419. doi: 10.3389/fonc.2022.1037419, PMID: 36911792 PMC9999042

[175] Rahman MA Yan F Li R Wang Y Huang L Han R . Deep learning insights into the dynamic effects of photodynamic therapy on cancer cells. Pharmaceutics. (2024) 16:673. doi: 10.3390/pharmaceutics16050673, PMID: 38794335 PMC11125085

[176] Yoo TK Kim SH Kim M Lee CS Byeon SH Kim SS . Deeppdt-net: predicting the outcome of photodynamic therapy for chronic central serous chorioretinopathy using two-stage multimodal transfer learning. Sci Rep. (2022) 12:18689. doi: 10.1038/s41598-022-22984-6, PMID: 36333442 PMC9636239

[177] Zuhayri H Samarinova AA Borisov AV Guardado DAL Baalbaki H Krivova NA . Quantitative assessment of low-dose photodynamic therapy effects on diabetic wound healing using raman spectroscopy. Pharmaceutics. (2023) 15:595. doi: 10.3390/pharmaceutics15020595, PMID: 36839917 PMC9966264

[178] Hara C Maruyama K Wakabayashi T Liu S Mao Z Kawasaki R . Choroidal vessel and stromal volumetric analysis after photodynamic therapy or focal laser for central serous chorioretinopathy. Transl Vis Sci Technol. (2023) 12:26. doi: 10.1167/tvst.12.11.26, PMID: 37982766 PMC10668616

[179] Wang F Song Y Xu H Liu J Tang F Yang D . Prediction of the short-term efficacy and recurrence of photodynamic therapy in the treatment of oral leukoplakia based on deep learning. Photodiagnosis Photodyn Ther. (2024) 48:104236. doi: 10.1016/j.pdpdt.2024.104236, PMID: 38851310

[180] Fernandez-Vigo JI Gomez Calleja V de Moura Ramos JJ Novo-Bujan J Burgos-Blasco B Lopez-Guajardo L . Prediction of the response to photodynamic therapy in patients with chronic central serous chorioretinopathy based on optical coherence tomography using deep learning. Photodiagnosis Photodyn Ther. (2022) 40:103107. doi: 10.1016/j.pdpdt.2022.103107, PMID: 36070850

[181] Mitchell MJ Billingsley MM Haley RM Wechsler ME Peppas NA Langer R . Engineering precision nanoparticles for drug delivery. Nat Rev Drug Discov. (2021) 20:101–24. doi: 10.1038/s41573-020-0090-8, PMID: 33277608 PMC7717100

[182] Gavas S Quazi S Karpinski TM . Nanoparticles for cancer therapy: current progress and challenges. Nanoscale Res Lett. (2021) 16:173. doi: 10.1186/s11671-021-03628-6, PMID: 34866166 PMC8645667

[183] Fang RH Gao W Zhang L . Targeting drugs to tumours using cell membrane-coated nanoparticles. Nat Rev Clin Oncol. (2023) 20:33–48. doi: 10.1038/s41571-022-00699-x, PMID: 36307534

[184] Zhou Y Zou P Chen X Chen P Shi M Lang J . Overcoming barriers in photodynamic therapy harnessing nanogenerators strategies. Int J Biol Sci. (2024) 20:5673–94. doi: 10.7150/ijbs.100317, PMID: 39494340 PMC11528466

[185] Rao L Yuan Y Shen X Yu G Chen X . Designing nanotheranostics with machine learning. Nat Nanotechnology. (2024) 19:1769–81. doi: 10.1038/s41565-024-01753-8, PMID: 39362960

[186] Hamilton S Kingston BR . Applying artificial intelligence and computational modeling to nanomedicine. Curr Opin Biotechnol. (2024) 85:103043. doi: 10.1016/j.copbio.2023.103043, PMID: 38091874

[187] Tao HC Wu TY Aldeghi M Wu TC Aspuru-Guzik A Kumacheva E . Nanoparticle synthesis assisted by machine learning. Nat Rev Materials. (2021) 6:701–16. doi: 10.1038/s41578-021-00337-5, PMID: 41862587

[188] Xue L Hamilton AG Zhao G Xiao Z El-Mayta R Han X . High-throughput barcoding of nanoparticles identifies cationic, degradable lipid-like materials for Mrna delivery to the lungs in female preclinical models. Nat Commun. (2024) 15:1884. doi: 10.1038/s41467-024-45422-9, PMID: 38424061 PMC10904786

[189] Chaurawal N Quadir SS Joshi G Barkat MA Alanezi AA Raza K . Development of fucoidan/polyethyleneimine based sorafenib-loaded self-assembled nanoparticles with machine learning and doe-ann implementation: optimization, characterization, and in-vitro assessment for the anticancer drug delivery. Int J Biol Macromol. (2024) 279:135123. doi: 10.1016/j.ijbiomac.2024.135123, PMID: 39208886

[190] Debnath G Vasu B Gorla RSR . Current state-of-the-art in multi-scale modeling in nano-cancer drug delivery: role of Ai and machine learning. Cancer Nanotechnology. (2025) 16:45. doi: 10.1186/s12645-025-00326-1, PMID: 41863001

[191] Mahmoudi M Landry MP Moore A Coreas R . The protein corona from nanomedicine to environmental science. Nat Rev Mater. (2023) 8:1–17. doi: 10.1038/s41578-023-00552-2, PMID: 37361608 PMC10037407

[192] Canchola A Li KY Chen KP Borboa-Pimentel A Chou C Dela Rama R . Meta-analysis and machine learning prediction of protein corona composition across nanoparticle systems in biological media. ACS Nano. (2025) 19:37633–50. doi: 10.1021/acsnano.5c08608, PMID: 41031442 PMC12593346

[193] Mi K Chou WC Chen Q Yuan L Kamineni VN Kuchimanchi Y . Predicting tissue distribution and tumor delivery of nanoparticles in mice using machine learning models. J Control Release. (2024) 374:219–29. doi: 10.1016/j.jconrel.2024.08.015, PMID: 39146980 PMC11886896

[194] Kapoor DU Sharma JB Gandhi SM Prajapati BG Thanawuth K Limmatvapirat S . Ai-driven design and optimization of nanoparticle-based drug delivery systems. Science Eng Health Stud. (2024) 18:24010003. doi: 10.69598/sehs.18.24010003

[195] Sun B Bte Rahmat JN Zhang Y . Advanced techniques for performing photodynamic therapy in deep-seated tissues. Biomaterials. (2022) 291:121875. doi: 10.1016/j.biomaterials.2022.121875, PMID: 36335717

[196] Chen G Qiu H Prasad PN Chen X . Upconversion nanoparticles: design, nanochemistry, and applications in theranostics. Chem Rev. (2014) 114:5161–214. doi: 10.1021/cr400425h, PMID: 24605868 PMC4039352

[197] Chota A Abrahamse H George BP . Nano-enhanced photodynamic therapy and machine learning: advancements, challenges, and future directions. BioMed Pharmacother. (2025) 193:118710. doi: 10.1016/j.biopha.2025.118710, PMID: 41191983

[198] Bhujel R Enkmann V Burgstaller H Maharjan R . Artificial intelligence-driven strategies for targeted delivery and enhanced stability of Rna-based lipid nanoparticle cancer vaccines. Pharmaceutics. (2025) 17:992. doi: 10.3390/pharmaceutics17080992, PMID: 40871015 PMC12389219

[199] Shan X Cai Y Zhu B Zhou L Sun X Xu X . Rational Strategies for Improving the Efficiency of Design and Discovery of Nanomedicines. Nature Communications. (2024) 15:9990. doi: 10.1038/s41467-024-54265-3, PMID: 39557860 PMC11574076

[200] Chou W-C Canchola A Zhang F Lin Z . Machine Learning and Artificial Intelligence in Nanomedicine. WIREs Nanomedicine and Nanobiotechnology. (2025) 17:e70027. doi: 10.1002/wnan.70027, PMID: 40813104 PMC12353477

[201] Li Y El Habib Daho M Conze P-H Zeghlache R Le Boité H Tadayoni R . A review of deep learning-based information fusion techniques for multimodal medical image classification. Comput Biol Med. (2024) 177:108635. doi: 10.1016/j.compbiomed.2024.108635, PMID: 38796881

[202] Sheller MJ Edwards B Reina GA Martin J Pati S Kotrotsou A . Federated learning in medicine: facilitating multi-institutional collaborations without sharing patient data. Sci Rep. (2020) 10:12598. doi: 10.1038/s41598-020-69250-1, PMID: 32724046 PMC7387485

[203] Yahiaoui ME Derdour M Abdulghafor R Turaev S Gasmi M Bennour A . Federated learning with privacy preserving for multi- institutional three-dimensional brain tumor segmentation. Diagnostics. (2024) 14:2891 p. doi: 10.3390/diagnostics14242891, PMID: 39767253 PMC11675895

[204] Rieke N Hancox J Li W Milletarì F Roth HR Albarqouni S . The future of digital health with federated learning. NPJ Digital Med. (2020) 3:119. doi: 10.1038/s41746-020-00323-1, PMID: 33015372 PMC7490367

[205] Li C Guo Y Lin X Feng X Xu D Yang R . Deep reinforcement learning in radiation therapy planning optimization: A comprehensive review. Physica Medica: Eur J Med Phys. (2024) 125:104498. doi: 10.1016/j.ejmp.2024.104498, PMID: 39163802

[206] Tseng H-H Luo Y Cui S Chien J-T Ten Haken RK Naqa IE . Deep reinforcement learning for automated radiation adaptation in lung cancer. Med Phys. (2017) 44:6690–705. doi: 10.1002/mp.12625, PMID: 29034482 PMC5734677

[207] Hrinivich WT Bhattacharya M Mekki L McNutt T Jia X Li H . Clinical vmat machine parameter optimization for localized prostate cancer using deep reinforcement learning. Med Phys. (2024) 51:3972–84. doi: 10.1002/mp.17100, PMID: 38669457

[208] Xiang H Zeng L Hou L Li K Fu Z Qiu Y . A molecular video-derived foundation model for scientific drug discovery. Nat Commun. (2024) 15:9696. doi: 10.1038/s41467-024-53742-z, PMID: 39516468 PMC11549228

[209] Méndez-Lucio O Nicolaou CA Earnshaw B . Mole: A foundation model for molecular graphs using disentangled attention. Nat Commun. (2024) 15:9431. doi: 10.1038/s41467-024-53751-y, PMID: 39532853 PMC11557931

[210] Pyzer-Knapp EO Manica M Staar P Morin L Ruch P Laino T . Foundation models for materials discovery – current state and future directions. NPJ Comput Materials. (2025) 11:61. doi: 10.1038/s41524-025-01538-0, PMID: 41862587

[211] Tom G Schmid SP Baird SG Cao Y Darvish K Hao H . Self-driving laboratories for chemistry and materials science. Chem Rev. (2024) 124:9633–732. doi: 10.1021/acs.chemrev.4c00055, PMID: 39137296 PMC11363023

[212] Abolhasani M Kumacheva E . The rise of self-driving labs in chemical and materials sciences. Nat Synthesis. (2023) 2:483–92. doi: 10.1038/s44160-022-00231-0, PMID: 41862587

[213] Tobias AV Wahab A . Autonomous ‘Self-driving’ Laboratories: A review of technology and policy implications. R Soc Open Sci. (2025) 12:250646. doi: 10.1098/rsos.250646, PMID: 40852582 PMC12368842

[214] Abbas Q Jeong W Lee SW . Explainable Ai in clinical decision support systems: A meta-analysis of methods, applications, and usability challenges. Healthcare. (2025) 13:2154 p. doi: 10.3390/healthcare13172154, PMID: 40941506 PMC12427955

[215] Noor AA Manzoor A Mazhar Qureshi MD Qureshi MA Rashwan W . Unveiling explainable Ai in healthcare: current trends, challenges, and future directions. WIREs Data Min Knowledge Discov. (2025) 15:e70018. doi: 10.1002/widm.70018, PMID: 41859965

[216] Elhaddad M Hamam S . Ai-driven clinical decision support systems: an ongoing pursuit of potential. Cureus. (2024) 16:e57728. doi: 10.7759/cureus.57728, PMID: 38711724 PMC11073764

[217] Liu S McCoy AB Peterson JF Lasko TA Sittig DF Nelson SD . Leveraging explainable artificial intelligence to optimize clinical decision support. J Am Med Inf Assoc. (2024) 31:968–74. doi: 10.1093/jamia/ocae019, PMID: 38383050 PMC10990514

[218] Singh R Bapna M Diab AR Ruiz ES Lotter W . How Ai is used in Fda-authorized medical devices: A taxonomy across 1,016 authorizations. NPJ Digital Med. (2025) 8:388. doi: 10.1038/s41746-025-01800-1, PMID: 40596700 PMC12219150

[219] Sandalow M Adams K Loud G . FDA Oversight: Understanding the Regulation of Health Ai Tools. Washington, DC: Bipartisan Policy Center (2025). Available online at: https://bipartisanpolicy.org/issue-brief/fda-oversight-understanding-the-regulation-of-health-ai-tools/.

[220] Wilson BC Lilge L Weersink RA Pires L . Photodynamic therapy dosimetry: current status and the emerging challenge of immune stimulation. J BioMed Opt. (2025) 30:S34118. doi: 10.1117/1.JBO.30.S3.S34118, PMID: 41425296 PMC12715725

[221] Center MIaDR . Medical Imaging and Data Resource Center (Midrc) (2024). Available online at: https://www.midrc.org (Accessed January 21, 2025).

[222] Collins GS Moons KGM Dhiman P Riley RD Beam AL Van Calster B . Tripod+Ai statement: updated guidance for reporting clinical prediction models that use regression or machine learning methods. BMJ. (2024) 385:e078378. doi: 10.1136/bmj-2023-078378, PMID: 38626948 PMC11019967

[223] Abràmoff MD Lavin PT Birch M Shah N Folk JC . Pivotal trial of an autonomous Ai-based diagnostic system for detection of diabetic retinopathy in primary care offices. NPJ Digital Med. (2018) 1:39. doi: 10.1038/s41746-018-0040-6, PMID: 31304320 PMC6550188

[224] Abramoff MD Roehrenbeck C Trujillo S Goldstein J Graves AS Repka MX . A reimbursement framework for artificial intelligence in healthcare. NPJ Digit Med. (2022) 5:72. doi: 10.1038/s41746-022-00621-w, PMID: 35681002 PMC9184542

[225] Advanced Research Projects Agency for Health (ARPA-H) . PRECISE-AI: Performance and Reliability Evaluation for Continuous Modifications and Useability of Artificial Intelligence [Internet] (2024). Available online at: https://arpa-h.gov/explore-funding/programs/precise-ai (Accessed January 21, 2025).

[226] Zou J Huss M Abid A Mohammadi P Torkamani A Telenti A . A primer on deep learning in genomics. Nat Genet. (2019) 51:12–8. doi: 10.1038/s41588-018-0295-5, PMID: 30478442 PMC11180539

[227] Libbrecht MW Noble WS . Machine learning applications in genetics and genomics. Nat Rev Genet. (2015) 16:321–32. doi: 10.1038/nrg3920, PMID: 25948244 PMC5204302

[228] Wang J Wang S Zhang Y . Deep learning on medical image analysis. CAAI Trans Intell Technol. (2025) 10:1–35. doi: 10.1049/cit2.12356, PMID: 40688697

[229] Topol EJ . High-performance medicine: the convergence of human and artificial intelligence. Nat Med. (2019) 25:44–56. doi: 10.1038/s41591-018-0300-7, PMID: 30617339

[230] Zhang R Pei C Shi J Wang S . Construction and validation of a general medical image dataset for pretraining. J Imaging Inform Med. (2025) 38:1051–61. doi: 10.1007/s10278-024-01226-3, PMID: 39147887 PMC11950592

[231] Gao C Li M . Deep transfer learning using real-world image features for medical image classification. Bioengineering. (2024) 11:406. doi: 10.3390/bioengineering11040406, PMID: 38671827 PMC11048359

[232] Moen E Bannon D Kudo T Graf W Covert M Van Valen D . Deep learning for cellular image analysis. Nat Methods. (2019) 16:1233–46. doi: 10.1038/s41592-019-0403-1, PMID: 31133758 PMC8759575

[233] Eraslan G Simon LM Mircea M Mueller NS Theis FJ . Single-cell rna-seq denoising using a deep count autoencoder. Nat Commun. (2019) 10:390. doi: 10.1038/s41467-018-07931-2, PMID: 30674886 PMC6344535

[234] Haripriya R Khare N Pandey M . Privacy-preserving federated learning for collaborative medical data mining in multi-institutional settings. Sci Rep. (2025) 15:12482. doi: 10.1038/s41598-025-97565-4, PMID: 40217112 PMC11992079

[235] Alkhanbouli R Matar Abdulla Almadhaani H Alhosani F Simsekler MCE . The role of explainable artificial intelligence in disease prediction: A systematic literature review and future research directions. BMC Med Inform Decis Mak. (2025) 25:110. doi: 10.1186/s12911-025-02944-6, PMID: 40038704 PMC11877768

[236] Marey A Arjmand P Alerab ADS Eslami MJ Saad AM Sanchez N . Explainability, transparency and black box challenges of AI in radiology: impact on patient care in cardiovascular radiology. Egyptian J Radiol Nucl Med. (2024) 55:183. doi: 10.1186/s43055-024-01356-2, PMID: 41863001

[237] Blackman J Veerapen R . On the practical, ethical, and legal necessity of clinical artificial intelligence explainability: an examination of key arguments. BMC Med Inform Decis Mak. (2025) 25:111. doi: 10.1186/s12911-025-02891-2, PMID: 40045339 PMC11881432

[238] Mienye ID Obaido G Jere N Mienye E Aruleba K Emmanuel ID . A survey of explainable artificial intelligence in healthcare: concepts, applications, and challenges. Inf Med Unlocked. (2024) 51:101587. doi: 10.1016/j.imu.2024.101587, PMID: 41865745

[239] Selvaraju RR Cogswell M Das A Vedantam R Parikh D Batra D . Grad-cam: visual explanations from deep networks via gradient-based localization. Int J Comput Vision. (2020) 128:336–59. doi: 10.1007/s11263-019-01228-7, PMID: 41865474

[240] Lundberg SM Lee SI . A unified approach to interpreting model predictions. Proceedings of the 31st International Conference on Neural Information Processing Systems. Long Beach, California, USA: Curran Associates Inc (2017) 4768–77. Available online at: https://dl.acm.org/doi/10.5555/3295222.3295230

[241] Adebayo J Gilmer J Muelly M Goodfellow I Hardt M Kim B . Sanity checks for saliency maps. Proceedings of the 32nd International Conference on Neural Information Processing Systems. Montréal, Canada: Curran Associates Inc. (2018) p. 9525–36. Available online at: https://dl.acm.org/doi/10.5555/3327546.3327621

[242] Cai CJ Reif E Hegde N Hipp J Kim B Smilkov D . Human-centered tools for coping with imperfect algorithms during medical decision-making. Proceedings of the 2019 CHI Conference on Human Factors in Computing Systems. Glasgow, Scotland Uk: Association for Computing Machinery (2019) p. Paper 4.

[243] Tonekaboni S Joshi S McCradden MD Goldenberg A . What clinicians want: contextualizing explainable machine learning for clinical end use. In: Finale D-V Jim F Ken J David K Rajesh R Byron W , editors. Proceedings of the 4th Machine Learning for Healthcare Conference. Proceedings of Machine Learning Research: PMLR (2019). p. 359–80.

[244] Serra-Burriel M Locher L Vokinger KN . Development pipeline and geographic representation of trials for Ai/Ml-enabled medical devices (2010 to 2023). NEJM AI. (2024) 1:AIp2300038. doi: 10.1056/AIpc2300038, PMID: 41686241

[245] Kelly CJ Karthikesalingam A Suleyman M Corrado G King D . Key challenges for delivering clinical impact with artificial intelligence. BMC Med. (2019) 17:195. doi: 10.1186/s12916-019-1426-2, PMID: 31665002 PMC6821018

[246] Vasey B Nagendran M Campbell B Clifton DA Collins GS Denaxas S . Reporting guideline for the early-stage clinical evaluation of decision support systems driven by artificial intelligence: Decide-Ai. Nat Med. (2022) 28:924–33. doi: 10.1038/s41591-022-01772-9, PMID: 35585198

[247] Rajpurkar P Chen E Banerjee O Topol EJ . Ai in health and medicine. Nat Med. (2022) 28:31–8. doi: 10.1038/s41591-021-01614-0, PMID: 35058619

[248] Sendak MP D'Arcy J Kashyap S Gao M Nichols M Corey KM . A path for translation of machine learning products into healthcare delivery. EMJ Innov. (2020). doi: 10.33590/emjinnov/19-00172

[249] Shah NH Milstein A Bagley Ph DS . Making machine learning models clinically useful. JAMA. (2019) 322:1351–2. doi: 10.1001/jama.2019.10306, PMID: 31393527

[250] Wiens J Saria S Sendak M Ghassemi M Liu VX Doshi-Velez F . Do no harm: A roadmap for responsible machine learning for health care. Nat Med. (2019) 25:1337–40. doi: 10.1038/s41591-019-0548-6, PMID: 31427808

[251] ClinicalTrials.gov [Internet]. Bethesda (MD): National Library of Medicine (2025). Available online at: https://clinicaltrials.gov/search?cond=photodynamic%20therapy&intr=artificial%20intelligence (Accessed January 21, 2025).

[252] Christie JR Lang P Zelko LM Palma DA Abdelrazek M Mattonen SA . Artificial intelligence in lung cancer: bridging the gap between computational power and clinical decision-making. Can Assoc Radiol J. (2021) 72:86–97. doi: 10.1177/0846537120941434, PMID: 32735493

[253] Olaye IM Seixas AA . The gap between Ai and bedside: participatory workshop on the barriers to the integration, translation, and adoption of digital health care and Ai startup technology into clinical practice. J Med Internet Res. (2023) 25:e32962. doi: 10.2196/32962, PMID: 37129947 PMC10189623

[254] United States. Department of H, Human Services ib United States F, Drug Administration ib United States F, Drug Administration Office of Combination Products ib . Principles of Premarket Pathways for Combination Products : Guidance for Industry and Fda Staff. Silver Spring, MD: Center for Drug Evaluation and Research (2022).

[255] U.S. Food and Drug Administration . Frequently Asked Questions About Combination Products. Silver Spring (MD): FDA (2020). Available online at: https://www.fda.gov/combination-products/about-combination-products/frequently-asked-questions-about-combination-products (Accessed January 21, 2025).

[256] Gunaydin G Gedik ME Ayan S . Photodynamic therapy for the treatment and diagnosis of cancer-a review of the current clinical status. Front Chem. (2021) 9:686303. doi: 10.3389/fchem.2021.686303, PMID: 34409014 PMC8365093

[257] Gomes-da-Silva LC Kepp O Kroemer G . Regulatory Approval of Photoimmunotherapy: Photodynamic Therapy That Induces Immunogenic Cell Death. OncoImmunology. (2020) 9:1841393. doi: 10.1080/2162402X.2020.1841393, PMID: 33178498 PMC7595598

[258] Kataoka H Nishie H Hayashi N Tanaka M Nomoto A Yano S . New photodynamic therapy with next-generation photosensitizers. Ann Transl Med. (2017) 5:183. doi: 10.21037/atm.2017.03.59, PMID: 28616398 PMC5464935

[259] U.S. Food and Drug Administration . Product Classification Database: Product Code Mvf - System, Laser, Photodynamic Therapy. Silver Spring (MD): FDA (2025). Available online at: https://www.accessdata.fda.gov/scripts/cdrh/cfdocs/cfPCD/classification.cfm (Accessed January 21, 2025).

[260] U.S. Food and Drug Administration . Photofrin (Porfimer Sodium) Prescribing Information. Silver Spring (MD): U.S. Food and Drug Administration (2020). Available from: https://www.accessdata.fda.gov/drugsatfda_docs/label/2020/020451s029,021525s005lbl.pdf.

[261] Calzavara-Pinton PG Rossi MT Aronson E Sala R Italian Group For Photodynamic T . A retrospective analysis of real-life practice of off-label photodynamic therapy using methyl aminolevulinate (Mal-Pdt) in 20 Italian dermatology departments. Part 1: inflammatory and aesthetic indications. Photochem Photobiol Sci. (2013) 12:148–57. doi: 10.1039/c2pp25124h, PMID: 22949035

[262] U.S. Food and Drug Administration . How to Write a Request for Designation (Rfd): Guidance for Industry. Silver Spring (MD): U.S. Food and Drug Administration (2011). Available online at: https://www.fda.gov/regulatory-information/search-fda-guidance-documents/how-write-request-designation-rfd.

[263] International Medical Device Regulators Forum (IMDRF) Software as a Medical Device (SaMD) Working Group . Software as a Medical Device: Possible Framework for Risk Categorization and Corresponding Considerations. IMDRF/SaMD WG/N12 FINAL:2014; 18 September 2014. Available from: https://www.imdrf.org/sites/default/files/docs/imdrf/final/technical/imdrf-tech-140918-samd-framework-risk-categorization-141013.pdf.

[264] U.S. Food and Drug Administration . Artificial Intelligence - Enabled Medical Devices Silver Spring (MD): FDA (2025). Available online at: https://www.fda.gov/medical-devices/software-medical-device-samd/artificial-intelligence-enabled-medical-devices (Accessed January 21, 2025).

[265] Windecker D Baj G Shiri I Kazaj PM Kaesmacher J Grani C . Generalizability of Fda-approved Ai-enabled medical devices for clinical use. JAMA Netw Open. (2025) 8:e258052. doi: 10.1001/jamanetworkopen.2025.8052, PMID: 40305017 PMC12044510

[266] Babic B Gerke S Evgeniou T Cohen IG . Algorithms on Regulatory Lockdown in Medicine. Science. (2019) 379:1202–4. doi: 10.1126/science.aay9547, PMID: 31806804

[267] U.S. Food and Drug Administration . Artificial Intelligence-Enabled Device Software Functions: Lifecycle Management and Marketing Submission Recommendations: Draft Guidance for Industry and Food and Drug Administration Staff. Silver Spring (MD): U.S. Food and Drug Administration (2025). Available from: https://www.fda.gov/regulatory-information/search-fda-guidance-documents/artificial-intelligence-enabled-device-software-functions-lifecycle-management-and-marketing (Accessed January 7, 2025).

[268] Wu E Wu K Daneshjou R Ouyang D Ho DE Zou J . How medical Ai devices are evaluated: limitations and recommendations from an analysis of Fda approvals. Nat Med. (2021) 27:582–4. doi: 10.1038/s41591-021-01312-x, PMID: 33820998

[269] U.S. Food and Drug Administration . Marketing Submission Recommendations for a Predetermined Change Control Plan for Artificial Intelligence-Enabled Device Software Functions: Guidance for Industry and FDA Staff. Silver Spring (MD): U.S. Food and Drug Administration (2025). Available from: https://www.fda.gov/regulatory-information/search-fda-guidance-documents/marketing-submission-recommendations-predetermined-change-control-plan-artificial-intelligence (Accessed August 18, 2025).

[270] Zhang SW Li YJ Liu WX Chu Q Wang SS Li JY . A decade of review in global regulation and research of artificial intelligence medical devices (2015-2025). Front Med. (2025) 12:1630408. doi: 10.3389/fmed.2025.1630408, PMID: 40747091 PMC12310608

[271] Price WN 2nd Gerke S Cohen IG . Potential liability for physicians using artificial intelligence. JAMA. (2019) 322:1765–6. doi: 10.1001/jama.2019.15064, PMID: 31584609

[272] Maliha G Gerke S Cohen IG Parikh RB . Artificial intelligence and liability in medicine: balancing safety and innovation. Milbank Q. (2021) 99:629–47. doi: 10.1111/1468-0009.12504, PMID: 33822422 PMC8452365

[273] Obermeyer Z Powers B Vogeli C Mullainathan S . Dissecting racial bias in an algorithm used to manage the health of populations. Science. (2019) 366:447–53. doi: 10.1126/science.aax2342, PMID: 31649194

[274] Price WN 2nd Cohen IG . Privacy in the age of medical big data. Nat Med. (2019) 25:37–43. doi: 10.1038/s41591-018-0272-7, PMID: 30617331 PMC6376961

[275] Koutsoubis N Waqas A Yilmaz Y Ramachandran RP Schabath MB Rasool G . Privacy-preserving federated learning and uncertainty quantification in medical imaging. Radiol Artif Intell. (2025) 7:e240637. doi: 10.1148/ryai.240637, PMID: 40366260 PMC12319697

[276] Bujotzek MR Akunal U Denner S Neher P Zenk M Frodl E . Real-world federated learning in radiology: hurdles to overcome and benefits to gain. J Am Med Inform Assoc. (2025) 32:193–205. doi: 10.1093/jamia/ocae259, PMID: 39455061 PMC11648732

[277] Gaube S Suresh H Raue M Merritt A Berkowitz SJ Lermer E . Do as Ai say: susceptibility in deployment of clinical decision-aids. NPJ Digit Med. (2021) 4:31. doi: 10.1038/s41746-021-00385-9, PMID: 33608629 PMC7896064

[278] Cai CJ Winter S Steiner D Wilcox L Terry M ed. “ Hello Ai” uncovering the onboarding needs of medical practitioners for human-Ai collaborative decision-making. Proc ACM Hum-Comput Interact. (2019) 3(CSCW):104. doi: 10.1145/3359206, PMID: 40727313

[279] Dwivedi R Dave D Naik H Singhal S Omer R Patel P . Explainable Ai (Xai): core ideas, techniques, and solutions. ACM Computing Surveys. (2023) 55:1–33. doi: 10.1145/3561048, PMID: 40727313

[280] Gunning D Stefik M Choi J Miller T Stumpf S Yang GZ . Xai-explainable artificial intelligence. Sci Robot. (2019) 4:eaay7120. doi: 10.1126/scirobotics.aay7120, PMID: 33137719

[281] Hossain MI Zamzmi G Mouton PR Salekin MS Sun Y Goldgof D . Explainable Ai for medical data: current methods, limitations, and future directions. ACM Computing Surveys. (2025) 57:1–46. doi: 10.1145/3637487, PMID: 40727313

[282] Hulsen T . Explainable artificial intelligence (Xai): concepts and challenges in healthcare. AI. (2023) 4:652–66. doi: 10.3390/ai4030034, PMID: 41725453

[283] Tjoa E Guan C . A survey on explainable artificial intelligence (Xai): toward medical Xai. IEEE Trans Neural Networks Learn Syst. (2020) 32:4793–813. doi: 10.1109/TNNLS.2020.3027314, PMID: 33079674

[284] van der Velden BHM Kuijf HJ Gilhuijs KGA Viergever MA . Explainable artificial intelligence (Xai) in deep learning-based medical image analysis. Med Image Anal. (2022) 79:102470. doi: 10.1016/j.media.2022.102470, PMID: 35576821

[285] Borys K Schmitt YA Nauta M Seifert C Krämer N Friedrich CM . Explainable Ai in medical imaging: an overview for clinical practitioners–beyond saliency-based Xai approaches. Eur J Radiol. (2023) 162:110786. doi: 10.1016/j.ejrad.2023.110786, PMID: 36990051

[286] Cerekci E Alis D Denizoglu N Camurdan O Ege Seker M Ozer C . Quantitative evaluation of saliency-based explainable artificial intelligence (Xai) methods in deep learning-based mammogram analysis. Eur J Radiol. (2024) 173:111356. doi: 10.1016/j.ejrad.2024.111356, PMID: 38364587

[287] Keles A Akcay O Kul H Bendechache M . Saliency maps as an explainable Ai method in medical imaging: A case study on brain tumor classification. Zenodo. (2023). doi: 10.5281/zenodo.8199332, PMID: 41863333

[288] Reddy S Allan S Coghlan S Cooper P . A governance model for the application of Ai in health care. J Am Med Inform Assoc. (2020) 27:491–7. doi: 10.1093/jamia/ocz192, PMID: 31682262 PMC7647243

